# Perilla Seed Oil Alleviates High-Fat-Diet-Induced Hyperlipidemia by Regulating Fatty Acid Metabolism via the PI3K/Akt/NOS3 Pathway

**DOI:** 10.3390/foods14234125

**Published:** 2025-12-01

**Authors:** Jianfeng Chang, Peng Hu, Bo Zhang, Yitong Liu, Yuting Cheng, Lianzhen Li, Leyuan Li

**Affiliations:** College of Agronomy, Henan Agricultural University, Zhengzhou 450046, China

**Keywords:** perilla seed oil, serum metabolomics, network pharmacology, PI3K-Akt, lipid-lowering mechanism

## Abstract

Perilla seed oil (PSO), rich in alpha-linolenic acid (ALA), has been traditionally used to relieve exterior syndrome and promote digestion, with modern studies confirming its anti-hyperlipidemic and anti-atherosclerotic properties. This study investigated the lipid-lowering effects of PSO and its underlying mechanisms in high-fat-diet-induced hyperlipidemic rats. Chemical standardization by UPLC-MS and GC-MS identified 591 compounds in PSO, with ALA accounting for 57.5% of its composition. The PSO administration significantly improved the general condition of hyperlipidemic rats, reduced body weight, lowered serum total cholesterol and LDL-C levels, and alleviated liver tissue injury and lipid accumulation. Serum metabolomics analysis revealed that PSO upregulated ALA and eicosapentaenoic acid while downregulating pro-inflammatory metabolites, including arachidonic acid, prostaglandin H2, and prostaglandin E2. Integrated network pharmacology and molecular docking studies identified the PI3K/Akt/NOS3 pathway as the primary signaling mechanism, which was further confirmed by Western blot analysis showing that PSO upregulated expression of p-PI3K, p-Akt, and NOS3 proteins. These results demonstrated that PSO-ameliorated hyperlipidemia, through PI3K/Akt/NOS3 pathway activation, coordinately modulated fatty acid metabolism and endogenous inflammatory responses. Our findings provided scientific evidence supporting PSO as a dietary intervention for managing hyperlipidemia and related metabolic disorders.

## 1. Introduction

Hyperlipidemia is becoming an increasingly serious threat to public health that co-locates with other disorders, including atherosclerosis, and non-alcoholic fatty liver disease (NAFLD) [1,2]. A high-fat diet (HFD) dysregulates blood lipids by increasing total cholesterol (TC), triglycerides (TG), and low-density lipoprotein cholesterol (LDL-C), in contrast to decreasing high-density lipoprotein cholesterol (HDL-C), causing abnormal lipid metabolism and eventually hyperlipidemia [3]. According to the World Health Statistics Report 2025 and related epidemiological studies, approximately 1.5 billion adults worldwide exhibit abnormal lipid levels [4,5]. With ongoing globalization and the adoption of Westernized dietary patterns, the prevalence of hyperlipidemia is increasingly rising in low- and middle-income countries, further straining healthcare systems.

Hyperlipidemia pathogenesis is multifactorial, involving nutritional status, lifestyle, and socio-economic determinants. Multiple interconnected signaling pathways underlie the complex pathophysiology of hyperlipidemia, orchestrating disruptions in glucose homeostasis, lipid and protein metabolism, as well as energy balance. The PI3K/AKT pathway serves as a central regulator of metabolism, coordinating lipid biosynthesis and lipolysis in metabolic tissues to maintain energy balance [6,7]. Its activation under normal conditions enhances glucose utilization and lipid storage, thereby supporting metabolic homeostasis and counteracting insulin resistance [6]. However, this pathway is inhibited by chronic energy surplus [8], which contributes to metabolic dysregulation. Consequently, the targeted activation of PI3K has emerged as a viable approach for treating obesity and hyperlipidemia by restoring metabolic equilibrium.

Growing evidence indicates that a healthy diet and behavioral interventions are considered a cornerstone in the initial management of obesity and associated diseases [9]. Among food sources, those that are less than 30% total fat and less than 10% saturated fat, alongside the replacement of animal fats with vegetable oils, are regarded as a healthy diet [10,11]. Furthermore, unsaturated fatty acids in one’s diet have been proven to be able to beneficially modulate lipid profiles and confer vascular protection [12]. Interestingly, α-linolenic acid (ALA) is an essential polyunsaturated fatty acid and, besides being the precursor for the omega-3 fatty acids eicosapentaenoic acid (EPA) and docosahexaenoic acid (DHA), is also involved in the regulation of lipid metabolism [13], reducing blood viscosity, enhancing oxygen transport, and exerting antioxidant and anti-inflammatory effects.

Perilla (*Perilla frutescens* (L.) Britt.), a member of the Lamiaceae family, is a traditional multi-purpose annual short-day plant, and has had a 2000-year history of use in China for food, medicine, spices, and cosmetics, before its utilization spread to Republic of Korea and Japan. Perilla seed is the dried ripe fruit of *Perilla frutescens*. In the “Ben Cao Hua Yi”, from the Ming Dynasty, it is recorded: “Perilla seed governs descending, with a pungent flavor and fragrant property that governs diffusion. It is used to resolve phlegm and dissipate nodules”. Within the theoretical framework of Traditional Chinese Medicine (TCM), hyperlipidemia falls under the category of “accumulation of greasy lipids and internal encumbrance of phlegm-turbidity” [14,15]. Therefore, the lipid-lowering effect of perilla seeds can be understood as stemming from and corroborating its traditionally documented functions in ancient texts, such as “resolving phlegm”, “directing Qi of the body downward flow”, and “moistening the intestines” [15].

The most valuable component of perilla seeds is their abundant oil. Modern research indicates that perilla seed oil (PSO) is an excellent source of polyunsaturated fatty acids, constituting 70.7–83.4% of its total content. Notably, the alpha-linolenic acid (ALA) content is remarkably high, ranging from 54% to 64%. Other constituents include linoleic acid (9.5–14.4%), oleic acid (9.6–20.8%), palmitic acid (5.1–7.0%), and stearic acid (1.1–2.3%) [16]. PSO has the highest ALA content compared to other plant seed oils. Acidic and enzymatic pre-treatments prior to cold-pressing have been shown to markedly increase seed-oil yield and transfer of phenolics without compromising oxidative stability [17,18]. Previous reports have indicated that PSO can inhibit hepatic lipid synthesis and regulate oxidative stress by reducing malondialdehyde (MDA) and protein carbonyl (PC) levels, while increasing hepatic glutathione (GSH) content and superoxide dismutase (SOD) activity [19,20,21,22]. Moreover, research has indicated that PSO is safe and nontoxic, as well as categorized as under the “no observed adverse effect level”. PSO can prevent atherosclerosis and colon cancer, and improve one’s health, such as the immune and mental function improvements demonstrated in animal studies and clinical trials [23,24]. Thus, Japan and the United States have developed PSO as a functional food ingredient. The efficacy of PSO in ameliorating HFD-induced hyperlipidemia in rats and promoting cardiovascular and cerebrovascular health has been established in numerous studies, which attribute these benefits to its high concentration of PUFAs and other bioactive compounds [25,26]. However, the underlying mechanism of hypolipidemic effects remains underexplored.

Metabolomics [27,28,29], which enables the comprehensive profiling of endogenous low-molecular-weight metabolites in biological systems, has emerged as a powerful tool for uncovering dynamic metabolic perturbations and pathway alterations in disease states and in response to interventions. Network pharmacology [27,30], on the other hand, adopts a holistic “multi-component, multi-target, multi-disease” perspective, utilizing high-throughput data integration and network analysis to decipher complex interactions between bioactive compounds and biological systems. Previous studies have evaluated the biological activity of PSO or ALA-rich vegetable oils using metabolomics or molecular pathway analysis [28]. In this study, we utilized UPLC-MS and GC-MS to systematically identify potential bioactive components in PSO. Furthermore, an integrated analysis combining network pharmacology and metabolomics, along with in vivo animal experiments, was employed to investigate the potential metabolic mechanism behind PSO’s anti-hyperlipidemic activity.

## 2. Materials and Methods

### 2.1. Materials and Reagents

#### 2.1.1. Animal Experiment Materials

A total of 70 male Sprague-Dawley rats (weighing approximately 200 g), specific pathogen-free (SPF) grade, were purchased from Liaoning Changsheng Biotechnology Co., Ltd. (Benxi, China; License No. SCXK (Liao) 2020-0002). The animal experiment was approved by the Henan Agricultural University’s Animal Welfare and Ethics Committee (Ethical Review Approval No. HNND2025110601, 18 May 2025). All the animals were conventionally housed at the Experimental Animal Center of Henan Agricultural University. The high-fat diet (HFD) was formulated to contain 636 g of basal feed, 200 g of sucrose, 150 g of lard, 12 g of cholesterol, and 2 g of sodium cholate per 1000 g of diet.

#### 2.1.2. Plant Experiment Materials

*Perilla frutescens* seeds, obtained from the cultivation base of Yiming Chinese Medicinal Materials Co., Ltd. (Yucheng County, Shangqiu, China), were used for the extraction of PSO. The plant material was authenticated by Professor Lianzhen Li of Henan Agricultural University (Zhengzhou, China) and was confirmed as *Perilla frutescens* var. acuta. The detailed specifications of all the materials provided by the manufacturers are listed in Supplementary Table S1.

### 2.2. UHPLC-MS Analysis of Chemical Composition of PSO

#### 2.2.1. Extraction of PSO

PSO was obtained through a multi-step process including seed cleaning, cold-pressing, filtration, and solvent extraction. Specifically, PSO was first extracted by cold pressing using a screw press at room temperature. The pressing temperature was 50 ± 10 °C, and the resulting oil temperature was 39 ± 1 °C. The crude oil was then filtered through fine gauze and subsequently subjected to sequential liquid–liquid extractions with 0.1 mol/L NaCl solution for 24 h, and then with distilled water for 12 h, with the upper oil layer retained after each step. Finally, the PSO was stored in dark glass containers at 4 °C for later use.

#### 2.2.2. UHPLC-MS Analysis and Data Processing

A 100 µL aliquot of PSO was mixed with 400 µL of ice-cold methanol:acetonitrile (1:1, *v*/*v*) containing isotope-labeled internal standards. The mixture was then vortex-mixed, sonicated for 10 min in an ice-water bath (SCIENTZ SB-5200 DTD, Ningbo, China), and incubated at −40 °C for 1 h according to the previous description [31]. The resulting supernatant was filtered prior to instrumental analysis.

The chemical compounds were chromatographically separated using a Vanquish (Thermo Fisher Scientific, Waltham, MA, USA) ultra-high-performance liquid chromatography system (UHPLC) with a Phenomenex Kinetex C18 column (2.1 mm × 50 mm, 2.6 μm; Agilent Technologies, Santa Clara, CA, USA). Mobile phase A was aqueous, containing 0.01% acetic acid; phase B was isopropanol: acetonitrile (1:1, *v/v*). The metabolites were eluted as follows: 0–0.5 min, 99% A; 0.5–4.0 min, 99–1% A; 4.0–4.5 min, 1% A; 4.5–4.55 min, 1–99% A; 4.55–6.0 min, 99% A. The column temperature was 25 °C, the sample tray temperature was 4 °C, the injection volume was 2 μL, and the flow rate was 0.3 mL/min. The Orbitrap Exploris 120 mass spectrometer acquired primary and secondary mass spectrometry data under control software (Xcalibur, Version: 4.4, Thermo Fisher Scientific, Waltham, MA, USA). Detailed parameters are as follows: Sheath gas flow rate: 50 Arb, Aux gas flow rate: 15 Arb, Capillary temperature: 320 °C, Sweep Gas: 1 Arb, Vaporizer Temp: 350 °C. Full mass resolution: 60,000, MS/MS resolution: 15,000, Collision energy: SNCE 20/30/40, Spray Voltage: 3.8 kV (positive) or −3.4 kV (negative). The data were converted to the mzXML format using ProteoWizard software, the BiotreeDB (V3.0, reference standard database), and BT-Plant (V1.1, plant-specific database). Visualization analysis was then performed using a custom-developed R package (Version 4.3.2).

### 2.3. Determination of Major Fatty Acids in PSO by GC-MS/MS

#### 2.3.1. Sample Preparation

Fatty acids analysis was conducted via a methylation reaction, referring to previous report [32]. Briefly, 0.3 g of PSO was blended with 4 mL of isooctane and agitated on a shaker at 37 °C for 8 h to achieve full homogenization. Next, 4 mL of a 2 mmol/L potassium hydroxide–methanol solution was introduced and mixed. Following a resting period, sodium bisulfate was applied to counteract any residual potassium hydroxide. The resulting supernatant was filtered and diluted with isooctane in readiness for GC-MS analysis.

#### 2.3.2. GC-MS/MS Analysis

The determination of fatty acid content was performed on a SUPEC7000 GC-MS/MS (EXPEC Technology, Hangzhou, China) system based on GB 5009.168-2016 [33]. Chromatographic separation was achieved on a SP-2560 capillary column (100 m × 0.25 mm, 0.2 μm) under the following conditions: helium carrier gas (≥99.999%) at 1.0 mL/min, a 1:1 split ratio, and an initial column temperature of 80 °C. The temperature was ramped up to 200 °C at a rate of 6 °C/min, then raised to 218 °C at 1 °C/min, further ramped up to 220 °C at 0.25 °C/min, and finally elevated to 230 °C at 1 °C/min, then held for 8 min. Electron ionization (EI) was employed for ion generation, with the ion source temperature set at 250 °C. The quantification of fatty acids in the samples was performed using the external standard method, and all fatty acid methyl esters showed good linearity in their linear ranges with correlation coefficients (R^2^) greater than 0.999. The limits of detection (LODs) of the method were in the range of 0.005–0.079 μg/mL, and the limits of quantification (LOQs) were between 0.018 and 0.264 μg/mL, with the results represented in grams per kilogram (g/kg) [34].

### 2.4. Network Pharmacology Analysis

The compounds analyzed in this study were collected from the analysis described in Section 2.2. (Supplementary Table S2). The potential compounds of PSO were collected based on UPLC-MS and GC-MS/MS spectrometry, with high abundance in our previous literature research. Potential targets of these compounds were screened against the Traditional Chinese Medicine Systems Pharmacology (TCMSP) database, with subsequent identity verification and standardization performed in the UniProt database.

#### 2.4.1. Identification of Hyperlipidemia-Associated Targets

Potential therapeutic targets for hyperlipidemia were screened by searching the keyword “Hyperlipidemia” in disease databases, such as DisGeNET Database (https://www.genecards.org/) and GeneCards (https://www.genecards.org). To identify the shared targets, a Venn diagram was generated to visualize the overlap between PSO’s active compound targets and those associated with hyperlipidemia.

#### 2.4.2. Construction of Protein–Protein Interaction Networks

A protein–protein interaction (PPI) network for the overlapping targets was constructed using the STRING database, with the organism set as *Homo sapiens* and a minimum confidence score threshold of 0.9. Following its construction, the network was analyzed in Cytoscape (Version 3.10.3) with the MCODE plugin.

#### 2.4.3. Gene Ontology (GO) and Kyoto Encyclopedia of Genes and Genomes (KEGG) Analysis

GO and KEGG enrichment analyses were conducted via the STRING database to identify hyperlipidemia-related pathways for PSO. Significantly enriched terms (Biological Process (BP), Cellular Component (CC), Molecular Function (MF)) and KEGG pathways (*p* ≤ 0.05) were selected and visualized as bubble charts on the bioinformatics platform (http://www.bioinformatics.com.cn/).

#### 2.4.4. Compound-Target-Pathway Networks

To delineate the therapeutic mechanism of PSO against hyperlipidemia, a compound-target-pathway network was built in Cytoscape 3.8.2, revealing its core constituents, pivotal targets, and central pathways.

#### 2.4.5. Molecular Docking Validation

Molecular docking simulations were performed between the selected ligands and the receptor using AutoDock Vina. The crystal structure of the target protein was downloaded from the PDB and prepared in AutoDock Vina 1.5.7, including the addition of hydrogen atoms and the assignment of partial charges. The optimal binding conformations between the key targets and the major active components were identified and visualized using PyMOL (Version 3.1.6.1).

### 2.5. Animal Experiments

Upon completion of the one-week acclimatization period, the rats were randomly allocated to five experimental groups (*n* = 10): a control group maintained on a standard diet; an HFD model group; and three HFD groups that received oral supplementation with PSO at low (5 g/kg/d), medium (10 g/kg/d), or high (15 g/kg/d) doses, respectively. The dietary intervention was conducted over 8 weeks, with weekly monitoring of body weight. Upon the completion of the intervention, the animals were euthanized by 1% sodium pentobarbital. Blood was drawn from the abdominal aorta, followed by centrifugation to obtain serum; liver tissue was also excised. Following collection, all samples were immediately snap-frozen in liquid nitrogen and stored at −80 °C for later use. Liver specimens destined for histological examination were fixed in 4% paraformaldehyde.

### 2.6. Serum Biochemical Parameter

TC, TG, HDL-C, and LDL-C in serum were measured using commercial assay kits from Nanjing Jiancheng Bioengineering Institute (Nanjing, China), with an automatic biochemical analyzer (GS 200; Shenzhen Jinrui Biotechnology Co., Ltd., Shenzhen, China).

### 2.7. Serum Metabolomics Analysis

Following a one-week acclimation period, the rats were randomly assigned into two groups (n = 10): a control group (administered water, 10 g/kg/d) and a PSO group (administered PSO, 10 g/kg/d). The treatments were orally gavaged once daily for 7 days. On day 7, after a 12 h fast (with free access to water), the rats were weighed, anesthetized with 1% sodium pentobarbital, and subjected to dissection for blood collection. Serum was separated, snap-frozen in liquid nitrogen, and stored at −80 °C for subsequent metabolomic analysis.

Serum samples (100 μL) were mixed with 400 μL of ice-cold extraction solvent (methanol:acetonitrile, 1:1, *v*/*v*). After vortexing at 750 rpm for 5 min and standing for an additional 5 min, the supernatant was collected for analysis. Chromatographic separation was carried out on a Vanquish UHPLC system (Thermo Fisher Scientific, Waltham, MA, USA) equipped with a Waters ACQUITY UPLC BEH Amide column (2.1 mm × 50 mm, 1.7 μm, Waters Corporation, Milford, MA, USA). Mobile phase A was aqueous, containing 25 mmol/L ammonium acetate and 25 mmol/L ammonia; phase B was isopropanol: acetonitrile (1:1, *v*/*v*). The contents are as follows: 0–0.25 min, 5% A; 0.25–3.5 min, 5–35% A; 3.5–4.0 min, 35–60% A; 4.0–4.5 min, 60% A; 4.5–4.55 min, 60% A; 4.55–6.0 min, 5% A. The column temperature was 25 °C, the sample tray temperature was 4 °C, the injection volume was 2 μL, and the flow rate was 0.5 mL/min. The Orbitrap Exploris 120 mass spectrometer acquired primary and secondary mass spectrometry data under control software (Xcalibur, Version: 4.4, Thermo Fisher Scientific, Waltham, MA, USA). The detailed parameters were outlined in the Methods section, Section 2.2.2. The raw data were converted to mzXML format using ProteoWizard (v3.0.24054) for further processing.

To ensure data quality, quality control (QC) samples were injected prior to the actual sample sequence to equilibrate the system. Metabolic features with excessive variation were filtered based on the relative standard deviation (RSD) of their peak areas in the QC samples. Only variables with missing values not exceeding 50% in any single group or 50% across all groups were retained. The qualified data were then normalized and subjected to principal component analysis (PCA) and orthogonal partial least squares-discriminant analysis (OPLS-DA). To control the false discovery rate, the *p*-values from Student’s *t*-test were adjusted using the Benjamini–Hochberg procedure. The final criteria for identifying differential metabolites were an FDR-adjusted *p*-value (q-value) < 0.05 and a variable importance in projection (VIP) score > 1 from the OPLS-DA model. Finally, the KEGG pathway enrichment analysis was performed on the identified differential metabolites.

### 2.8. Western Blot Analysis

Total proteins were extracted from the liver tissue using RIPA lysis buffer, quantified, and denatured at 95 °C. The protein concentration was then quantified using a BCA assay kit (Solarbio, Beijing, China) with a Synergy H1 microplate reader (BioTek Instruments Inc, Winooski, VT, USA). After separation by SDS-PAGE, the proteins were transferred to a PVDF membrane. The membrane was blocked and then incubated overnight at 4 °C with primary antibodies against p-PI3K, PI3K, p-AKT, AKT, NOS3, and GAPDH (all at 1:2000 dilution). Following washing, the membrane was incubated with an HRP-conjugated secondary antibody (1:10,000) at 37 °C for 1 h. Protein bands were visualized using a ChemiDoc Go System (BioTek Instruments Inc., Winooski, VT, USA), and the levels of target proteins were quantified.

### 2.9. Statistical Analysis

Statistical analysis was conducted with GraphPad Prism 8.0.2. Quantitative data are expressed as mean ± standard deviation (SD). Intergroup differences were determined by Student’s *t*-test (two groups) or one-way ANOVA with post hoc tests (multiple groups), with *p* < 0.05 considered statistically significant.

## 3. Results

### 3.1. Analysis of Chemical Components in PSO

**Figure 1:**
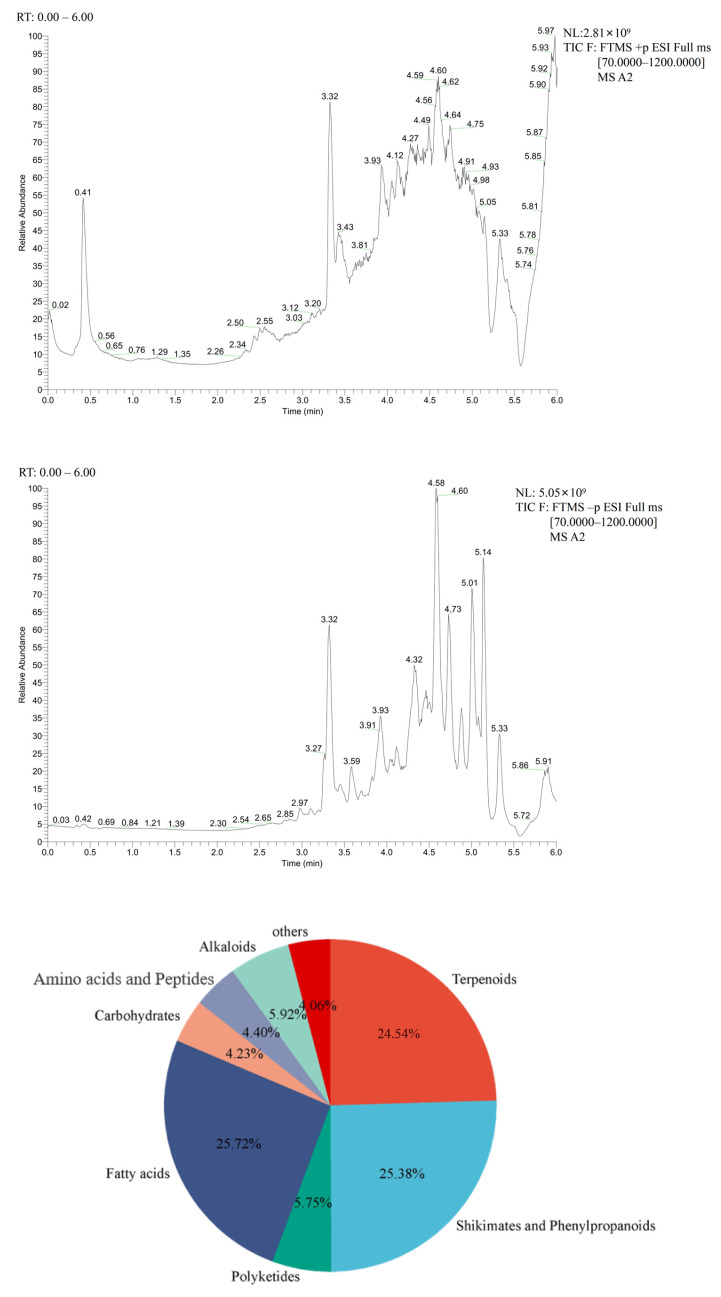
Chemical composition analysis of PSO. (**a**) Total ion chromatogram (TIC) of PSO in positive ion mode. (**b**) TIC of PSO in negative ion mode. The green line represents the retention time of compounds in PSO. (**c**) Pie chart illustrating the distribution of 591 chemical compounds into 8 classes.

The research has indicated that PSO contains a complex chemical composition, which consists of essential fatty acids, volatile organic compounds, monoterpenoids, phenolic compounds, pyrazine, aldehyde, and flavonoid compounds [35]. Furthermore, we confirmed the chemical composition of PSO using the UPLC-MS/MS in this study. The positive and negative ion flow profile for identifying compounds in the PSO are shown in Figure 1a,b. A total of 591 compounds were identified by comparative screening from the standard solution and collected database CD2.1 (Thermo Fisher Scientific, Waltham, MA, USA), and categorized into 8 different classes, including 152 fatty acids, 150 shikimates and phenylpropanoids, 145 terpenoids, 34 polyketides, 25 carbohydrates, 26 amino acids and peptides, and 35 alkaloids and others. Among these, the identified three categories of fatty acids, shikimates, phenylpropanoids, and terpenoids accounted for 75.63% of the total compounds (Figure 1c, Supplementary Table S2).

### 3.2. GC-MS/MS Fragmentation Profiling of PSO

**Figure 2:**
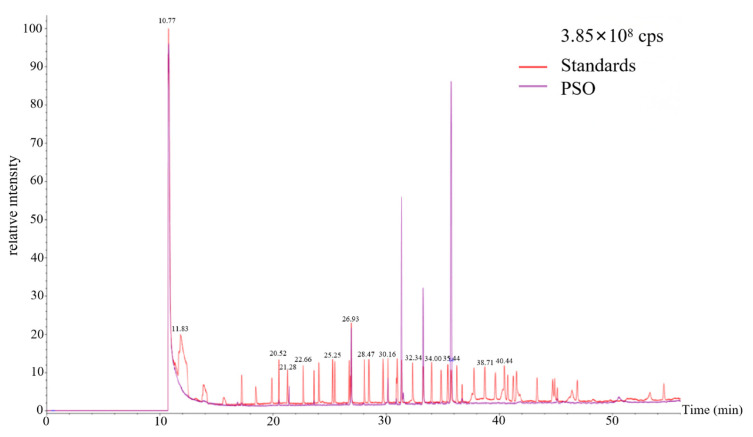
Representative GC–MS/MS chromatogram of PSO fatty acid methyl esters.

The fatty acid composition defines the nutritional value of vegetable oils. Previous studies have indicated that perilla seed oil (PSO) possesses a distinct profile, with unsaturated fatty acids (UFAs) representing 91.70% and saturated fatty acids (SFAs) comprising 8.34% of its total content [36]. In this study, the fatty acid methyl ester profile of PSO was characterized by GC-MS/MS, with the identification carried out through the comparison of key fragment ions from the sample against those of authentic FAME standards and the NIST mass spectral database (Figure 2). In total, 11 compounds were confirmed by matching both the standard and NIST library, including methyl myristoleate, methyl pentadecanoate, methyl palmitate, methyl heptadecanoate, methyl stearate, methyl oleate, methyl linoleate, methyl α-linolenate, methyl arachidonate, methyl gadoleate, and methyl docosanoate. Furthermore, the contents of these compounds and their linear calibration curve were quantitatively analyzed and calculated (Table 1, Supplementary Figure S1, and Supplementary Table S4). The major fatty acids were PUFAs of ALA and linoleic acid, and monounsaturated fatty acids of oleic acid and palmitic acid. Among these, ALA was the most abundant, representing approximately 57.5% of the total fatty acid content, consistent with previous reports [37].

### 3.3. Effects of PSO on Body Weight and Liver Function

**Figure 3:**
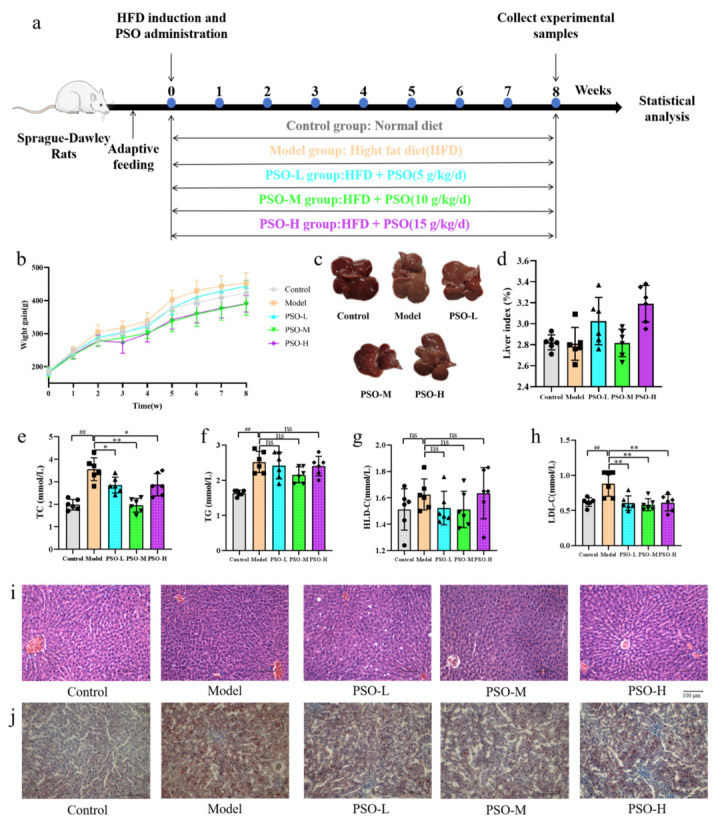
PSO ameliorates HFD-induced hyperlipidemic rats. (**a**) The animal experimental procedure design. The control group is represented in gray, the model group in orange, the PSO-L group in green, the PSO-M group in blue, and the PSO-H group in purple. (**b**) Body weight measurements; (**c**) image of liver; (**d**) liver organ index, representing the liver weight divided by body weight; (**e**) serum TC; (**f**) serum TG; (**g**) serum HLD-C; (**h**) serum LDL-C; (**i**) representative photomicrographs of H&E-stained liver sections; (**j**) hepatic lipid deposition visualized by Oil Red O staining. Data are presented as mean ± SD. ^##^ *p* < 0.01 vs. control group; * *p* < 0.05, ** *p* < 0.01 vs. model group; ns means *p* > 0.05 vs. control group or vs. model group.

An HFD model was established in SD rats using the feeding protocol illustrated in Figure 3a, followed by the corresponding drug interventions. Body weight was monitored weekly across all groups. As shown in Figure 3b, PSO supplementation suppressed HFD-induced body weight gain. Rats treated with medium- and high-dose PSO showed a mild reduction in body weight relative to controls, though the differences were not statistically significant through the ANOVA analysis (*p* > 0.05). At the end of the treatment period, liver tissues were collected, and the organ index was calculated (Figure 3c,d). PSO did not significantly affect the weight of liver organs, while the liver index of the PSO-M group was close to that of the control group.

To further assess the liver function of rats, we measured the physiological indexes, including TC, TG, LDL-C, and HDL-C, using ELISA kits after the end of gavage (Supplementary Table S5). PSO administration significantly ameliorated HFD-induced dyslipidemia. Specifically, the elevated hepatic TC and LDL-C levels in the model group were notably reduced following PSO treatment, with statistical significance (*p* < 0.05, *p* < 0.01), compared to the model group as per the ANOVA analysis (Figure 3e–h). These results confirmed that PSO has notable hypolipidemic activity in hyperlipidemic rat models.

### 3.4. PSO Alleviates Hepatic Steatosis in HFD-Fed Rats

Hepatic histology was assessed by H&E and Oil Red O staining after eight weeks of treatment to determine the anti-hyperlipidemic effect of PSO. The representative images are presented in Figure 3i,j, respectively. H&E staining revealed that compared with the control group, the model group exhibited disrupted hepatic lobular architecture, along with compressed and even occluded hepatic sinusoids. In contrast, PSO treatment groups showed dose-dependent improvements; the low-dose PSO group displayed some vacuolization, whereas medium- and high-dose groups demonstrated a notable reduction in vacuolization and a restoration of lobular structure. Consistent with these findings, Oil Red O staining indicated the severe lipid accumulation in the model group, which was significantly attenuated in all PSO-treated groups in a dose-responsive manner. Thus, histopathological analysis indicated that PSO exerted a protective effect on the liver tissue of rats.

### 3.5. PSO Improved the Lipid Metabolism Profile by Targeting PI3K-AKT

#### Signaling Pathway

**Figure 4:**
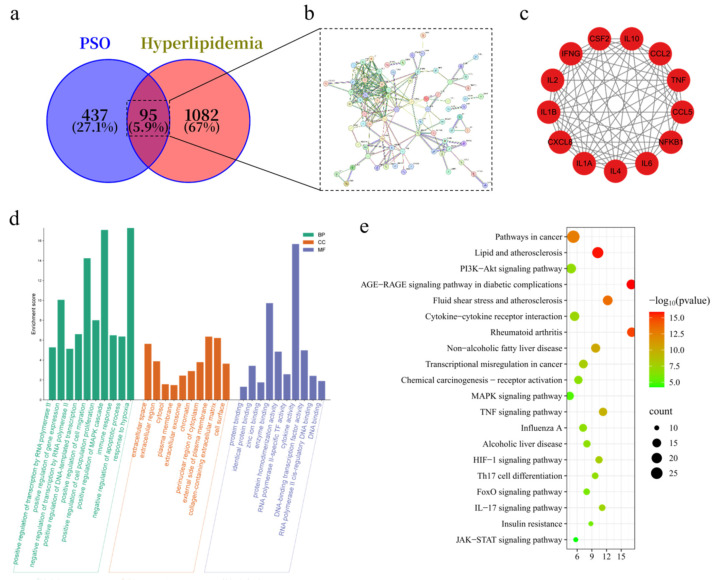
Network pharmacology analysis of PSO treating hyperlipidemia. (**a**) Venn diagram of intersection targets between PSO and hyperlipidemia. (**b**) Interaction relationship diagram of intersection targets. (**c**) PPI network diagram of key targets. (**d**) GO enrichment analysis. (**e**) KEGG enrichment analysis.

To elucidate the mechanism of PSO, a network pharmacology approach was applied. Screening of relevant databases yielded 532 targets for its bioactive compounds. From searching the keyword “hyperlipidemia” in GeneCards and DisGeNET databases, 1177 targets were acquired. As displayed in Figure 4a, there were 95 intersection biomarkers identified as potential therapeutic targets of PSO in treating hyperlipidemia. Subsequently (Supplementary Table S3), a PPI network of the common targets was generated using the STRING database and analyzed in Cytoscape (v3.9.1) to elucidate their interactive relationships, as depicted in Figure 4b. Then, we ultimately screened the core targets, including TNF, CCL5, IL6, NFKB1, and others, based on the MCODE score of 11.833 from the PPI network (Figure 4c).

GO enrichment analysis via the DAVID database categorized the targets into biological processes (BP, 493 terms), cellular components (CC, 42 terms), and molecular functions (MF, 82 terms). The top 10 significantly enriched terms for each category are presented in Figure 4d. The results showed that BP was most related to transcriptional regulation (especially RNA polymerase II-related), and immuno-inflammatory responses, CC was most related to the extracellular space and extracellular region, suggesting the key role of target gene populations in cell-to-cell communication and microenvironment regulation, and MF was most related to nuclear receptor activity and steroid nuclear receptor activity.

KEGG pathway analysis indicated significant alterations in metabolic pathways among the potential targets of PSO. The 95 targets were mapped to 119 significant pathways, and the top 20 (*p* < 0.05, ranked by gene count) are presented in Figure 4e. These pathways were used to build a “compound-target-pathway” network (Supplementary Figure S2). Overall, these data highlighted that the metabolic pathway alterations might be the potential targets of PSO when treating hyperlipidemia, especially the lipid and atherosclerosis pathway (hsa05417), PI3K-Akt signaling pathway (hsa04151), and AGE-RAGE signaling pathway (hsa04933).

### 3.6. Molecular Docking

**Figure 5:**
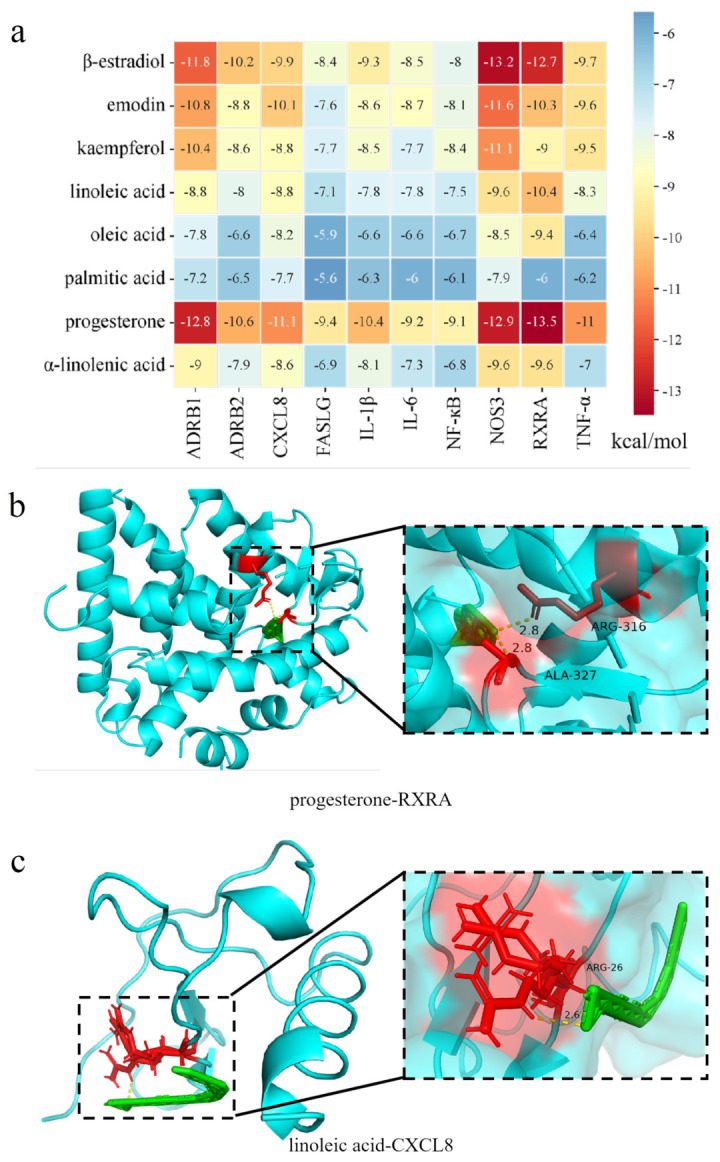
The result of molecular docking between the ligand and the receptor. (**a**) The heat map. (**b**,**c**) Partial molecular docking model of the core small molecule binding to the core protein: (**b**) progesterone−RXRA, green: progesterone, red: the amino acid structure where progesterone interacts with RXRA, and yellow dashed lines: the forces between progesterone and RXRA. (**c**) linoleic acid−CXCL8, green: linoleic acid, red: the amino acid structure where linoleic acid interacts with CXCL8, and yellow dashed lines: the forces between linoleic acid and CXCL8.

The top nine active compounds identified after filtering by the “compound-target-pathway” network included β-estradiol, emodin, kaempferol, progesterone, and four primary fatty acids: linoleic acid, oleic acid, palmitic acid, and α-linolenic acid. The top 10 core targets, including ADRB1, ADRB2, CXCL8, FASLG, IL-1β, IL-6, NF-κB, NOS3, RXRA, and TNF-α were also screened. Molecular docking was conducted between the nine core compounds and the ten hub gene-derived proteins, resulting in 90 distinct conformational complexes (Figure 5a). The molecular docking results indicate that a binding energy of less than −5 kcal/mol among all the compounds and the proteins in this study, and suggest a good and stable binding activity between the ligand and the receptor. Specifically, the retinoid x receptor alpha (RXRA)-progesterone complex had an affinity of −13.5 kcal/mol and formed two hydrogen bonds at the ARG-316 and ALA-327 residues (Figure 5b). The C-X-C Motif Chemokine Ligand 8 (CXCL8)-linoleic acid complex had an affinity of −8.8 kcal/mol and formed one hydrogen bond at the ARG-26 residues (Figure 5c).

### 3.7. PSO Affects Serum Metabolic Markers in Rats

**Figure 6:**
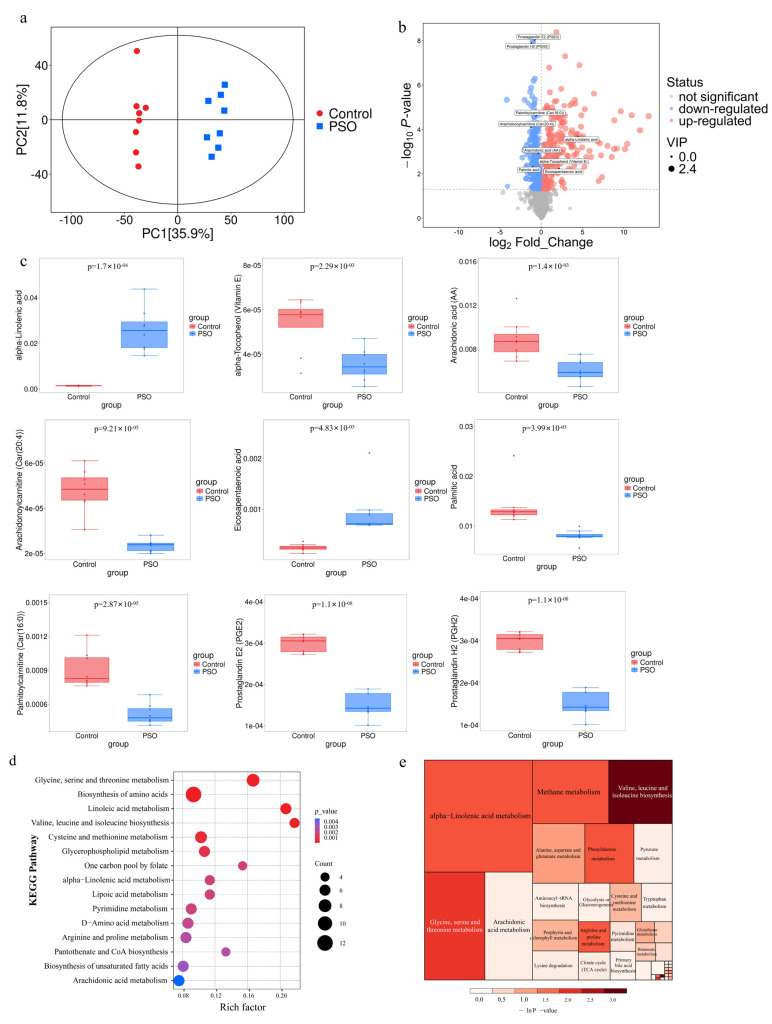
The analysis of the endogenous serum metabolites in PSO. (**a**) Principal component analysis. (**b**) Volcano plot. (**c**) Relative abundance of the differential metabolites between the control and PSO−treated groups. Data are presented as mean ± SD. *p* < 0.01 was considered statistically significant in the PSO−treated group compared with the control group. (**d**) Bubble chart of KEGG enrichment. (**e**) Comprehensive analysis heatmap of the differential metabolite pathways. The color of the square indicates the *p*-value of the enrichment analysis (expressed as −ln(p)); deeper colors correspond to smaller *p*-values, indicating greater enrichment significance.

UPLC-MS was used to investigate the serum metabolomics from the PSO-treated group and the control group. The Principal Component Analysis (PCA) score plot revealed the metabolic profiles of well-clustered samples from each group in unsupervised mode, and a robust separation was also presented between the two groups. The PSO-treated group was positioned closer to the normal group, indicating that PSO significantly altered the serum metabolic profile in rats (Figure 6a). Orthogonal partial least squares discriminant analysis (OPLS-DA) further confirmed the separation between the control and PSO groups, while the PSO group was more closely related to the control group, and further validated by the permutation test.

A total of 1985 serum metabolites were identified, of which 635 significant differential metabolites were ultimately identified following a preliminary screening using Student’s *t*-test (*p* < 0.05) and VIP > 1 from OPLS-DA. Compared with the control group, PSO administration resulted in the downregulation of 266 endogenous metabolites and the upregulation of 369 (Figure 6b). Ultimately, a total of 81 metabolites were identified, including 25 significantly upregulated and 56 significantly downregulated, based on the criterion of FDR < 0.05 (Supplementary Table S6). Notably, several key metabolites were significantly altered, including the upregulation of α-linolenic acid and eicosapentaenoic acid, and the downregulation of palmitic acid, arachidonic acid (AA), Prostaglandin H2 (PGH2), Prostaglandin E2 (PGE2), alpha-Tocopherol (Vitamin E), Arachidonoylcarnitine (Car(20:4)), and Palmitoylcarnitine (Car(16:0)) (Figure 6c, Supplementary Table S7). These changes may affect the metabolism, absorption, and utilization of PSO in rats, thereby treating hyperlipidemia. The KEGG pathway enrichment analysis of all differentially abundant metabolites highlighted significant alterations in amino acid metabolism (e.g., glycine, serine, and threonine metabolism) and fatty acid metabolism pathways (e.g., linoleic acid and α-linolenic acid metabolism) (Figure 6d). The subsequent comprehensive pathway analysis, which integrated both enrichment and topological assessment, identified the linolenic acid metabolism pathway as the key metabolic pathway in serum samples (Figure 6e).

### 3.8. PSO-Regulated PI3K/AKT/NOS3 Cellular Metabolic Signaling to Exert Hypolipidemic Effect

**Figure 7:**
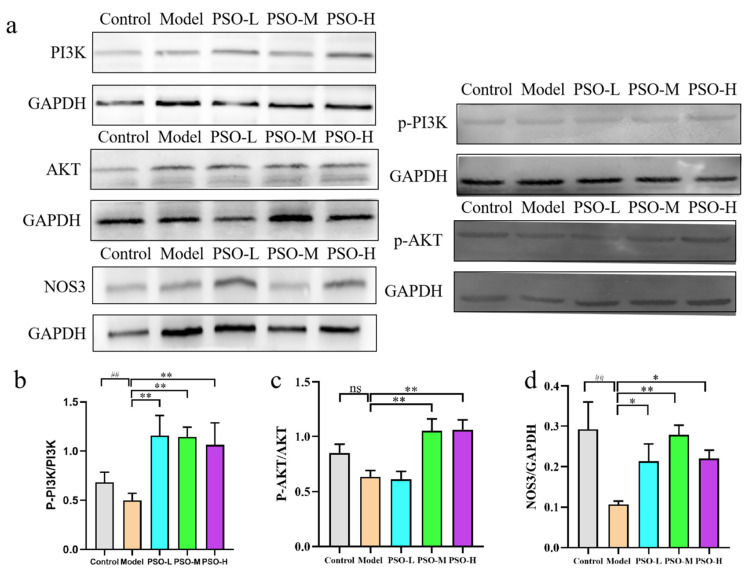
Western blot analysis of the expression of PI3K/AKT/NOS3 signaling pathway-related proteins. (**a**) Western blot assays were performed to detect the expression levels of p-PI3K, PI3K, p-AKT, AKT, and NOS3. The p-PI3K/PI3K (**b**), p-AKT/AKT (**c**), and NOS3/GAPDH (**d**) were calculated by grayscale analysis. Data are presented as mean ± SD (n = 3, with three independent biological replicates). ^##^ *p* < 0.01 vs. control group; * *p* < 0.05, ** *p* < 0.01 vs. model group; ns means *p* > 0.05 vs. control group or vs. model group.

To confirm that the PI3K-Akt signaling pathway is involved in the PSO-regulated lipid metabolism, we analyzed key components of the PI3K-Akt signaling pathway at the protein level, based on the integrated findings from the metabolomics and network pharmacology analyses (Figure 7a, Supplementary Figure S3, and Supplementary Table S8). The statistical analysis of the Western blot data using ANOVA revealed that the phosphorylation levels of PI3K and AKT, as well as the NOS3 expression relative to GAPDH, were significantly decreased in the model group relative to controls (*p* < 0.05, *p* < 0.01). Notably, NOS3 protein levels in the model group were reduced by 2-fold compared with the control group. In contrast, PSO treatment significantly upregulated these markers compared to the model group (*p* < 0.05, *p* < 0.01) (Figure 7b–d). Thus, the results suggested that PSO can promote lipid metabolism signaling by activation of the PI3K/AKT/NOS3 pathway in the liver of hyperlipidemic rats.

## 4. Discussion

Hyperlipidemia is a systemic metabolic disorder with an increasing prevalence, which represents a major underlying risk factor for cardiovascular diseases (CVD) and increases societal healthcare costs [38,39,40]. Consequently, the prevention or early intervention of hyperlipidemia is of paramount clinical importance. Current therapeutic strategies often involve the use of statins, sometimes in combination with lipid-regulating traditional Chinese medicines (TCM) [14]. Recently, the PSO from *P. frutescens* has been attracting attention as a health food product, and the cultivation area of cultivated *P. frutescens* has also expanded significantly in China and Republic of Korea. PSO has emerged as a promising functional edible vegetable oil with essential fatty acids to balance the ω-6/ω-3 PUFA ratio in one’s diet, and serve as a natural, nontoxic ingredient in food products. It has been shown that PSO could provide health promotional effects, reduce cholesterol, regulate blood pressure, attenuate ecological dysregulation, prevent cancer, provide anti-aging effects, etc. [41,42]. Previous studies have shown that dietary PSO supplementation relieves hyperglycemia induced by an HFD and attenuates metabolic disorders in the gut [43,44]. However, given the complex chemical composition of PSO, a systematic investigation is required to identify the specific bioactive constituents responsible for its antihyperlipidemic activity and to elucidate potential synergistic effects.

In this study, a total of 591 compounds were identified in PSO, comprising 152 fatty acids, 150 shikimates and phenylpropanoids, 145 terpenoids, and 34 polyketides. The major fatty acids were identified and quantified through GC-MS/MS, with ALA as the predominant component, accounting for 57.5% of PSO’s composition, and establishing a material basis for its bioactivity. This result indicated that PSO contained 57.5% ALA content more than siritch oil, peony seed oil, and herbaceous peony seed oil reported in previous studies of edible vegetable oils [45,46]. As a member of the ω-3 PUFAs family, ALA could be converted into EPA and DHA by desaturase and elongase enzymes in mammals, though the conversion rate ranges from 0.3% to 10% [47,48,49], promoting the development of the brain and retina.

A hyperlipidemic rat model was successfully established through an HFD. Compared to the control group, model rats exhibited significantly elevated serum levels of TC, TG, and LDL-C (*p* < 0.05, *p* < 0.01). Subsequent intervention with PSO significantly reduced TC and LDL-C levels across all dosage groups (*p* < 0.05, *p* < 0.01), thereby confirming its hypolipidemic efficacy, which is consistent with previous findings [40]. However, TG and HDL-C levels did not show a significant difference, which may reflect the short-term (8-week) intervention effects, and longer-term intervention requires further investigation. In addition, the administration of PSO (5, 10, and 15 g/kg/d) could effectively attenuate pathological dysregulation, lipid accumulation, and inflammation in the liver tissue of hyperlipidemic rats induced by an HFD. In addition, it has been proven that elevated levels of native LDL in the model rats were accompanied by decreased levels of endothelial eNOS [50], which is consistent with verified results by Western blot.

Metabolomics research is an interdisciplinary field that systematically investigates the dynamic changes in endogenous small molecule metabolites within biological systems and in the corresponding organismal responses, and obtains potential biomarkers more quickly and accurately. This approach provides critical insights for drug development and personalized medicine, which is also an important embodiment of the holistic view of TCM [51,52]. Using untargeted metabolomics analysis, we examined the impact of PSO on the serum metabolic profile of rats. The PSO-treated group showed significant upregulation of metabolites such as ALA and EPA, whereas the levels of AA, PGH2, PGE2, and several acylcarnitines were markedly downregulated. Both ω-6 and ω-3 PUFAs are essential precursors for the synthesis of prostaglandins, leukotrienes, and structural components of cell membranes and phospholipids. They play vital roles in visual function, brain tissue integrity, and reproductive health [53,54].

In particular, the ω-3 PUFAs family of ALA is known to exert crucial regulatory effects on the cardiovascular, immune, nervous, and digestive systems [55,56]. Given its high ALA content, dietary supplementation with PSO may contribute to immune enhancement and improved digestive function. PGH2 and PGE2 are lipid mediators belonging to the prostaglandin family, known for their roles in inflammation, allergic responses, and pain perception [57]. Previous studies have indicated that fish oil can decrease TC, TG, apolipoprotein B, and glucose, and these reductions were positively correlated with decreases in serum PGE2 [58]. The observed reduction in serum levels of PGH2 and PGE2, following PSO intervention, suggests a potential anti-inflammatory mechanism of PSO. Meanwhile, the KEGG pathway enrichment analysis further indicated that PSO modulates several core metabolic pathways, including α-linolenic acid metabolism, glycerophospholipid metabolism, and arachidonic acid metabolism. Notably, PSO significantly regulated key differential metabolites by reducing pro-inflammatory AA and lysophosphatidylcholine levels. Thus, the serum metabolomics in HFD rats indicated that PSO may regulate fatty acid metabolism networks and endogenous inflammatory factors to reduce the lipid level in HFD rats.

To systematically elucidate the multi-target mechanism of PSO, we employed a network pharmacology approach to identify potential active ingredients and targets associated with its anti-hyperlipidemic effects. This suggested that alpha-linolenic acid and kaempferol serve as the active compounds in PSO, which may activate nuclear receptors like PI3K, TNF, and IL-1β while suppressing inflammatory factors, thereby initiating lipid catabolism upstream and inhibiting inflammatory signaling. Moreover, ALA has been shown to mitigate endothelial inflammation under high-glucose conditions via the PI3K/Akt pathway [59,60]. The KEGG pathway enrichment analysis revealed the significant enrichment of these common targets in pathways including the “Lipid and atherosclerosis,” “PI3K-Akt signaling pathway,” and “AGE-RAGE signaling pathway.” Molecular docking was subsequently performed to validate the interactions between key chemical components of PSO and core target proteins. The results demonstrated strong binding affinities, with binding energies of less than −5 kcal/mol between key PSO constituents and critical targets. In particular, NOS3 exhibited the lowest binding energy among nine potential active components, suggesting its key role as a potential key target for PSO’s lipid-lowering activity.

The PI3K/Akt pathway is activated by cytokine and chemokine receptors in immune cells; it also recruits inflammatory cells and plays a significant role in the initiation of adipogenic transformation and adipocyte hyperplasia [61]. The PI3K/Akt regulates adipocyte growth and proliferation, which serves as a crucial modulator of hypercholesterolaemia-induced vascular disease and inflammatory bowel disease [26]. NOS3 is named for endothelial NO synthase (eNOS), a gene located downstream of the PI3K-AKT signaling pathway, which is also a key gene correlated with the levels of L-arginine metabolites [62]. Akt, upon activation by PI3K, directly phosphorylates NOS3, enhancing its activity and promoting nitric oxide (NO) release from endothelial cells, thereby exerting anti-inflammatory and anti-atherosclerotic effects [63]. Meanwhile, NOS3 gene polymorphisms also affect the lipid profile in cardiometabolic disorders [64]. To validate the network predictions at the protein level, we performed Western blot analysis. The results showed that PSO administration significantly increased the p-PI3K/PI3K and p-Akt/Akt ratios and upregulated NOS3 protein expression in the livers of the hyperlipidemic rats, further confirming the central role of the PI3K/Akt/NOS3 axis in mediating the therapeutic effects of PSO. Similarly, the main active ingredient in PSO, ALA, has been shown to attenuate hepatic steatosis in the HFD-induced rats via endoplasmic reticulum stress-mediated autophagy and the PI3K/Akt pathway [59]. Meanwhile, safflower seed, flaxseed, and soybean oils, serving as other plant oils, can also exert the lipid-lowering effects via the PI3K/Akt pathway [65,66].

## 5. Conclusions

In conclusion, 591 compounds were confirmed in PSO, including 152 fatty acids, 150 shikimates and phenylpropanoids, 145 terpenoids and others. Notably, ALA is the predominant PUFA, accounting for approximately 57.5%. PSO ameliorated lipid profiles, body weight, reduced hepatic steatosis/pathological damage through the in vivo experiment, and is probably associated with the potential mechanism activating the PI3K/Akt/NOS3 signaling pathway. Furthermore, PSO regulated fatty acid metabolism (e.g., ALA and arachidonic acid) and endogenous inflammatory responses against HFD-induced hyperlipidemia in rats. The novel findings of this study, obtained through the integrated multi-technique approach of serum metabolomics, network pharmacology, and molecular docking, provide scientific justifications for using PSO as a functional food for dietary intervention strategies in hyperlipidemia management. Future studies are warranted to elucidate the clinical potential for hyperlipidemia and metabolic cardiovascular diseases, and to achieve a more comprehensive understanding of the underlying mechanisms of PSO as a nutritional approach in treating hyperlipidemia.

## Figures and Tables

**Table 1:** Determination of the major fatty acid methyl ester components in PSO.

Number	Identification	Molecular Formula	Fragment Ions	Content (g/kg)	Retention Time/min	Relative Content of Fatty Acid Methyl Esters (%)
1	Methyl myristoleate	C_15_H_28_O_2_	74, 87, 55	0.154	24.01	0.05
2	Methyl pentadecanoate	C_16_H_32_O_2_	74, 87, 213	0.188	25.45	0.06
3	Methyl palmitate	C_17_H_34_O_2_	74, 87, 227	10.808	26.91	3.52
4	Methyl heptadecanoate	C_18_H_36_O_2_	74, 87, 241	0.374	28.46	0.12
5	Methyl stearate	C_19_H_38_O_2_	74, 87, 255	6.317	30.15	2.06
6	Methyl oleate	C_19_H_36_O_2_	264, 97, 83	67.540	31.35	21.98
7	Methyl linoleate	C_19_H_34_O_2_	81, 67, 95	42.611	33.27	13.87
8	Methyl α-linolenate	C_19_H_32_O_2_	79, 93, 67	176.653	35.73	57.48
9	Methyl arachidonate	C_21_H_42_O_2_	87, 74, 283	0.912	34.00	0.30
10	Methyl gadoleate	C_21_H_40_O_2_	292, 97, 83	0.991	35.43	0.32
11	Methyl docosanoate	C_23_H_46_O_2_	87, 74, 143	0.762	38.70	0.25

## Data Availability

Data are contained within the article. Further information can be obtained by contacting the corresponding authors.

## Supplementary Materials

The following supporting information can be downloaded at: https://www.mdpi.com/article/10.3390/foods14234125/s1, Figure S1: Methyl Fatty Acid GC-MS/MS Spectrum of Perilla Seed Oil; Figure S2: Network of compound-target-pathway; Figure S3: Western blot analysis of the expression of PI3K/AKT/NOS3 signaling pathway-related proteins. Table S1: Sources of materials and reagents. Table S2: Chemical composition analysis results of perilla seed oil. Table S3: The table of biomarkers intersecting between “Perilla Seed Oil” and “hyperlipidemia”. Table S4: GC-MS/MS calibration data, linear equations, and R^2^ for fatty acid methyl esters. Table S5: PSO affects serum lipid levels in rats. Table S6: Identification results of differential serum metabolites in control vs. PSO groups. Supplementary Table S7: PSO affects the relative expression levels of key serum metabolites. Table S8: Expression levels of key liver proteins.

## Abbreviations

The following abbreviations are used in this manuscript:

PSO	Perilla seed oil
ALA	Alpha-linolenic acid
HFD	High-fat diet
TC	Total cholesterol
TG	Triglycerides
LDL-C	Low-density lipoprotein cholesterol
HDL-C	High-density lipoprotein cholesterol
NAFLD	Non-alcoholic fatty liver disease
TCM	Traditional Chinese Medicine
PUFA	Polyunsaturated fatty acid
PPI	Protein–protein interaction
GO	Gene ontology
KEGG	Kyoto Encyclopedia of Genes and Genomes
BP	Biological process
CC	Cellular component
AA	Arachidonic acid
SDS-PAGE	Sodium dodecyl sulfate-polyacrylamide gel electrophoresis
