# Supplementary material for: Perilla Seed Oil Alleviates High-Fat-Diet-Induced Hyperlipidemia by Regulating Fatty Acid Metabolism via the PI3K/Akt/NOS3 Pathway
Source: Foods. 2025 Dec 1;14(23):4125. doi: 10.3390/foods14234125 (PMC12692184; doi:10.3390/foods14234125)
Supplement: Supplementary file 1 [file foods-14-04125-s001.zip › foods-3999280-Supplementary material.pdf]

Supplementary materials

Supplementarey Table S1 Sources of materials and reagents.

Material/reagent name	Supplier	Address
High-fat diet(63.6% Basal feed,20% sucrose,15% lard, 1.2% cholesterol,0.2% sodium cholate)	Liaoning Changsheng Biotechnology Co., Ltd.	Beixi, China
4% paraformaldehyde	Solarbio & Technology Co., Ltd.	Beijing, China
TC reagent kit	Nanjing Jiancheng Biotechnology Co., Ltd.	Nanjing, China
TG reagent kit	Nanjing Jiancheng Biotechnology Co., Ltd.	Nanjing, China
HDL-C reagent kit	Nanjing Jiancheng Biotechnology Co., Ltd.	Nanjing, China
LDL-C reagent kit	Nanjing Jiancheng Biotechnology Co., Ltd.	Nanjing, China
Hematoxy	CNW Technologies	Shanghai,China
eosin	CNW Technologies	Shanghai,China
Oil Red O solution	Solarbio & Technology Co., Ltd.	Beijing, China
Trizol	Solarbio & Technology Co., Ltd.	Beijing, China
Methanol	CNW Technologies	Shanghai,China
Acetonitrile	CNW Technologies	Shanghai,China
Quant-iT PicoGreen dsDNA Assay Kit	Invitrogen	The United States
Agarose	Invitrogen	The United States
Marker	Takara	Japan
2-Propanol	CNW Technologies	Shanghai,China
Acetic acid	SIGMA-ALDRICH	Shanghai,China
Ammonium acetate	SIGMA-ALDRICH	Shanghai,China
YP-Ab-14915 Anti-PI3K	Upingbio	Hangzhou,China
bs-6417R	Bioss	Beijing, China
Anti-phospho-PI3K YP-mAb-14652 Anti-AKT	Upingbio	Hangzhou,China
bsm-56203R Anti-phospho-AKT	Beijing, China	Beijing, China
YP-Ab-17170 Anti-NOS3	Upingbio	Hangzhou,China

Supplementarey Table S2 Chemical composition analysis results of perilla seed oil.

Compound	mz	rt/min	FORMULA	type	MS2 ion fragment	Responsesignal
Guvacine	128.0702	0.02	C6H9NO2	POS	127.0387726;128.070343;128.0426331;43.01779175;82.06494141;109.0281601;99.04377747;81.03334808;100.0755005;110.0598145;71.04901886;114.9713593;83.04911804;112.1117401;69.0333786;111.0438538;57.03349304;110.031723;55.05416107;86.05978394;85.0284729;55.01771164;100.0472794;69.06977844;101.0596085;84.04419708;53.03845978;72.08071136;84.08052063;97.02824402;83.08535767;72.05222321;85.06456757;67.05404663;86.09609222	1884612.163
L-Azetidine-2-carboxylic acid	84.0442	0.08	C4H7NO2	POS	42.03372955;84.04415894;55.05410767;43.05413437;83.08534241;41.03848267;83.04885864;59.04902649;56.04925919;57.03332901;71.07268524;56.05725861	20367563.87
Phloroglucinol	127.0386	0.10	C6H6O3	POS	127.038681;109.0281296;43.01777267;99.04379272;81.03328705;71.04898071;82.06497192;126.054657;55.01771927;69.03330994;100.0753479;53.03847885;85.94519043;110.0597534;108.0442352;85.02841187;126.0908585	102968670.4
Pyrogallol	127.0386	0.10	C6H6O3	POS	127.038681;109.0281296;43.01777267;99.04379272;81.03328705;71.04898071;82.06497192;126.054657;55.01771927;69.03330994;100.0753479;53.03847885;85.94519043;110.0597534;108.0442352;85.02841187;126.0908585	102968670.4
4-(dimethylamino)butanoate	132.1015	0.18	C6H13NO2	POS	86.09622955;90.94745636;72.93695831;90.90305328;131.9612732;70.95773315;114.9472961;131.929718;113.9635391;132.1018066;108.9578934;43.0177803;88.96840668;87.09951782;108.9135056;113.9509506;87.05473328;87.04391479;97.02818298;69.06980896;69.96086884;74.02346039;44.04948807;105.0695419;69.03331757;73.94540405;85.08384705;130.9625702;130.9788361	32235149.99
Kojic acid	143.0335	0.21	C6H6O4	POS	143.0336456;125.0231094;69.06973267;98.05979919;57.06978226;142.0857391;118.9670105;119.018898;97.0282135;142.122406;139.017746;55.05414963;142.0345764;43.01781082;121.0076523;83.08532715;85.02815247;99.04379272;84.08071136;142.0495605;102.0546112;126.0545273;141.9589691;120.9645844;126.0265198;70.06502533;71.04893494;59.04904175;101.0083847;42.03377914	17343016.02
Pipecolic acid	130.0859	0.22	C6H11NO2	POS	130.0859375;84.04417419;84.08055878;130.04953;129.0545349;70.06501007;43.01780701;85.0282135;72.93694305;88.07544708;112.1114655;112.0755463;88.95237732;71.04904938;83.04891205;57.03341293;88.11180115;129.0179596;69.06975555;85.04750061;85.08407593;129.138443;111.0435944;72.08057404;74.09619904;129.0737915;101.0592804;129.0437622;103.0536804;70.95758057;90.94737244;113.0232315;57.06970596;69.03314972;102.0548248;95.04898834;59.04882431;113.0589905;86.09611511;58.06497955;56.04945755;67.05387115;41.03859711;71.06829071;88.90773773;86.00576019;89.90627289;111.984314;42.22185135;89.93930817;126.5372162;70.94172668	8024632.167
Adenine	136.0614	0.22	C5H5N5	POS	136.0616455;81.06970215;95.085289;91.054039;107.0853882;135.1162872;93.0697403;79.05403137;73.95600891;67.05422211;109.100914;109.0647125;135.0805664;43.01776886	8110595.421
Hygric acid	130.0859	0.22	C6H11NO2	POS	130.0859375;84.04417419;84.08055878;130.04953;129.0545349;70.06501007;43.01780701;85.0282135;72.93694305;88.07544708;112.1114655;112.0755463;88.95237732;71.04904938;83.04891205;57.03341293;88.11180115;129.0179596;69.06975555;85.04750061;85.08407593;129.138443;111.0435944;72.08057404;74.09619904;129.0737915;101.0592804;129.0437622;103.0536804;70.95758057;90.94737244;113.0232315;57.06970596;69.03314972;102.0548248;95.04898834;59.04882431;113.0589905;86.09611511;58.06497955;56.04945755;67.05387115;41.03859711;71.06829071;88.90773773;86.00576019;89.90627289;111.984314;42.22185135;89.93930817;126.5372162;70.94172668	8024632.167

Compound	mz	rt/min	FORMULA	type	MS2 ion fragment	Responsesignal
Nipecotic acid	130.0859	0.22	C6H11NO2	POS	130.0859375;84.04417419;84.08055878;130.04953;129.0545349;70.06501007;43.01780701;85.0282135;72.93694305;88.07544708;112.1114655;112.0755463;88.95237732;71.04904938;83.04891205;57.03341293;88.11180115;129.0179596;69.06975555;85.04750061;85.08407593;129.138443;111.0435944;72.08057404;74.09619904;129.0737915;101.0592804;129.0437622;103.0536804;70.95758057;90.94737244;113.0232315;57.06970596;69.03314972;102.0548248;95.04898834;59.04882431;113.0589905;86.09611511;58.06497955;56.04945755;67.05387115;41.03859711;71.06829071;88.90773773;86.00576019;89.90627289;111.984314;42.22185135;89.93930817;126.5372162;70.94172668	8024632.167
3-Butynoic acid	85.0282	0.27	C4H4O2	POS	42.03373718;85.02817535;84.04417419;84.08054352;43.05414963;71.07279205;57.03337097;41.03849792;43.0370903;59.04893494;56.04944992;56.05751419;57.06985855;67.05406189;57.05735779;55.05408859;44.04946518;68.02414703;69.03355408	23098894.47
1-Deoxymannojirimycin (hydrochloride)	146.0807	0.36	C6H13NO4	POS	99.04377747;146.0809479;127.0387192;145.0493927;43.01777267;100.0754089;71.04899597;100.0471802;72.08065033;81.03330994;85.02826691;128.0422516;128.0703278;112.1117706;82.06494904;129.1379242;57.03338623;118.0646744;55.01767349;72.05239105;111.0436478;53.0384903;83.04906464;86.05977631;44.02110672;69.0334549;129.0546265;110.0598526;84.0440979;123.9638977;82.03661346;119.0490646;101.0232086;84.08067322;87.04388428;72.93709564;55.0539856	6982655.754
Choline	104.1066	0.36	C5H14NO	POS	104.1066284;60.08062744	15930360.73
Sorbose	203.052	0.37	C6H12O6	POS	203.0522461;119.0185928;101.0080338;161.0291595;161.0653992;129.1382751;137.0292511;112.1118393;179.0396271;120.0192719;142.0342865;60.04429626;179.0749207;143.0182495;82.99749756;102.0092392;84.08061218;162.0668182;184.0447998;120.964592;143.0550842;180.04039;162.0302887;72.08039856;184.0800476;159.0499115;179.978363	13739290.63
Galactose	203.052	0.37	C6H12O6	POS	203.0522461;119.0185928;101.0080338;161.0291595;161.0653992;129.1382751;137.0292511;112.1118393;179.0396271;120.0192719;142.0342865;60.04429626;179.0749207;143.0182495;82.99749756;102.0092392;84.08061218;162.0668182;184.0447998;120.964592;143.0550842;180.04039;162.0302887;72.08039856;184.0800476;159.0499115;179.978363	13739290.63
Allose	203.052	0.37	C6H12O6	POS	203.0522461;119.0185928;101.0080338;161.0291595;161.0653992;129.1382751;137.0292511;112.1118393;179.0396271;120.0192719;142.0342865;60.04429626;179.0749207;143.0182495;82.99749756;102.0092392;84.08061218;162.0668182;184.0447998;120.964592;143.0550842;180.04039;162.0302887;72.08039856;184.0800476;159.0499115;179.978363	13739290.63
Stachydrine	144.1014	0.42	C7H13NO2	POS	114.9858246;144.1016235;79.02101898;99.04377747;62.01834869;123.9911346;137.4758606;105.9804459;127.0386658;134.9194794;126.493782;113.9882584;43.0177803;114.4875488;107.4741135;71.04903412;143.0337219;123.4926834;116.4795837;81.03316498;98.46869659;85.0281601;61.01058197;98.05958557;104.9827728;119.9323883;122.9935913;108.016037;46.99479294;136.4776764;106.9759979;115.9814072;135.4990845;129.0126495;119.0185242;116.9090881	7327806.318
3-Pyridinemethanol	110.0596	0.43	C6H7NO	POS	110.0596771;88.00393677;99.51186371;87.00370789;97.00914764;81.03327179;109.1008682;77.99846649;78.9986496;42.03369904;90.5066452;68.04928589;109.028244;67.05407715;89.50649261;98.51159668;82.06485748;78.01305389;108.0167084;81.06952667;108.5173645;109.064476;109.075531;43.01774597;83.04893494;53.03839493;99.01148224;82.03661346;55.05410004;87.50524902	7027434.111
3-Hydroxy-2-methylpyridin	110.0596	0.43	C6H7N	POS	110.0596771;88.00393677;99.51186371;87.0037	7027434.1

Compound	mz	rt/min	FORMULA	type	MS2 ion fragment	Responses ignal
e			O		0789;97.00914764;81.03327179;109.1008682;77.99846649;78.9986496;42.03369904;90.5066452;68.04928589;109.028244;67.05407715;89.50649261;98.51159668;82.06485748;78.01305389;108.0167084;81.06952667;108.5173645;109.064476;109.075531;43.01774597;83.04893494;53.03839493;99.01148224;82.03661346;55.05410004;87.50524902	11
2-Hydroxypyridine	96.044	0.43	C5H5NO	POS	96.04408264;86.00331879;95.08525848	26214510.93
Spermine	203.2223	0.43	C10H26N4	POS	61.01054001;129.1384125;112.1118393;158.9625397;202.95224;203.2226563;84.08056641;80.94869995;187.9284363;143.9390259;159.9642334;63.0063324;72.08067322;142.9489899;172.9414215;113.1153183;130.1421051;58.06501389;127.907547;179.9542847;154.9312592;160.9602203;124.9387054;115.1228714;62.01393509;125.9465103;60.04432678;190.9525757;119.0179977;188.9308472;179.9765472;101.0078888;144.9104614;132.1487274	9540557.891
Spermidine	146.1646	0.44	C7H19N3	POS	72.08059692;146.0808411;79.02098083;78.01317596;100.0753479;62.01832199;115.9847794;146.1650543;124.991127;99.04371643;114.9857101;112.1117477;129.1382904;100.047081;115.4867706;116.4851227;125.4904175;62.98978806;128.0703125;125.9890594;43.01771927;75.0915451;82.06476593;61.01056671;138.9747925;116.9839783;128.0419617;127.0385742;130.0495148;84.08048248;87.04369354;123.9638596;71.04894257;127.4927902;145.1055145;132.9581299;84.04440308;138.4747009;145.0493774;81.0331192;134.9193878;118.064743;58.06498718;123.9913025;127.9928589;104.9629593;106.9800644;136.9192963;81.01673889;86.05973053;137.9768829;83.04888153;122.9643173;124.491951;73.0841217;145.4889069;136.4993591;85.02791595;110.0596542;111.0437469;135.9486389;44.02120209;46.99497223;126.4940948;72.93700409;122.9399338;128.4918518;80.00868988;126.9952316;82.03664398;106.4812698;101.0789261;60.08081818;90.94739532;102.0907898;121.965744;80.02437592;105.9809723;113.1148682;107.4734344;119.050705;64.01436615;137.9174194;57.03343201;113.9631195;55.05388641;139.4749146;114.4870682;135.9998016;107.4813385;123.4927292;69.03318024;102.0541916;86.09616852;59.60874557;57.74437332;47.12155533;112.5214996	4448700.779
Tetraethylene glycol	195.122	0.76	C8H18O5	POS	89.05947113;194.1173401;45.03342438;135.043808;195.1208801;133.0856628;195.0888367;58.06497955;61.01068115;138.0662842;151.0963898;153.9790497;107.0703506;59.04908371;136.0473328;153.9595032;149.1329346;177.0554962;152.9517212;107.0856552;130.0855408;81.06980896;136.0747986;151.9706879;178.0860443;107.0490723;149.0949554;67.05439758;159.1172943;73.90859985;95.08603668;55.81771469	14266507.34
Maltol	127.0386	1.31	C6H6O3	POS	127.0386887;43.01776505;109.0281143;99.0437851;81.03330994;71.04901886;82.06491089;126.0547256;108.044075;55.01767731;53.03852081;126.0909576;69.0333786;100.0754089;110.0595627;57.03344345	47878305.31
5-Hydroxymethylfurfural	127.0386	1.31	C6H6O3	POS	127.0386887;43.01776505;109.0281143;99.0437851;81.03330994;71.04901886;82.06491089;126.0547256;108.044075;55.01767731;53.03852081;126.0909576;69.0333786;100.0754089;110.0595627;57.03344345	47878305.31
4-Chlorophenylalanine	200.0466	1.32	C9H10ClNO2	POS	154.0414734;200.0469208;155.0449219;118.0648422;119.0726395;141.0099792	297193282.3
2-Methylcyclopentane-1,3-dione	113.0593	1.46	C6H8O2	POS	113.0594482;113.0231323;113.0342941;72.93695068;95.04889679;90.94738007;112.0754242;67.05416107;71.04901123;95.08526611;85.06452942;112.111702;57.03340149;112.0499954;43.01773834;112.0389481;86.05960846;70.95774841;70.02837372;68.04932404;70.06496429;96.00769043;69.06973267;89.0014267;72.08063507;55.05408478;84.0807724;108.9575958;96.04402161;72.04459381;84.04442596;100.5097122;83.04	18000007.97

Compound	mz	rt/min	FORMULA	type	MS2 ion fragment	Responsesignal
					850769;44.04950714;81.03341675;88.96838379;69.03325653;83.08603668;109.3156433;106.4965744;96.05212402;93.59537506;45.70609665;105.1958008	
Pentaethylene glycol	239.1481	1.51	C10H22O6	POS	89.05942535;133.0856934;216.9792786;234.9901276;239.1485748;198.9672546;170.9734344;177.1117401;195.1224976;59.04908371;209.9833069;90.06258392;151.0960083;107.0699463;142.9784698;130.0864258;87.04334259	33280219.97
5-Hydroxyhexanoic acid	115.0749	1.67	C6H12O3	POS	70.06496429;69.06973267;115.0388031;87.04382324;74.09620667;97.06459045;59.04906082;114.091011;115.0750732;71.08524323;55.05411911;74.93733215;73.06458282;114.0548019;43.01776886;98.02350616;97.02833557;92.9477005;73.03946686;45.03332138;41.03845978;72.93688965;86.05986786;43.05418015;55.01769638;98.05975342;57.03342056;99.00740814;90.94738007;72.08066559;71.0490799;88.07552338;79.05392456;68.04961395;44.04925537	23833283.91
Caffeine	195.0857	1.92	C8H10N4O2	POS	194.1171265;135.0437317;195.0875702;58.06499863;136.04776;138.0658722;177.0545044;81.06959534;93.06997681;95.08534241;145.0290985;166.0859528;97.06434631;72.18625641;77.41680145;47.24541855;59.62154388;64.23640442;47.74726105;158.7731323;51.14210129;66.67565155;110.7891464	54632682.38
Hexaethylene glycol	283.1741	2.34	C12H26O7	POS	89.05942535;133.0856323;177.1116638;283.174408;90.06277466;87.04363251;195.1228027;239.1470947;59.04889679;134.0888519	117022482.8
Dethiobiotin	197.1278	2.41	C10H18N2O3	POS	197.1284027;155.9739075;132.9579163;70.06498718;111.984108;72.08062744;173.9844971;169.1329498;150.9686584;174.9685516;98.05998993;179.1049347;124.1117401;141.1385193;100.0755157;154.073288;70.95788574;192.980484;129.9950867;107.0854263;114.9478683;156.9776154;135.1166534;81.06958008;152.1076508;153.0113831;113.9636459;121.0997009;180.1003418;196.0953369;172.9524536;63.7009964;156.9608765;112.987114;96.35207367;53.36520004;45.7339859	6263071.862
Veratraldehyde	167.0697	2.49	C9H10O3	POS	149.0593262;121.0646057;93.06956482;120.0804749;167.0681763;166.0844269;139.0749359;144.9576111;166.1229248;125.0596085;107.0855408;111.0801163;95.04927826;95.08560181;107.0491486;131.0854645;79.05420685;123.0801239;97.06466675;162.9689331;43.0178299;67.05400085;91.05438232;150.0643768;142.9669037;57.0334816;108.0448837;122.0691299;103.0539017;83.0859375;122.0590439;140.070282;81.06951904;83.04875946;70.06516266;150.0914459;109.0646896;94.07344055;55.01813126;85.50390625;125.070015;60.85826111;51.55077362;115.9634018;122.0960541;74.94785309	3855215.605
Nonaethylene glycol	415.2523	2.56	C18H38O10	POS	89.05941772;133.0856171;177.1117249;87.04384613;90.06263733;134.0891418;73.06455231;131.0704498;59.04922867;221.1370392;415.2527161	105502146.2
Diaveridine	243.1218	2.62	C13H16N4O2	POS	123.0801315;151.0751648;95.08526611;81.06961823;243.1224823;105.0697784;187.0960693;141.090744;79.05397034;169.0854034;124.0838928;133.0649872;86.09655762;101.0231323;84.08090973;219.9771729;57.0334816;237.9910126;152.0778351;242.0973816;72.08084869;144.3716736;84.04400635;110.071434;70.06522369;166.1598816;129.055191;98.51081085;72.604599;109.0651703;64.13043976;201.0632629	3288484.057
3-isobutyl-2,3,6,7,8,8a-hexahydropyrrolo[1,2-a]pyrazine-1,4-dione	211.1434	2.80	C11H18N2O2	POS	211.1437683;70.06499481;86.09621429;183.1489563;98.05981445;179.0699768;114.0910339;168.0778809;138.1276398;167.070282;210.203949;155.1539307;127.0863876;123.0437851;194.1169739	56822512.66
Syringaldehyde	183.0646	2.81	C9H10O4	POS	123.043808;95.04891205;155.0700226;140.0465851;183.0647125;105.0444107;124.0470886;182.0590057;77.03847504;67.05400848;138.1273499;96.05250549;156.0738525;159.969101;181.0496674;55.01763535;137.0593719	12510090.46
5-Hydroxy-1-tetralone	163.0748	2.82	C10H10O2	POS	131.0489044;163.0748749;103.0539703;163.0385284;107.0489044;162.0902863;162.0542908;16	6527155.116

Compound	mz	rt/min	FORMULA	type	MS2 ion fragment	Responses ignal
					2.1271057;135.0799103;132.052124;79.05393219;120.0805359;135.0440979;115.0540924;117.0691605;136.0751953;105.0699615;121.0071259;135.1170044;93.0695343	
(R)-Sulforaphane	178.0349	2.85	C6H11NOS2	POS	114.03685;71.99003601;119.0521774;55.05408859;178.0345001;177.0655212;177.1041718;118.068222;115.0405731;133.1003418;89.04165649;160.0752716;177.0512695;98.0597229;131.0852203;108.0440674;105.0698547;107.0857697;161.0952148;177.1448364;150.0911407	515601.7108
Paeonol	149.0592	2.86	C9H10O3	POS	149.0587158;93.06957245;121.0644302;91.05394745;149.0229797;105.0443192;77.03838348;95.04883575;131.085083;148.1109161;148.0738831;79.05393982;107.0851746;94.07260132;92.05709076;108.043808;122.0675049;103.0538177;107.0486221;148.0970001;55.01768875;81.06964111;106.0643616;147.0805817;126.0552902;50.53297043;63.21555328;90.05277252;88.71274567;82.01917267;62.594944;77.70688629	1266519.103
Kaempferol-3-O-galactoside	449.1065	2.86	C21H20O11	POS	329.0651855;299.0545654;449.1078491;413.085968;431.0966492;353.0652771;383.0762024;395.0751038;339.086792;359.0762024;330.0675964;300.0592957;70.06499481;311.0533142;367.0791931;432.1031189;84.08029938;414.0901794;129.1022339;86.09585571;354.0687561;365.0653992;325.0701904;287.0540161;72.08084869;313.0701294;377.0661316;396.0765381;371.0748596;341.0653992;217.0492401;384.0808105;110.0707397;243.028717;120.0809097;340.0910034;327.0503845;314.0812378;326.0768433;323.045929;86.62741852;440.2341003;272.0648499;65.77138519;343.0820313;312.0623779;93.36962891;120.5618362;59.30729294;159.0014648;259.0590515;50.04484558	5919763.693
3,5-dihydroxy-2-(4-hydroxyphenyl)-7-[(2S,3R,4S,5S,6R)-3,4,5-trihydroxy-6-(hydroxymethyl)tetrahydropyran-2-yl]oxy-chromen-4-one	449.1065	2.86	C21H20O11	POS	329.0651855;299.0545654;449.1078491;413.085968;431.0966492;353.0652771;383.0762024;395.0751038;339.086792;359.0762024;330.0675964;300.0592957;70.06499481;311.0533142;367.0791931;432.1031189;84.08029938;414.0901794;129.1022339;86.09585571;354.0687561;365.0653992;325.0701904;287.0540161;72.08084869;313.0701294;377.0661316;396.0765381;371.0748596;341.0653992;217.0492401;384.0808105;110.0707397;243.028717;120.0809097;340.0910034;327.0503845;314.0812378;326.0768433;323.045929;86.62741852;440.2341003;272.0648499;65.77138519;343.0820313;312.0623779;93.36962891;120.5618362;59.30729294;159.0014648;259.0590515;50.04484558	5919763.693
Isoscopoletin	193.0488	2.90	C10H8O4	POS	193.0491638;135.0437622;178.0254517;133.1008453;133.0282288;192.102005;161.0592499;58.06496811;105.0695496;83.04911041;192.1749573;119.0853424;192.0658417;133.0644836;81.0696106;192.1390076;169.990387;175.110672;93.06936646;147.1167297;95.08530426;109.1009598;137.0587311;166.0853424;175.1477661;91.05397797;137.096283;176.0709991;146.9732819;165.0538788;107.0848465;123.1167831;131.0857086	3126230.622
3-Hydroxy-4-methoxyacetophenone	167.0697	2.91	C9H10O3	POS	140.1066895;122.0961533;43.01777267;167.0695343;112.0753479;94.06489563;120.0803909;107.0725555;149.0950928;93.06965637;166.084198;125.0595245;96.08053589;67.05406189;166.1214447;107.0853271;121.1008682;131.0852814;68.04923248;79.05393982;95.08511353;80.049263;149.0587921;123.0797043;121.0642853;144.9584961;108.0437927;121.0837326	3651541.771
Acetovanillone	167.0697	2.91	C9H10O3	POS	140.1066895;122.0961533;43.01777267;167.0695343;112.0753479;94.06489563;120.0803909;107.0725555;149.0950928;93.06965637;166.084198;125.0595245;96.08053589;67.05406189;166.1214447;107.0853271;121.1008682;131.0852814;68.04923248;79.05393982;95.08511353;80.049263;149.0587921;123.0797043;121.0642853;144.9584961;108.0437927;121.0837326	3651541.771
4-methoxy-6-[(E)-prop-1-enyl]pyran-2-one	167.0697	2.91	C9H10O3	POS	140.1066895;122.0961533;43.01777267;167.0695343;112.0753479;94.06489563;120.0803909;107.0725555;149.0950928;93.06965637;166.08419	3651541.771

Compound	mz	rt/min	FORMULA	type	MS2 ion fragment	Responses ignal
					8;125.0595245;96.08053589;67.05406189;166.1214447;107.0853271;121.1008682;131.0852814;68.04923248;79.05393982;95.08511353;80.049263;149.0587921;123.0797043;121.0642853;144.9584961;108.0437927;121.0837326	
p-Dimethylaminobenzaldehyde	150.0909	2.98	C9H11NO	POS	150.0913696;105.0695877;93.05695343;149.0710754;91.05389404;133.0646515;149.0944061;95.0488205;149.0229797;93.06884766;107.0731125;123.0801773;122.096199;106.0723953;106.0650253;108.0806046;95.08558655;107.0852509;131.0854492;94.06505585;121.0642853;121.101059;124.0752792;79.05416107;123.0915527;109.0279617;107.0490036;81.06968689	2957511.167
3,3-Dimethylcyclohexanone	109.1008	2.99	C8H14O	POS	109.1008224;67.05406189;87.00375366;81.06964111;77.99851227;98.5116806;89.50645447;55.05403519;43.05408478;107.0855026;42.03376389;85.00683594;86.00608063	7839943.204
2-(1-hydroxyethyl)-3H-quinazolin-4-one	173.0704	3.02	C10H10N2O2	POS	173.0705719;131.9297943;149.940033;172.130661;62.05994415;172.1144104;131.973877;172.0730896;172.1690674;132.9579926;90.9031601;145.100769;150.9687958;83.08551788;155.9736481;131.085434;90.94746399;111.9838715;172.0939484;87.04418182;128.0704041;158.1086578;131.117157;126.0907669;155.1065674	3289653.32
(6S,7aR)-6-hydroxy-4,4,7a-trimethyl-6,7-dihydro-5H-benzofuran-2-one	197.1166	3.02	C11H16O3	POS	179.1062927;197.1166534;135.1165619;107.0852585;133.1009216;161.0958099;197.0827637;155.9740143;93.06957245;132.957901;182.0568542;180.1100159;111.9842453;173.9847412;151.1113129;169.1331024;105.0694199;150.9686279;121.0646744;119.0851974;137.0957336;136.1202698;109.1008453;95.0490036;91.05392456;134.1038971;137.0596924;123.0439758;109.064621;162.0991211;81.06987762;123.1165924;70.95782471;108.0886917;79.05412292;113.0708313;95.08543396	45296493.32
5-butyl-4-methoxy-6-methyl-pyran-2-one	197.1166	3.02	C11H16O3	POS	179.1062927;197.1166534;135.1165619;107.0852585;133.1009216;161.0958099;197.0827637;155.9740143;93.06957245;132.957901;182.0568542;180.1100159;111.9842453;173.9847412;151.1113129;169.1331024;105.0694199;150.9686279;121.0646744;119.0851974;137.0957336;136.1202698;109.1008453;95.0490036;91.05392456;134.1038971;137.0596924;123.0439758;109.064621;162.0991211;81.06987762;123.1165924;70.95782471;108.0886917;79.05412292;113.0708313;95.08543396	45296493.32
Coniferaldehyde	179.0697	3.06	C10H10O3	POS	147.0436859;179.0697784;161.0592651;119.0487823;55.01767731;148.0470276;133.0644226;105.0694962;162.062912;91.05396271;120.0523071;146.0359344;163.1106567;123.0439072;162.0892944;134.0684204;95.04870605;121.1010742;137.059433;136.0519104;107.0846024;115.0537796;164.0461578;145.101059;106.0730515;125.0595245;93.0695343;103.0540695;151.0746002;109.0642853;112.0753555;56.02108383;151.1116486;163.0384521;81.06969452;135.1162415;118.0411606;139.1114502	39569663.26
(1R,2S,6S,9S,10R,11R,14S,15S,18S,20S,23R,24S)-6,10,23-trimethyl-20-[(2R,3R,4S,5S,6R)-3,4,5-trihydroxy-6-(hydroxymethyl)tetrahydropyran-2-yl]oxy-4-azahexacyclo[12.11.0.02,11.04,9.015,24.018,23]pentacosan-17-one	576.3935	3.07	C33H53NO7	POS	89.05944061;133.0856781;103.0750809;147.1011963;87.04380798;191.1275635;177.1117401;101.0593719;70.06483459;86.09627533;205.1434631;131.0702209;235.1534271;129.1018219;84.08049774;129.0908661;559.3688965;73.02820587;72.0806427;134.0894012;161.1170502;120.080986;249.1684723;117.0909958;145.0862579;130.0857697;293.1955261;85.06465912;73.06478119;90.06279755;110.070816;148.1048279;576.3981323;189.1115112;221.1369476;131.1061249;116.0703659;104.0789948	5219344.484
(Z)-3-(4-oxo-5-propan-2-ylidene-2-propylfuran-3-yl)prop-2-enoic acid	219.1009	3.12	C13H16O4	POS	219.1007843;189.0906219;174.0671997;201.0902557;157.1220093;159.0800934;131.0853119;157.0644989;175.0749054;141.0694427;129.0694275;186.067337;169.0638428;191.1056366;190.0935822;158.072876;158.1255646;170.0715027;145.1007385;202.092804;218.1703186;161.0951996;218.1209412;95.08507538;145.0643005;218.2124481;187.0740356;173.0950775;160.0509949;160.0834045;144.0562897;81.06954956;137.0592194;95.04840851;163.0739441;105.0699615;	13037928.79

Compound	mz	rt/min	FORMULA	type	MS2 ion fragment	Responsesignal
					119.0852509;171.0806885;218.1533051;192.1090393;72.08052063;91.0539093;117.0700073;135.0804443;93.06932831;173.0596924;133.1009064;188.0826874	
(2R)-8-ethyl-7-hydroxy-5-methoxy-2-methyl-chroman-4-one	219.1009	3.12	C13H16O4	POS	219.1007843;189.0906219;174.0671997;201.0902557;157.1220093;159.0800934;131.0853119;157.0644989;175.0749054;141.0694427;129.0694275;186.067337;169.0638428;191.1056366;190.0935822;158.072876;158.1255646;170.0715027;145.1007385;202.092804;218.1703186;161.0951996;218.1209412;95.08507538;145.0643005;218.2124481;187.0740356;173.0950775;160.0509949;160.0834045;144.0562897;81.06954956;137.0592194;95.04840851;163.0739441;105.0699615;119.0852509;171.0806885;218.1533051;192.1090393;72.08052063;91.0539093;117.0700073;135.0804443;93.06932831;173.0596924;133.1009064;188.0826874	13037928.79
Azaleatin	317.0672	3.12	C16H12O7	POS	261.0414429;243.0307007;284.0576172;317.0674133;299.0561218;225.02005;262.0438232;269.047699;187.0054779;70.06494141;287.0570679;84.0806427;198.0335236;303.0898438;173.9967957;72.08060455;279.0507507;260.0450439;168.9949036;129.102066;251.0357819;86.09583282;171.0101624;244.0331116;213.0209198;300.0593872;301.0697021;164.9839325	3704440.269
Bilastine	464.2894	3.17	C28H37N3O3	POS	216.1741638;213.1117706;464.2997131;57.06975174;234.184845;85.06456757;217.177536;160.1117249;231.1221619;76.03905487;111.0438156;129.054245;214.1150055;83.04892731;51.59000015;235.1876678;174.1270599	767270.4847
Tryptophol	162.0908	3.22	C10H11NO	POS	144.0805054;162.0908661;145.0835266;161.0953979;117.0696869;105.069458;119.0849762;145.1008911;120.0805435;107.0850983;93.06977844;121.1007919;161.1320343;106.0649796;161.058609;133.1015472;79.05389404;118.0645752;161.1085968;107.0490417;161.0723572;134.0596466;133.064743;135.1166229;91.05376434;130.0654449;135.0796814;95.04877472;121.0649719;145.0630798;42.0381279	7877697.341
Cue-lure	207.1009	3.25	C12H14O3	POS	109.0281067;161.0957336;189.090683;107.048851;133.1010284;91.05396271;171.0799866;105.0697403;207.098999;83.08528137;93.0696106;143.0852814;67.05410004;111.0801163;97.02811432;95.08518219;123.0436783;69.0697403;79.05399323;104.1067352;119.0849609;95.04902649;207.0617523;147.1161957;121.0647049;206.1546173;206.1191711;97.06460571;123.0804367;81.06977844;133.064743;145.0649567;206.1904297;117.0697479;147.0801544;81.03335571;179.1068115;55.01785278;128.0622101;71.04938507;149.0594635;147.0438538;151.111557;110.0315018;99.00756836;111.0439072;121.0280991;72.0806427;135.0917206;149.0956421;153.0702209;206.0803375;162.099411;99.04379272;129.0695343;164.1071167;145.1018982;137.0963593;165.1272583;163.11203;53.03856659;99.08047485;95.01272583;190.093338;123.1166229;85.06522369;135.0441589;119.0491104;131.0857544;115.0537109;55.05402756;106.4624405;120.0804214;85.02850342;172.0838013;120.3258133	3327955.832
1-Methyl-6-phenyl-1H-imidazo[4,5-b]pyridin-2-amine	225.1115	3.25	C13H12N4	POS	102.127388;109.028038;161.0955963;137.0682831;189.0902252;224.1241455;207.1382751;88.11170197;225.1091309;133.1009064;107.0490265;165.0543213;136.0617218;93.06975555;225.0657196;171.0808411;95.08540344;207.1017151;111.0803528;121.0649185;123.0805969;167.1064758;83.08561707;91.05431366;97.02817535;67.05440521;179.1067352;100.1120529;119.0851364;107.0852737;81.06960297;143.085556;71.04903412;79.05426788;69.06984711;58.06507874;148.0611725;208.1330261;95.04891968;123.0436096;105.0699387;97.06468964;111.0443954;103.1314011;83.04903412;125.0948639;166.0574799;190.0929871;180.1756439;86.06009674;119.0494843;151.0750275;85.06476593;81.0328064;149.0951538;85.0283432;162.1678162;94.07362366;144.5863953;99.08055878;58.87798691	7315340.552

Compound	mz	rt/min	FORMULA	type	MS2 ion fragment	Responsesignal
1-(3,7-dihydroxy-3-methyl-2-oxatricyclo[7.3.1.05,13]trideca-1(12),5(13),6,8,10-pentaen-6-yl)ethanone	259.0956	3.29	$C_{15}H_{14}O_4$	POS	137.0230408;241.0856018;212.0465698;213.0900574;259.0951538;185.0958862;184.0513306;213.0523529;139.1112518;70.06491089;185.0592194;242.0883026;201.1312256;69.06983185;84.08071899;167.0851288;258.1197815;138.0268097;121.1013107;195.0801086;143.085495;157.064209;187.0746765;173.0237579;203.1269379;109.0284348;105.0695801;107.0488052;129.0697937;147.04422;258.1541138;223.07724;142.077301;199.0749054;72.08050537;170.0726471;157.1010742;159.0802765;214.0960083;93.069664;91.05425262;226.061676;203.1053467;171.0807953;232.096344;231.1021118;109.0647736;81.03330994;81.06967163;149.0956879;55.05411911;84.04454041;97.10133362;198.0681152;171.0440674;131.0853729;153.1024017;168.0880585;110.071312;185.1144257;133.0648804;119.0856705;55.01769638;86.09638214;189.0905762;159.0436401;159.6408997;92.2173233;56.02497482;57.03336334	33191685.91
10-methoxy-2,2-dimethyl-pyrano[3,2-g]chromen-8-one	259.0956	3.29	$C_{15}H_{14}O_4$	POS	137.0230408;241.0856018;212.0465698;213.0900574;259.0951538;185.0958862;184.0513306;213.0523529;139.1112518;70.06491089;185.0592194;242.0883026;201.1312256;69.06983185;84.08071899;167.0851288;258.1197815;138.0268097;121.1013107;195.0801086;143.085495;157.064209;187.0746765;173.0237579;203.1269379;109.0284348;105.0695801;107.0488052;129.0697937;147.04422;258.1541138;223.07724;142.077301;199.0749054;72.08050537;170.0726471;157.1010742;159.0802765;214.0960083;93.069664;91.05425262;226.061676;203.1053467;171.0807953;232.096344;231.1021118;109.0647736;81.03330994;81.06967163;149.0956879;55.05411911;84.04454041;97.10133362;198.0681152;171.0440674;131.0853729;153.1024017;168.0880585;110.071312;185.1144257;133.0648804;119.0856705;55.01769638;86.09638214;189.0905762;159.0436401;159.6408997;92.2173233;56.02497482;57.03336334	33191685.91
Xanthoxyletin	259.0956	3.29	$C_{15}H_{14}O_4$	POS	137.0230408;241.0856018;212.0465698;213.0900574;259.0951538;185.0958862;184.0513306;213.0523529;139.1112518;70.06491089;185.0592194;242.0883026;201.1312256;69.06983185;84.08071899;167.0851288;258.1197815;138.0268097;121.1013107;195.0801086;143.085495;157.064209;187.0746765;173.0237579;203.1269379;109.0284348;105.0695801;107.0488052;129.0697937;147.04422;258.1541138;223.07724;142.077301;199.0749054;72.08050537;170.0726471;157.1010742;159.0802765;214.0960083;93.069664;91.05425262;226.061676;203.1053467;171.0807953;232.096344;231.1021118;109.0647736;81.03330994;81.06967163;149.0956879;55.05411911;84.04454041;97.10133362;198.0681152;171.0440674;131.0853729;153.1024017;168.0880585;110.071312;185.1144257;133.0648804;119.0856705;55.01769638;86.09638214;189.0905762;159.0436401;159.6408997;92.2173233;56.02497482;57.03336334	33191685.91
Betaxolol	308.2211	3.31	$C_{18}H_{29}NO_3$	POS	308.2214355;290.2109985;291.2144775;262.2157898	95942380.32
2,6-Dimethyl-4-hydroxybenzaldehyde	151.0749	3.33	$C_9H_{10}O_2$	POS	151.0749969;107.0488892;108.0568924;136.0516663;91.05396271;135.0437469;150.0908813	44511352.79
(3R,4R)-4-[(4-hydroxy-3,5-dimethoxy-phenyl)methyl]-3-[(4-hydroxy-3-methoxy-phenyl)methyl]tetrahydrofuran-2-one	389.1582	3.33	$C_{21}H_{24}O_7$	POS	137.0595093;151.0751495;219.1009369;203.1061401;138.0627594;189.0905457;247.096817;325.1432495;188.0827637;343.1549683;371.1489563;205.0857239;177.0914459;294.1248169;172.08815;201.0912018;149.0593567;152.0777283;353.1374817;293.1164856;191.1070404;165.0549469;341.1366882;323.1283875;179.06987;162.0680542;326.1482544;335.1265564;220.1053772;313.1462097;175.0761414;299.1286316	632956726.2
6-[(2R)-2,3-dihydroxy-3-methyl-butyl]-7-hydroxy-chromen-2-one	247.0956	3.33	$C_{14}H_{16}O_5$	POS	188.0827332;203.1062164;189.0902863;172.0878754;247.0959167;201.0905457;173.0593719;174.0669403;191.1061249;229.0853424;204.1096344;190.0941162;158.0721893;175.0745544;143.0853424;173.091095;159.0799713;160.0880432;	72294288.53

Compound	mz	rt/min	FORMULA	type	MS2 ion fragment	Responsesignal
1-(4-methoxyphenyl)ethanone	151.0749	3.33	C9H10O2	POS	128.0616608;161.0592499;145.0644836;157.0645599;171.0802307;129.0693512;138.0670013;219.1008148;137.0594025;202.0959473;187.0749359;121.0641785;215.0700531;156.0569;216.0765839;214.0619812;198.0671082;186.0669861151.0749969;107.0488892;108.0568924;136.0516663;91.05396271;135.0437469;150.0908813	44511352.79
18-hydroxy-15-(3-hydroxy-3-methyl-butyl)-2-methoxy-7,9,13-trioxapentacyclo[10.8.0.03,10.04,8.014,19]icosa-1,3(10),5,11,14,16,18-heptaen-20-one	411.1401	3.33	C23H22O7	POS	411.140686;159.041748	85932201.7
4'-Hydroxyacetophenone	137.0596	3.34	C8H8O2	POS	137.0594482;122.0359344;136.061676;81.06967163;91.0539856;95.08526611;94.04103088;136.0508423;119.0851059	21288333.97
Homoveratric acid	179.0697	3.35	C10H12O4	POS	179.0697327;111.0437469;151.0750427;178.158493;133.0644531;161.0593567;178.1214752;105.0695801;178.0867004;83.04883575;137.0592957;109.0644455;133.1005859;95.01222992;107.0853195;81.03305054;69.03336334;95.04872894;123.0801163;79.05400848;91.05384827;110.0597;119.0851898;162.0912781;43.01782608;95.08538818;135.080368;93.06967163;132.077713;112.0470581;81.06965637;152.0785522;123.0439835;147.044281;119.0486984;150.0907593;124.0753937;162.062149;55.01776886	4872086.357
Xanthoxylin	179.0697	3.35	C10H12O4	POS	179.0697327;111.0437469;151.0750427;178.158493;133.0644531;161.0593567;178.1214752;105.0695801;178.0867004;83.04883575;137.0592957;109.0644455;133.1005859;95.01222992;107.0853195;81.03305054;69.03336334;95.04872894;123.0801163;79.05400848;91.05384827;110.0597;119.0851898;162.0912781;43.01782608;95.08538818;135.080368;93.06967163;132.077713;112.0470581;81.06965637;152.0785522;123.0439835;147.044281;119.0486984;150.0907593;124.0753937;162.062149;55.01776886	4872086.357
2,3,4,9-tetrahydropyrido[3,4-b]indol-1-one	187.086	3.37	C11H10N2O	POS	187.0865173;118.0649033;173.0593567;91.05399323;145.0643921;186.0898132;62.06003952;159.0773926;159.0924683;145.0979309;144.0809784	10789461.54
Fenspiride (hydrochloride)	261.1589	3.40	C15H20N2O2	POS	120.0805283;261.1595459;86.09619904;188.1431274;233.1647491;216.1375732;228.1100769;114.0910263;121.0841675;200.1148376;213.0866089;260.1347046;186.0996552;91.05400085;105.0695267;158.1039429;189.1465607;229.1126404;234.1681671;72.04404449;157.0991821;217.1416016;132.0802155;81.06958008;87.09967804;148.0754242;93.06932831;185.0952148;113.0347977;172.1198883;84.08031464;69.06955719;144.1022339	9702481.822
3-(4-Methoxy-3-methyl-6-oxopyran-2-yl)propanoic acid	195.0647	3.43	C10H12O5	POS	194.1170349;195.0644989;95.01251984;135.0437927;149.0594177;121.064537;177.0542145;93.06962585;58.06496811;81.0699234;153.0543518;178.1213684;136.0477905;139.0744171;177.1273956;107.0854721;107.0488892;167.0696869;91.05387115;138.0545349;125.0706787;167.0333405;70.06490326;79.05407715;57.03326035;105.0695496;67.05419922;136.0744476;97.06433105;71.04886627;111.0800934;150.0622711;125.0596008;131.0856628;138.0661621;133.1006317;153.1029205;153.0890961;69.06967163;98.05982208;81.03347015;99.04412842;152.0939636;148.1217499;159.1160431;55.01760101;131.0490112;123.0799484;87.04393005;103.0544357;99.81354523;109.1007767;109.0645447;89.36898804;80.36385345;83.04821014;148.075531;61.4251976;137.9597778;72.08085632	498269.457
(6-hydroxy-7-methyl-8-oxo-3-propyl-5,6-dihydro-1H-isochromen-7-yl) 2-hydroxy-4-methoxy-6-methyl-benzoate	403.1739	3.44	C22H26O7	POS	137.0594025;267.1222839;207.1011505;181.0855865;335.1272278;176.08284;353.1374207;179.0698395;138.0627289;151.0749969;325.1427002;336.1337585;177.0541687;385.1635437;175.0749207;249.111618;367.1531372;165.0540771;208.1043549;268.1254883;403.1758728;293.1170044;192.0777893;221.1170654;166.062027;182.0906677;177.0891113;189.0901642;321.1104736;191.0699463	71742322.67

Compound	mz	rt/min	FORMULA	type	MS2 ion fragment	Responses ignal
Camptothecin	349.1172	3.46	$C_{20}H_{16}N_2O_4$	POS	349.1176453;321.1227112;151.0388184;331.1070862;306.108551;293.1284485;322.1254272;304.0948181;266.1166687;294.1119995;331.1895752;292.0961304;276.1013794;170.0826111;72.080513;197.0708466;348.1106567;348.180542;84.08040619;119.0858459;120.0802841;147.3259125;93.06984711;133.1012726	3247509.35
Tetrahydrocurcumin	373.1633	3.47	$C_{21}H_{24}O_6$	POS	137.0594482;177.0906067;151.0750732;305.1166382;237.1117859;138.0627289;337.1427612;219.1013489;355.1538391;165.0541382;149.0594177;177.055603;295.1324768;121.064682;373.1626587;323.127533;306.1219177;217.0856934	379418471
Deguelin	395.1452	3.47	$C_{23}H_{22}O_6$	POS	395.145813;177.0539093;394.1365662;159.0413666;84.08058929;70.06495667;72.0806427;86.09617615;120.080368;89.05921936	11478931.48
Scutellarein	287.0542	3.48	$C_{15}H_{10}O_6$	POS	287.0545044;175.0749054;269.1528931	4039734.474
Demethoxycapillarisin	287.0542	3.48	$C_{15}H_{10}O_6$	POS	287.0545044;175.0749054;269.1528931	4039734.474
Valpromide	144.1378	3.50	$C_8H_{17}NO$	POS	144.13797;144.1016846;99.04377747;127.0386658;102.0910416;98.05978394;43.01778793;71.04896545;88.07540131;85.02820587;121.9658279;103.0863876;81.03338623;85.10099792;143.033371;57.0697403;58.02872849;120.9650421;43.05415726;84.08042145;143.0856171;126.1272354;116.1068268	5155022.817
Linaprazan	349.1975	3.50	$C_{21}H_{26}N_4O_2$	POS	349.1976929;289.1795654;332.223938;233.1533813;83.04894257;348.186676;290.1823425;107.0851364;119.0855026;331.1887512;145.10112;275.1995239;105.0695724;159.1163483;261.1848755;271.1700745;95.08547974;133.1013336;57.03339767;81.06965637;307.1912537;70.06502533;93.06962585;147.0810089;91.0539093;131.0852661;171.1165924;121.1010284;147.1156921;79.05410004;84.08059692;125.0955887;161.095047;109.0645523;257.1904907;69.03328705;86.09642029;173.0940857;67.05389404;83.08533478;173.1315002;305.1691284;135.0789948;205.1597748;204.100174;149.0961304;150.4784851;157.100647;123.8012238;160.2427979	3377107.085
Diethyltoluamide	192.1377	3.51	$C_{12}H_{17}NO$	POS	105.0696182;122.0961685;192.1379242;192.1024933;71.04895782;191.1178436;147.1166229;106.0730667;133.1010437;123.1000366;191.1432953;175.1114044;91.05406952;131.085144;107.0852661;93.06970215;119.0857697;173.1320801;146.095993;88.07552338;79.05415344;148.0742645;81.06967163;135.0801849;173.0955811;95.08511353;145.1010742;147.0801544;81.03322601;121.1010818;109.0646896;95.04911804	2136578.093
SCH442416	372.1514	3.51	$C_{20}H_{19}N_7O_2$	POS	137.0593719;151.0749817;203.1062927;177.0902557;189.0900574;247.0960236;188.0827789;219.1010742;305.1167297;176.0827026;152.0784607;149.0594025;172.0879364;237.1116333;325.1431274;204.1095734;248.099472;321.1112061;353.1372681;201.0903931;177.055069;322.1173096;165.0542297;371.1490173;293.1160889;337.1430359;294.1231995;355.1526489;190.0941315;295.1312561;335.1278076;138.0627441;191.1066895;326.1464844;175.0751648;121.0644989;220.1044922;179.0700836;323.1262512;136.0515442;343.1526489;354.1409302;174.0668488;161.0591888;372.1512756;229.0848694;262.0971375;261.0904236;336.1311646;173.0913239;173.0595093;307.1329956;217.0853577;277.1217041	31282751.24
N-(1-benzyl-2-hydroxy-ethyl)benzamide	256.1322	3.52	$C_{16}H_{17}NO_2$	POS	105.0331955;117.0695877;91.05397797;134.0961609;256.1332092;238.122345;256.0980225;106.0366592;122.059761;239.1022491;194.0093842;118.0728149;92.0573349;116.1432495;95.08521271;81.06970215;255.15271;229.1333008;72.08067322;135.0997162	13530885.4
5,7-dihydroxy-2-(4-hydroxyphenyl)-3,6-dimethoxy-chromen-4-one	331.0802	3.52	$C_{17}H_{14}O_7$	POS	331.0808105;315.0494995;316.0567017;270.0523376;330.1833191;287.0536804;195.0281372	5290320.877
5,7-dihydroxy-2-(2-hydroxyphenyl)-6,8-dimethoxy-chromen-4-one	331.0802	3.52	$C_{17}H_{14}O_7$	POS	331.0808105;315.0494995;316.0567017;270.0523376;330.1833191;287.0536804;195.0281372	5290320.877
3,7-Di-O-methylquercetin	331.0802	3.52	$C_{17}H_{14}$	POS	331.0808105;315.0494995;316.0567017;270.052	5290320.8

Compound	mz	rt/min	FORMULA	type	MS2 ion fragment	Responsesignal
			O7		3376;330.1833191;287.0536804;195.0281372	77
3,5-dihydroxy-2-(3-hydroxy-4-methoxy-phenyl)-7-methoxy-chromen-4-one	331.0802	3.52	C17H14O7	POS	331.0808105;315.0494995;316.0567017;270.0523376;330.1833191;287.0536804;195.0281372	5290320.877
3,5,7-trihydroxy-8-methoxy-2-(4-methoxyphenyl)chromen-4-one	331.0802	3.52	C17H14O7	POS	331.0808105;315.0494995;316.0567017;270.0523376;330.1833191;287.0536804;195.0281372	5290320.877
Andrographolide	351.2136	3.54	C20H30O5	POS	351.2134094;332.2219238;333.2017212;350.2404785;273.1843567;291.1958618;133.1008301;93.069664;107.0852509;102.0912018;145.1004944;81.06957245;119.085434;289.178009;147.0438538;161.0955505;84.08053589;67.05382538;70.06491089;121.0645981;149.0956421;121.1001129;55.05396652;168.1018372;255.1722717;159.1164856;314.2080688;86.0958786;290.1809387;72.08069611	2160582.36
(3E,4R)-3-[2-[(1R,4aS,5R,6R,8aS)-6-hydroxy-5-(hydroxymethyl)-5,8a-dimethyl-2-methylene-decalin-1-yl]ethylidene]-4-hydroxy-tetrahydrofuran-2-one	351.2136	3.54	C20H30O5	POS	351.2134094;332.2219238;333.2017212;350.2404785;273.1843567;291.1958618;133.1008301;93.069664;107.0852509;102.0912018;145.1004944;81.06957245;119.085434;289.178009;147.0438538;161.0955505;84.08053589;67.05382538;70.06491089;121.0645981;149.0956421;121.1001129;55.05396652;168.1018372;255.1722717;159.1164856;314.2080688;86.0958786;290.1809387;72.08069611	2160582.36
Pratensein	301.0696	3.59	C16H12O6	POS	301.0700378;286.0468445;197.0442352;287.0503235	16407845.95
1,3-dihydroxy-6-(hydroxymethyl)-8-methoxy-anthracene-9,10-dione	301.0696	3.59	C16H12O6	POS	301.0700378;286.0468445;197.0442352;287.0503235	16407845.95
5,6,7-trihydroxy-2-(4-methoxyphenyl)chromen-4-one	301.0696	3.59	C16H12O6	POS	301.0700378;286.0468445;197.0442352;287.0503235	16407845.95
Kaempferol 3-methyl ether	301.0696	3.59	C16H12O6	POS	301.0700378;286.0468445;197.0442352;287.0503235	16407845.95
(1S,19R)-8,10-dioxa-4,17-diazaheptacyclo[15.4.3.01,18.04,19.05,13.07,11.014,19]tetracosa-5,7(11),12,22-tetraen-3-one	337.1532	3.60	C20H20N2O3	POS	177.0541992;145.0281372;178.0575714;144.080368;337.1593323;117.0329742;146.031662	19796733.39
6''-O-Acetylglycitin	489.1376	3.61	C24H24O11	POS	285.0751343;286.0784607;125.0957031;189.1116486;97.10088348;227.1274719;245.1374664;171.1012268;489.1385498;83.08537292	7543871.935
[(8R,9S,10R,13S,14S,17S)-10,13-dimethyl-3-oxo-1,2,6,7,8,9,11,12,14,15,16,17-dodecahydrocyclopenta[a]phenanthren-17-yl] acetate	331.2257	3.62	C21H30O3	POS	331.2264709;97.06446075;331.0808105;109.0644226;315.0496521;123.0802994;269.1895447;161.1325226;313.2160645;316.0562744;295.2064209;83.04895782;127.0750961;81.06970215;251.178421;133.1010284;270.0522156;110.0679398;95.08509064;159.1164703;203.1422882;147.1166229;231.1736145;107.0852051;175.1116943;98.06806183;211.1474152;185.1321259;145.1006927;135.116394;119.0850983;213.1635132;171.1164093;121.0646362;131.085144;157.1011658;93.07009125;173.131897;313.0330811;314.2200012;71.04883575;84.08049011;215.1422577;149.0963135;287.0539246;69.0697937;55.05418777;199.1483002;137.0592041;187.1470947;215.1778259;229.1576996;125.0955353;255.1725464;105.0693588;120.0804596;195.1170044;93.07649994;117.0704651;85.06441498;202.1067963;271.2058105;225.1628876;153.088974;175.1472168	36166175.12
17alpha-Hydroxyprogesterone	331.2257	3.62	C21H30O3	POS	331.2264709;97.06446075;331.0808105;109.0644226;315.0496521;123.0802994;269.1895447;161.1325226;313.2160645;316.0562744;295.2064209;83.04895782;127.0750961;81.06970215;251.178421;133.1010284;270.0522156;110.0679398;95.08509064;159.1164703;203.1422882;147.1166229;231.1736145;107.0852051;175.1116943;98.06806183;211.1474152;185.1321259;145.1006927;135.116394;119.0850983;213.1635132;171.1164093;121.0646362;131.085144;157.1011658;93.07009125;173.131897;313.0330811;314.2200012;71.04883575;84.08049011;215.1422577;149.0963135;287.0539246;69.0697937;55.05418777;199.1483002;137.0592041;187.1470947;215.1778259;229.1576996;125.0955353;255.1725464;105.0693588;120.0804596;195.1170044;93.07649994;117.0704651;85.06441498;202.1067963;271.2058105;225.1628876;153.088974;175.1472168	36166175.12

Compound	mz	rt/min	FORMULA	type	MS2 ion fragment	Responses ignal
Propyl paraben	181.0853	3.63	C10H12O3	POS	139.038681;71.01256561;180.1372223;135.1165161;163.111084;93.03324127;121.1008682;140.0419769;111.0438538;180.10112;107.085228;93.0695343;81.06983185;145.101181;95.08547974;121.0283508;79.05406189;83.04902649;97.06481171;162.09198;113.0597534;119.085495;67.05420685;125.0596237;112.0758514;110.059433;181.0860901;154.0861206;69.03330231;181.069458;55.05414963;138.0910034;55.01787949;83.06015015;123.0809174;69.06984711;95.04910278;95.01275635;85.02830505;109.1009216;124.0754852	33129240.81
(±)-Dihydroactinidiolide	181.1217	3.65	C11H16O2	POS	181.1217041;135.1165466;139.0386963;163.1113739;107.0852661;121.1008682;95.08529663;139.0749359;145.1009521;93.06959534;71.01263428;81.06964874;125.0594254;180.1378326;123.0800858;93.03305817;109.1009445;136.1203156;119.0853043;153.1265717	34511401.04
Corticosterone	329.2099	3.68	C21H30O4	POS	329.2120667;231.1739502;97.06449127;213.1633148;109.0644684;123.080162;81.06958771;131.0853729;133.1010132;95.08535004;157.1006317;107.0853729;311.2002869;121.1007614;161.1321259;135.1165161;173.131897;232.1773682;171.1165009;83.04888916;121.0646667;102.1279984;141.0914001;71.04917908;105.0699005;99.04399109;123.116806;119.0856857;145.1014557;160.0758362;93.07007599;175.1125641;125.0961075;215.1435242;214.1672516;229.1588135;83.08531952;99.08010101;135.0802765;187.1481628;111.0801239;69.06998444;149.1320801;163.1111908;241.0855865;147.1166687;147.0807037;95.04891205;269.1908264;275.2013855;143.0854187;161.0965729;98.06801605;189.1629791;79.05418396;159.1171875;310.205719;137.0599823;120.0812683;189.1277618;163.0748291;113.0962143;70.06522369;149.0962982;139.1107941;55.05425262;85.06479645;139.0762482;129.0189056;203.1805878;195.1172485;110.0685501;185.1328278;155.1057587;298.0533752;175.0738068;168.1004181	4763720.067
Lauramine oxide	230.247	3.71	C14H31NO	POS	230.2474365;62.05991745;212.2368774;57.06986237;71.08546448;119.0851822;145.1008606;213.1269531;171.1168518;83.08509064;85.1007843;109.1008072;185.132431;213.1633301;72.0806427;117.0697327;229.2714539	5307879.43
Cytochalasin B	480.2732	3.82	C29H37NO5	POS	462.2628784;91.05399323;444.2523193;463.2666321;120.0803986;445.2562256;172.0753326;416.2579651;105.069603;119.0852585;145.1009827;480.2740173;107.0851822;252.1373138;240.1376801;159.1165619;93.06970978;426.2419434;133.1007538;264.1375122;161.0960846;95.08515167;111.0438614;250.1226349;81.06967163;417.2615356;146.0595551;427.2465515;143.085144;398.2478943;161.1317749;131.0852661;186.0907135;266.1539917;147.1165466;157.1009674;378.2044373;198.0908356;169.1007843;121.1009293;117.0697479;107.0488892;278.1531982;399.2498474;253.1395264;171.1164856;147.0804901;195.1162872;187.1137543;135.0802612;175.1126709;200.1066132;129.0695953;79.05413055;251.1268921;379.2084045;254.1531525;401.2459717;109.1011124;174.0905304;209.1326294;121.0839005;121.0649109;155.0852661;173.079071;92.0572052;256.1327209;183.1163788;241.1416779;268.1325073;402.250824;181.1015167;279.1754761;332.200592;434.2687378;207.1162567;141.0698547;173.132309;296.165802;197.1317596;265.1423035;297.1864319;149.0961456;173.0962982;238.1218872;235.1474457;136.0756226;280.1724243;270.1484375;212.1078186;435.2709656;236.1067352;221.1326599;282.1489258;251.1793976;184.0751953;193.1006622;242.1529999;106.0731125;267.1587524;83.04917145;67.05420685;123.080368;464.2714539;167.0858154;226.1230316;211.1481628;135.1168518;189.1273193;185.1323547;298.1849976;408.2310181;133.0648804;146.1044464;225.1632233;334.2169495;284.1645508;254.1169434;333.203369	617702.8279

Compound	mz	rt/min	FORMULA	type	MS2 ion fragment	Responsesignal
					1;223.1475525;276.1376038;268.169281;109.0648575;199.1472473;185.081192;280.1325989;290.153595;233.1321716;344.2015076;198.1306458;384.2350159;179.107254;262.1225891;51.36366272;318.1841431;292.1685791;160.1214294;269.1358032;310.1789551;320.2018127;151.0750732;179.0860443;236.1498718;409.2387085;163.1116791;446.2602234;308.1622009;362.2077942;343.1898499;219.1166229;352.2269897;324.1950073;187.0938873;137.0956116;360.1925659;158.1056671;224.1071625;86.09602356;171.0808258;237.1641388;160.075119;210.1351776;99.04394531;339.2128906;210.0921021;237.1130371;224.1458435;108.0888977;51.47501755;165.0908051;365.2304688;69.06959534;213.1271515;134.1052246;96.04424286;325.1794434;364.2257996;255.1575928;96.08853912;144.0803833;171.1004486;199.0940247;188.1083069;255.1252289;283.1538391;288.1616516;83.08565521;201.1103973;55.01785278;208.1216888;249.165451;345.2039185;338.2113647;281.1775818;247.1499634;261.1626892;307.1950073;350.2123413;263.1281433;125.059906;304.1697998;196.1208954;293.1758423;148.1217499;212.1461792;115.0541382;170.103653;346.2168884;214.1232147;363.2184753;132.0897675;263.178833;175.0953369;211.1127014;85.06439209;94.07302094;55.05440903;294.1476746;190.128479;291.1592102;97.06495667;205.1004639;234.1342163;336.1936951;239.1260529;112.0476074;271.1713562;418.2633362;342.1857605;361.1957703;149.1332092;228.1377563;299.2016907;358.2199707;162.134552;184.1212616;257.1362915;153.0708771;249.1162872;276.1744385;84.08087158;347.2215271;223.1103516;53.47658539;98.20852661;140.0673981;353.1986389;70.06526947;85.0282135;180.0908356;194.1056824;222.1351776;94.06571198;55.81617355	
Camphor	153.1268	3.93	C10H16O	POS	153.1270447;83.04895782;97.06450653;93.06952667;135.1167297;107.0851288;69.06967163;95.08531189;136.0394897;79.05380249;111.0799408;43.01781845;152.0702515;81.06969452;67.0541687;55.01779938;109.1009293;55.05410385;91.05413055;152.1053619;136.0752411;135.0808716;71.04917145;84.05210876;117.0696411;152.0563202;125.0956192;57.03319931;152.1424408;94.06536865;57.06949997;61.03491974;42.76568604;84.04416656;41.263134;124.0850067;88.97328186;134.5739288	5473295.775
5,7,11,19-tetraoxapentacyclo[10.8.0.02,10.04,8.013,18]icosa-2,4(8),9,13(18),14,16-hexaen-16-ol	285.0747	3.94	C16H12O5	POS	285.0751343;270.0518494	1635756248
3-hydroxy-2-(4-hydroxyphenyl)chromen-4-one	255.0642	3.97	C15H10O4	POS	255.0645599;194.0088196	2859582.849
Shogaol	277.1791	3.97	C17H24O3	POS	137.0594177;93.06961823;135.1165619;121.1008835;81.06967926;107.0852585;95.08527374;149.1321869;79.05404663;67.05410767;147.1165314;109.1007767;133.1009064;259.2052307;119.0852814;69.06973267;277.1333923;105.0695648;91.05396271;161.1321564;276.2019348;83.08532715;277.1816711;145.1006317;241.1947937;131.0853882;111.0438919;175.1112976;149.0959778;163.1476746;163.1113281;111.0801773;123.1166306;85.06449127;161.0959778;231.1738586;71.08538055;171.1165466;55.05414963;151.1112518;175.1476898;159.1161804;221.1535645;185.1323853;177.1269989;207.1378021;165.1269379;109.0645523;97.06456757;99.08011627;217.1944733;179.1064301;83.04881287;97.10087585;259.1697388;157.1003723;173.1321259;57.06978226;123.0798721;193.1218262;117.0697708;143.0853424;213.1632996;189.127182;179.1426697;227.1070709;136.1203461;55.01766968;203.1784668;165.0906982;138.0628967;181.1221466;71.04903412;235.1694794;195.1374512;125.095993;148.1198273;122.1042633;87.0438385;203.1430054;260.2035217;129.0697174;249.18	13113045.04

Compound	mz	rt/min	FORMULA	type	MS2 ion fragment	Responsesignal
					47382;125.0595169;187.1483002;231.210144;151.0750122;134.1045074;72.08068848;127.1112595;163.0756531;139.1114655;241.1591187;177.163559;82.07304382;167.1066284;120.088913;199.1481781;201.1636963;153.090683;150.1359558;57.03342056;96.0885849;94.07292938;113.0953903;193.1576691;85.10083008;185.0964966;155.1068115;258.1904907;108.088913;249.2193298;189.1634216;162.1359558;217.1590576;232.175705;106.0730515;263.0952148;127.0748138;199.1109924;164.114975;153.1273346;245.1169586;160.1197052;110.1045609;209.1512299;139.0756683;217.1225433;260.16922;176.1525726;146.1042023;101.0586777;132.0889893;174.1356354;141.1273651;191.1438599;164.1519775;80.05761719;100.0830536;215.179657;59.04907227;178.131897;236.1696625;204.1411743;124.1198883;230.1565094;180.1107635;246.0868988;250.2198944;51.67687607;197.1343994;95.80503082;68.05767822	
1,3-Dicyclohexylurea	225.1954	4.04	C13H24N2O	POS	225.1955719;100.1117706;102.1274261;143.1174927;83.08524323;88.11180115;61.03952026;81.06968689;207.1374817;95.04885101;101.1149292;95.08548737;97.06460571;55.05415344	41089926.61
Methyl 2-(3-oxo-2-(pent-2-en-1-yl) cyclopentyl)acetate	225.1478	4.07	C13H20O3	POS	225.1492767;100.1118088;102.1274643;143.1177063;83.08532715;207.1375885;61.03953171;88.11183167;95.04894257;168.0776062;81.06964874;95.08542633;189.1271515;93.06942749;171.1164093;147.116684;81.0331955;91.05392456;109.0644302;161.1324768;105.0694885;123.0806808;69.07002258;67.05387878;101.1150818;79.0539093;121.10112;133.1011505;55.05394745;97.06450653;107.0851135;119.0851288;85.10069275;149.0958557;109.1006851;97.10076904;121.0642853;165.1274261	3716709.382
8-[3-oxo-2-[(E)-pent-2-enyl]cyclopenten-1-yl]octanoic acid	275.1996	4.09	C18H28O3	POS	275.2000732;147.1165771;133.1009674;119.0853195;105.0696182;257.1896973;91.05404663;145.1008759;159.1165009;81.06971741;95.08534241;107.0852966;161.132431;131.0852051;93.06964111;149.0957642;83.08538055;173.1321106;185.1320801;239.1789856;67.05413055;121.10112;79.05406189;69.06978607;55.05414581;117.0696869;135.1165924;163.1114502;203.1424561;157.1006927;109.1008377;171.116394;183.1162262;229.1946411;148.1197357;143.085556;197.1322937;57.03339767;121.0642548;97.10051727;161.096344;107.0489044;85.06424713;175.1118622;175.1476898;135.0802155;97.06458282;219.1372986;177.1272125;169.1009216;109.0643845;134.1043549;219.1740723;129.0696564;201.1630554;111.0805435;71.04898071;211.1469269;123.0801392;187.1114807;201.1271973;258.1912842;215.1790314	53115001.92
(4aR,5S,8aS,9aR)-9a-hydroxy-3,4a,5-trimethyl-5,6,7,8,8a,9-hexahydro-4H-benzo[f]benzofuran-2-one	233.1527	4.12	C15H22O3	POS	233.1520538;105.0695724;119.0852585;131.0852356;91.05399323;232.168335;93.06959534;215.1424713;79.05406952;81.069664;173.132309;145.1011047;187.1477966;117.0696564;107.0853806;133.1008301;232.2041168;95.08535004;121.0645065;197.1324615;107.048645;67.05402374;69.06958771;171.1166687;135.0803986;121.1007462;159.1162262;205.1573486;133.0643921;109.0641174;232.1342621;55.05388641;155.0844574;83.08489227;147.0787811;169.0997162;129.0691223;159.0795441;123.0795059;122.0954361;109.1002808;135.11586;132.0877686;147.1155548;97.06394958;141.0687561;71.04869843;149.09552;106.0723572;161.1316833;120.0880508;95.04906464;216.1427002;111.0799179;83.048172;191.1423035;161.0953674;157.1001587;188.147934;163.1102753;123.1156235;57.06961823;137.0953369;177.0891266;103.0533752;124.2367935;73.06413269;143.0843811;99.07906342;72.04446411;57.0331955;80.69876099;55.01760864;51.85794449;51.87754059	4115881.825
2-[(1S,2S,4aR,8aS)-1-hydroxy-4a-methyl-8-methylene-decalin-2-yl]prop-2-enoic acid	233.1527	4.12	C15H22O3	POS	233.1520538;105.0695724;119.0852585;131.0852356;91.05399323;232.168335;93.06959534;215.1424713;79.05406952;81.069664;173.132309;145.1011047;187.1477966;117.0696564;107.0853	4115881.825

Compound	mz	rt/min	FORMULA	type	MS2 ion fragment	Responsesignal
					806;133.1008301;232.2041168;95.08535004;121.0645065;197.1324615;107.048645;67.05402374;69.06958771;171.1166687;135.0803986;121.1007462;159.1162262;205.1573486;133.0643921;109.0641174;232.1342621;55.05388641;155.0844574;83.08489227;147.0787811;169.0997162;129.0691223;159.0795441;123.0795059;122.0954361;109.1002808;135.11586;132.0877686;147.1155548;97.06394958;141.0687561;71.04869843;149.09552;106.0723572;161.1316833;120.0880508;95.04906464;216.1427002;111.0799179;83.048172;191.1423035;161.0953674;157.1001587;188.147934;163.1102753;123.1156235;57.06961823;137.0953369;177.0891266;103.0533752;124.2367935;73.06413269;143.0843811;99.07906342;72.04446411;57.0331955;80.69876099;55.01760864;51.85794449;51.87754059	
1-Deoxypebrolide	415.21	4.13	C24H30O6	POS	119.0851898;120.0884705;133.0644531	487844437.1
6a-acetyl-1-(carboxymethyl)-5,5-dimethyl-1,3a,4,6-tetrahydropentalene-2-carboxylic acid	281.1373	4.13	C15H20O5	POS	135.0802307;107.0852661;69.0333786;83.04898071;85.02818298;57.03340912;129.0544739;91.03877258;111.0438538;61.02830887;119.0853119;147.0649567;263.2001038;81.03337097;95.0852356;81.069664;136.083725;99.04376221;87.04376221;73.02817535;109.1011658;91.05379486;67.05400085;55.05416489;121.1011353;71.04909515;147.1166992;133.1012878;105.0696411;99.0803833;151.1116028;93.06965637;281.0806885;108.0885773;71.08543396;137.0960999;79.05415344;280.2002869;281.1318665;59.04894638;97.10095215;97.06460571;161.1322174;55.01770782;280.2342224;123.1168289;103.0386963;110.104599;96.08892822;82.07294464;280.2822571;264.1994629;223.1326904;115.0861511;165.1277008;125.0964432;109.064682;262.1837463;101.0596161;72.08062744;145.1013489	11265907.49
(1S,2S,12S)-3,11-dihydroxy-1,2,5-trimethyl-spiro[8-oxatricyclo[7.2.1.02,7]dodec-5-ene-12,2'-oxirane]-4-one	281.1373	4.13	C15H20O5	POS	135.0802307;107.0852661;69.0333786;83.04898071;85.02818298;57.03340912;129.0544739;91.03877258;111.0438538;61.02830887;119.0853119;147.0649567;263.2001038;81.03337097;95.0852356;81.069664;136.083725;99.04376221;87.04376221;73.02817535;109.1011658;91.05379486;67.05400085;55.05416489;121.1011353;71.04909515;147.1166992;133.1012878;105.0696411;99.0803833;151.1116028;93.06965637;281.0806885;108.0885773;71.08543396;137.0960999;79.05415344;280.2002869;281.1318665;59.04894638;97.10095215;97.06460571;161.1322174;55.01770782;280.2342224;123.1168289;103.0386963;110.104599;96.08892822;82.07294464;280.2822571;264.1994629;223.1326904;115.0861511;165.1277008;125.0964432;109.064682;262.1837463;101.0596161;72.08062744;145.1013489	11265907.49
6-[[3-(hydroxymethyl)-5-oxo-2H-furan-4-yl]methyl]-5,6-dimethylcyclohexene-1-carboxylic acid	281.1373	4.13	C15H20O5	POS	135.0802307;107.0852661;69.0333786;83.04898071;85.02818298;57.03340912;129.0544739;91.03877258;111.0438538;61.02830887;119.0853119;147.0649567;263.2001038;81.03337097;95.0852356;81.069664;136.083725;99.04376221;87.04376221;73.02817535;109.1011658;91.05379486;67.05400085;55.05416489;121.1011353;71.04909515;147.1166992;133.1012878;105.0696411;99.0803833;151.1116028;93.06965637;281.0806885;108.0885773;71.08543396;137.0960999;79.05415344;280.2002869;281.1318665;59.04894638;97.10095215;97.06460571;161.1322174;55.01770782;280.2342224;123.1168289;103.0386963;110.104599;96.08892822;82.07294464;280.2822571;264.1994629;223.1326904;115.0861511;165.1277008;125.0964432;109.064682;262.1837463;101.0596161;72.08062744;145.1013489	11265907.49
Eplerenone	415.21	4.13	C24H30O6	POS	119.0851898;120.0884705;133.0644531	487844437.1
Fenobucarb	208.1325	4.18	C12H17NO2	POS	96.04412079;104.1066818;93.06959534;207.1378784;208.1343536;81.0696106;147.1168518;208.1110992;107.0850067;95.08531952;79.05395508;149.0593719;67.0541153;161.1323395;91.05361938;127.1228333;171.1166534;97.04782867;207.1157074;189.1270142;105.0696259;163.1480	5641717.977

Compound	mz	rt/min	FORMULA	type	MS2 ion fragment	Responsesignal
					713;109.1009521;119.0854416;121.1006546;133.1008606;97.06458282;95.04893494;135.1163025;109.0648193;191.1423187;123.0806732;207.1748505;83.08532715;105.1106873;172.1200409;149.132782;69.06985474;131.0851135;111.0437164;129.0697174;71.08532715;55.05422211;145.1012726;163.1118164;71.04917145;173.1318512;148.1195374;149.0954742;121.0647202;94.07317352;98.05965424;162.1280975;137.0955811;192.0937653	
Progesterone	315.2307	4.22	C21H30O2	POS	315.2319641;109.0644989;97.06452179;297.2208862;123.0801926;279.2098999;253.1940918;271.2053528;83.04897308;173.1321564;95.08532715;147.1166382;201.1634064;177.1270294;159.1164246;81.06972504;163.1478424;145.1006165;107.08535;133.1007385;215.1787872;171.1165924;161.1320496;298.2240906;121.1009293;119.0852966;135.1167755;131.0850067;239.1793365;197.1318512;189.1626434;213.163147;93.06964111;187.1473389;105.069664;121.0645218;189.1289368;175.1476746;185.1320953;157.1008911;125.0956726;110.0676117;251.1786346;209.1321564;98.0677948;99.08026886;149.1322937;211.1476135;227.1438293;149.0959167;280.2131348;199.1479034;143.0853729;223.1487122;163.1114655;183.1156616;227.1773834;169.1008911;241.1587372;161.0958862;255.2098541;71.08537292;83.08519745;79.05414581;237.1637421;272.2091064;254.1980743;69.06973267;85.0644455;67.05399323;137.0958862;225.1633148;88.11180878;55.05398178;124.0836945;314.2829895;175.1113586;111.0803452;269.2260132;155.0849457;135.0798798;269.1896667;203.141983;257.1903687;187.1127319;255.1746674;117.0699615	31127446.76
5-Hydroxy-7,8-dimethoxyflavone,Moslosooflavone	299.0902	4.25	C17H14O5	POS	299.0906677;284.0673523;256.0726318	515429099.5
5-hydroxy-3,7-dimethoxy-2-phenyl-chromen-4-one	299.0902	4.25	C17H14O5	POS	299.0906677;284.0673523;256.0726318	515429099.5
10-(1,1-dimethylallyl)-5-methoxy-2,2-dimethyl-pyrano[3,2-g]chromen-8-one	327.1581	4.28	C20H22O4	POS	95.01248169;327.1576233;163.0750275;135.0801392;271.0960083;69.06973267;164.0828552;97.06451416;217.1220398;155.1064911;231.1005554;309.1468201;83.04891968;107.0851974;109.0645676;81.06968689;93.06963348;232.1459351;159.0796356;109.1007767;67.05407715;165.0903168;217.0856171;253.0862427;121.0645294;309.1890869;161.0591431;215.1427155;119.0856018;173.0960236;175.0747833;225.0901031;175.0386353;326.2278748;105.0697937;265.2161865;149.0591125;121.1013794;326.2821655;79.05409241;137.0953979;147.0437317;199.1111755;145.1009827;213.0549469;91.0541687;231.1368103;139.1111908;96.01611328;145.0647583;83.08505249;117.0693512;81.03327942;97.10092926;189.0904999;259.1317444;147.0802917;133.101059;125.0961304;243.1003265;123.1164169;55.05418015;131.0855103;272.0997009;147.116272;55.01773453;123.0801392;239.106308;161.0956573;267.0995178;113.0230713;136.0847778;172.1321259;281.1517639;177.090744;198.1269226;211.0741272;239.0690002;185.0587921;133.0642242;171.1161804;159.1165161;185.0950012;213.1255798;111.0800934;257.0812073;129.0695801;241.1219177;157.1010132;201.0905609;195.1163483;199.0738831;85.06443787;291.1384277;175.1116638;71.08528137;203.0696411;111.0259857;99.07992554;197.0952606;149.0957947;143.0855713;310.150116;191.0696564;285.1118774;201.0540314;71.04903412;139.0580597;229.0853271;197.1322632;57.06972885;157.063797;156.109314;151.074585;94.07301331;185.1318665;223.1112976;275.1990967	18671699.39
(1S,4aR,6aS,6bR,10R,11R,12aR,14bS)-1,10,11-trihydroxy-2,2,6a,6b,9,9,12a-heptamethyl-1,3,4,5,6,6a,7,8,8a,10,11,12,13,14b-tetradecahydropicene-4a-carboxylic acid	471.3455	4.33	C30H48O5	POS	205.1581726;201.1633911;147.1165161;187.147644;407.3305664;177.1630707;425.3413696;219.1735687;189.1634827;119.0853195;131.0852509;133.1008606;135.1166229;121.1008301;107.085083;95.085289;175.1477661;207.1740112;159.1164551;145.1009827;202.166626;109.1007156	1566538.367

Compound	mz	rt/min	FORMULA	type	MS2 ion fragment	Responsesignal
					;206.1617889;163.1111298;173.1323242;161.1322632;99.08024597;149.1321106;471.3474426;453.3362732;203.178894;353.2853088;191.1783752;163.1472015;81.06967163;215.1790466;213.1639099;105.070015;93.06994629;389.3208313;426.3447571;83.08552551;199.1482239;408.3330994;188.1515656;123.11689;88.11196136;217.1587524;148.1204987;227.1797638;102.1276093;247.1684875;435.3235474;185.1331787;151.1115417;215.1433563;245.1903229;125.0958557;233.189209;223.1683655;111.0805511;178.1674347;69.0700531;67.05413818;217.1947479;191.1430817;171.1165466;241.1942902;91.05413055;149.0967712;203.1438751;200.1531982;231.173584;118.1227646;97.10116577;157.1009216;57.06969452;211.1494904;208.177597;239.1789856;255.2100372;195.1390839;83.04947662;190.1655121;204.5315552;193.1586914;137.0962982;137.1311798;269.1900635	
7-hydroxy-3-(4-hydroxyphenyl)-5-methoxy-chromen-4-one	285.0747	4.34	C16H12O5	POS	285.0751343;270.0516968;284.2943115;284.3315125;242.0570984	49413406.29
1-phenylbutane-1,3-dione	163.0747	4.39	C10H10O2	POS	105.0331802;163.0750122;106.0365601;107.0850372;93.06965637;91.05399323;121.1008682;77.0381012	12791179.31
9-hydroxy-7-isopropyl-1,4a-dimethyl-2,3,4,9,10,10a-hexahydrophenanthrene-1-carboxylic acid	317.2091	4.41	C20H28O3	POS	317.2084045;316.2272339;300.2322693;95.08518982;119.0849075;81.06951141;121.1004715;107.0855179;316.1758118;93.06972504;145.1009369;133.1010284;316.3000793;131.0855713;105.0695877;159.1166382;147.1170349;109.1010742;299.1992493;67.05410004;69.06990051;149.1323242;160.0757446;299.2391052;173.1326752;187.1481628;271.204071;161.1322327;83.08529663;171.1179199;135.1168671;88.11214447;91.05407715;163.1113892;277.2158203;175.1482849;79.05395508;149.0947266;253.1961975	4126243.367
7-(1-hydroxy-1-methyl-ethyl)-1,4a-dimethyl-2,3,4,9,10,10a-hexahydrophenanthrene-1-carboxylic acid	317.2091	4.41	C20H28O3	POS	317.2084045;316.2272339;300.2322693;95.08518982;119.0849075;81.06951141;121.1004715;107.0855179;316.1758118;93.06972504;145.1009369;133.1010284;316.3000793;131.0855713;105.0695877;159.1166382;147.1170349;109.1010742;299.1992493;67.05410004;69.06990051;149.1323242;160.0757446;299.2391052;173.1326752;187.1481628;271.204071;161.1322327;83.08529663;171.1179199;135.1168671;88.11214447;91.05407715;163.1113892;277.2158203;175.1482849;79.05395508;149.0947266;253.1961975	4126243.367
(1S,4aS,9R)-9-hydroxy-7-isopropyl-1,4a-dimethyl-2,3,4,9,10,10a-hexahydrophenanthrene-1-carboxylic acid	317.2091	4.41	C20H28O3	POS	317.2084045;316.2272339;300.2322693;95.08518982;119.0849075;81.06951141;121.1004715;107.0855179;316.1758118;93.06972504;145.1009369;133.1010284;316.3000793;131.0855713;105.0695877;159.1166382;147.1170349;109.1010742;299.1992493;67.05410004;69.06990051;149.1323242;160.0757446;299.2391052;173.1326752;187.1481628;271.204071;161.1322327;83.08529663;171.1179199;135.1168671;88.11214447;91.05407715;163.1113892;277.2158203;175.1482849;79.05395508;149.0947266;253.1961975	4126243.367
Helioscopinolide A	317.2091	4.41	C20H28O3	POS	317.2084045;316.2272339;300.2322693;95.08518982;119.0849075;81.06951141;121.1004715;107.0855179;316.1758118;93.06972504;145.1009369;133.1010284;316.3000793;131.0855713;105.0695877;159.1166382;147.1170349;109.1010742;299.1992493;67.05410004;69.06990051;149.1323242;160.0757446;299.2391052;173.1326752;187.1481628;271.204071;161.1322327;83.08529663;171.1179199;135.1168671;88.11214447;91.05407715;163.1113892;277.2158203;175.1482849;79.05395508;149.0947266;253.1961975	4126243.367
(1R,7R,10R)-4,10,11,11-tetramethyltricyclo[5.3.1.01,5]undec-4-en-3-one	219.1735	4.45	C15H22O	POS	219.1737671;93.06958008;81.06964874;131.0851898;91.05395508;107.0852051;79.05403137;95.08522797;121.1008072;67.05407715;119.0851898;117.0695572;145.1008301;105.0695648;135.1164703;109.1007919;107.0488968;133.1008453;201.1634064;159.116394;69.06970978;121.0644531;132.0885925;55.05413437;118.1223373;83.08527374;118.0859756;94.07291412;123.080162;97.10083771;92.05725098;108.0885086;122.	40072638.06

Compound	mz	rt/min	FORMULA	type	MS2 ion fragment	Responses ignal
					104248;146.104248;173.1318359;82.07292938;97.0645752;104.1066895;161.1321564;109.0644989;118.0730972;202.1668091;120.0885162;96.08855438;147.1166534;137.0956421;134.1044159	
α-Cyperone	219.1735	4.45	C15H22O	POS	219.1737671;93.06958008;81.06964874;131.0851898;91.05395508;107.0852051;79.05403137;95.08522797;121.1008072;67.05407715;119.0851898;117.0695572;145.1008301;105.0695648;135.1164703;109.1007919;107.0488968;133.1008453;201.1634064;159.116394;69.06970978;121.0644531;132.0885925;55.05413437;118.1223373;83.08527374;118.0859756;94.07291412;123.080162;97.10083771;92.05725098;108.0885086;122.104248;146.104248;173.1318359;82.07292938;97.0645752;104.1066895;161.1321564;109.0644989;118.0730972;202.1668091;120.0885162;96.08855438;147.1166534;137.0956421;134.1044159	40072638.06
Butyl isobutyl phthalate	279.1581	4.52	C16H22O4	POS	81.06963348;95.0852356;109.1008606;67.05407715;149.0230255;93.06960297;123.1165085;121.100853;107.0852127;278.2183533;135.1165924;79.05407715;69.06972504;136.1198883;261.2203064;122.1042938;137.1323853;94.07293701;83.08535767;108.0885696;149.1322327;57.06976318;119.085228;147.1164856;150.1351318;131.0851898;96.08849335;133.1010437;243.2099915;55.05418015;173.1318359;97.10073853;135.0806885;82.07298279;219.2096863;163.1468658;91.05382538;159.1162109;105.0697327;80.05756378;161.1318054;71.08531189;145.1005707;110.1042557;163.1115112;148.1199188;151.1474152;187.1472931;260.2078552;68.05741882;85.06452179;97.06452179;137.0960846;134.1045074;117.0694885;120.0886002;181.1225891;167.1065369;71.04892731;177.1637115;209.1525269;162.134903;177.1271515;99.08036041;70.07297516;279.1434937;124.120163;165.1273041;92.05742645;139.1116486;111.0803452;195.1376343;84.08847809;125.095932;262.2262573;85.10088348;175.1476746;151.1116638;191.1429291;57.03344727;223.1695404;106.0730133;179.1417847;174.1357117;237.1845093;208.1403046;186.1358032;153.0910034;72.05244446;56.05736542;127.1116333;132.0886688;153.1268921;141.1278534;113.0957336;205.1577148;233.2264404;164.1512909;236.1724396;176.1506195;127.0754776;122.5797272;271.4953613;167.1434326;250.1924286;232.1785278;146.1033783;58.23933411	24255401.34
Mono(2-ethylhexyl) phthalate	279.1581	4.52	C16H22O4	POS	81.06963348;95.0852356;109.1008606;67.05407715;149.0230255;93.06960297;123.1165085;121.100853;107.0852127;278.2183533;135.1165924;79.05407715;69.06972504;136.1198883;261.2203064;122.1042938;137.1323853;94.07293701;83.08535767;108.0885696;149.1322327;57.06976318;119.085228;147.1164856;150.1351318;131.0851898;96.08849335;133.1010437;243.2099915;55.05418015;173.1318359;97.10073853;135.0806885;82.07298279;219.2096863;163.1468658;91.05382538;159.1162109;105.0697327;80.05756378;161.1318054;71.08531189;145.1005707;110.1042557;163.1115112;148.1199188;151.1474152;187.1472931;260.2078552;68.05741882;85.06452179;97.06452179;137.0960846;134.1045074;117.0694885;120.0886002;181.1225891;167.1065369;71.04892731;177.1637115;209.1525269;162.134903;177.1271515;99.08036041;70.07297516;279.1434937;124.120163;165.1273041;92.05742645;139.1116486;111.0803452;195.1376343;84.08847809;125.095932;262.2262573;85.10088348;175.1476746;151.1116638;191.1429291;57.03344727;223.1695404;106.0730133;179.1417847;174.1357117;237.1845093;208.1403046;186.1358032;153.0910034;72.05244446;56.05736542;127.1116333;132.0886688;153.1268921;141.1278534;113.0957336;205.1577148;233.2264404;164.1512909;236.1724396;176.1506195;127.075	24255401.34

Compound	mz	rt/min	FORMULA	type	MS2 ion fragment	Responsesignal
					4776;122.5797272;271.4953613;167.1434326;250.1924286;232.1785278;146.1033783;58.23933411	
Neantine	205.0852	4.52	C12H14O4	POS	149.0230408;204.1771545;121.1009521;149.1322479;135.1166382;95.08525848;107.0852661;93.06965637;204.1383514;81.06970978;109.1009521;123.1165085;148.1198273;67.05425262;79.05401611;57.0697937;187.1472321;120.0890579;69.06995392;150.0265808;83.08547974;134.1043243;119.0855637;105.0700073;110.1045456;124.1204605;147.1169586;122.1048355;96.08883667;106.0729523;133.1010437;163.1484375;145.1012421;136.1196136;137.1324921;131.0854797;91.05423737;108.0888824;55.05430984;159.1173401;97.10123444;82.07335663;162.1369324;166.4304047;179.5731201;178.8760986;60.82505035;196.1669464;74.59070587;98.10639954;70.07310486	5518828.099
all-trans-5,6-Epoxyretinoic acid	317.2076	4.59	C20H28O3	POS	317.2081604;316.2274475;300.2315063;81.06967926;95.08547211;109.1009064;316.2991943;316.178772;133.1012421;131.0854187	29518454.34
Docosahexaenoic acid (DHA)	329.2458	4.59	C22H32O2	POS	102.1274567;329.193573;88.11180878;329.2512207;95.08531189;131.0853577;328.2622375;145.1014099;81.06980133;107.0854263;328.3017578;103.1309586;109.1007767;91.0541687;133.1012878;311.2379456;173.1331329;93.06970215;312.1906128;121.100975;67.0544281;119.0852661;60.08086777;143.0849609;171.1162415;149.1328278;70.06501007;117.0697021;301.2527771;159.1165619;55.0542717;157.1017761;123.1171265;311.1835327;312.23172;105.0695419;69.06995392;201.0707397;57.10332108;83.08521271;157.1348572;209.130127	25151863.92
LPC(18:2/0:0)	520.338	4.68	C26H50NO7P	POS	184.072876;104.1066818;520.3388672;86.09619141;502.3287964;124.9994736;60.08068085;185.0765228;105.1101151;258.110199;337.272583;503.3312683;57.81667709	19017256.36
NAE(18:3(9Z,12Z,15Z))	322.2729	4.75	C20H35NO2	POS	62.05989075;322.273468;95.08525848;81.06963348;261.2207031;67.054039;121.1008759;109.1009216;93.06959534;135.1166534;304.2633972;243.2095947;107.0853043;123.1165771;305.2460938;149.1321106;79.05393219;163.1478119;131.0851288;177.1634827;119.0851212;161.1320801;133.1008148;145.1010284;105.069725;173.1316833;69.06970978;147.1165009;137.1316528;83.08528137;200.1643372;159.1165771;55.05418777;63.06328201;60.04431152;91.05396271;187.1482391;186.1483917	8911838.663
NAE(18:3(6Z,9Z,12Z))	322.2729	4.75	C20H35NO2	POS	62.05989075;322.273468;95.08525848;81.06963348;261.2207031;67.054039;121.1008759;109.1009216;93.06959534;135.1166534;304.2633972;243.2095947;107.0853043;123.1165771;305.2460938;149.1321106;79.05393219;163.1478119;131.0851288;177.1634827;119.0851212;161.1320801;133.1008148;145.1010284;105.069725;173.1316833;69.06970978;147.1165009;137.1316528;83.08528137;200.1643372;159.1165771;55.05418777;63.06328201;60.04431152;91.05396271;187.1482391;186.1483917	8911838.663
NAE(18:2)	324.2886	4.91	C20H37NO2	POS	62.05990982;324.2893066;95.08528137;81.069664;67.05410004;109.1008911;69.0697403;306.2789917;83.08535004;63.06318283;307.2659607;97.10093689;123.1166992;121.1009521;93.0696106;55.05409622;245.2256317;133.1007996;308.2648315;107.085289;263.2362061;119.0850296;79.05406189;57.06978607;147.1168823;96.08857727;135.1163635;82.07311249;161.1319733;149.1323242;137.1321106;105.0698013	144645354.8
Linoleamide	280.2625	4.97	C18H33NO	POS	95.08531189;263.2365112;81.06970215;280.2631226;245.2259827;83.08535767;109.1009369;69.06976318;97.10093689;264.2401428;123.1166687;67.05412292;246.2292938;114.0910873;133.1009369;121.1010361;147.1165161;135.1166534;100.0755081;161.1321564;57.06981277;55.054142;149.1323242;119.0852432;93.06964874;137.1322021;128.1067505;163.1479492;107.0852966;175.147934;165.1636505;179.1791992;71.0853653;151.1478882;111.1165466;97.06447601;8	324735807.3

Compound	mz	rt/min	FORMULA	type	MS2 ion fragment	Responses ignal
					6.05992889;142.1220551;82.07299805;96.08856964;111.0802383;125.0957947;110.1041565;105.0695343;189.1632843;137.095932;156.1378479;151.1117706;91.05418396;139.1114655;124.1203156;79.05413055;84.08861542;184.1695709;170.1535797;70.07291412;134.1048126;162.1358948;221.2256622;165.1277008;85.1010437;85.06480408;98.10426331;164.1516266;140.1061096;99.08009338;198.1847534;181.1588593;148.1203003;219.2103424;122.104454;153.1274109;150.1358643;136.120163;166.1678467;127.1117325;154.1211853;167.1432953;177.1637726;179.1434174;113.0959244;180.1821442;176.1508789;126.0901337;203.1778259;129.1101379;125.1323166;115.0943832;193.1583405;84.08070374;141.1273193;68.05749512;72.08071899;120.0885544;152.151001;138.1361542;71.04894257;108.088356;210.1846924;112.1202927;265.2425232;190.1663666;205.1940918;70.06482697;138.0992889;168.1391754;222.2290497;182.1539612;196.1699677;72.04432678;58.07320404;207.1741943;106.0731583;112.0751801;98.0960083;94.07293701;56.05750275;112.0840302;247.234314;212.2007751;220.2132568;143.1260834;224.2002869;101.0792007;199.1873932;217.1945801;98.06749725;204.180954;191.1790619;98.05987549;171.1570129;262.2521973;166.1304626;152.1153717;72.08869171;218.1967621;180.1467743;213.2017059;185.1729889;169.1421051;112.111351	
PC(16:0/20:4)	782.5706	5.02	C44H80NO8P	POS	184.0728302;86.09616852;782.5670776;102.1274185;124.9996185;185.0762482;88.11188507	652513.1617
NAE(16:0)	300.2887	5.06	C18H37NO2	POS	62.05990219;300.2913513;283.2620544;71.08534241;57.06976318;282.2787476;85.10102844;95.08528137;81.06954193;83.08537292;109.1008987;69.06952667;239.2378693;284.2683105;97.10105896;67.0541687;63.06321716;123.116951	24408632.71
Octaethylene glycol monododecyl ether	539.4191	5.08	C28H58O9	POS	102.1274719;88.11183929;539.3666992;89.05960083;160.0762329;103.1312637;74.09648132;89.11535645;538.3677368;111.4532623;518.9688721;161.4661102	1588308.069
NAE(18:1)	326.3043	5.10	C20H39NO2	POS	62.05990601;326.3054504;309.2774963;83.085289;308.2941589;95.08524323;69.06977081;81.06961823;97.100914;121.1010742;135.1165009;109.1009445;67.05402374;55.05395508;93.06956482;149.1319427;247.2415924;265.2518005;63.06324768;111.1169052;310.2829285;123.1167603;107.0851822	30841559.65
Palmitamide	256.2627	5.11	C16H33NO	POS	256.2629089;88.07545471;102.0910568;57.06978989;74.05988312;71.08535767;116.10672	140888277.1
Oleamide	282.2782	5.14	C18H35NO	POS	282.2785339;83.08527374;97.10082245;69.06970215;57.06973648;95.08519745;247.2413788;81.0696106;111.116478;265.2520447;135.1165009;71.08531952;114.0909882;100.0753937;121.1008377;109.1008377;149.1320648;97.06448364;85.10090637;248.2447815;55.05409241;111.0801544;266.2552185;128.1066437;107.0852127;125.0957565;86.05981445;93.06955719;123.1164398;163.1477356;142.1221619;67.05402374;98.10407257;139.1113739;125.132019;84.08860016;177.1633606;156.1375885;136.1196747;70.07301331;153.1270752;112.1198425;96.08839417;170.1537476;72.08073425;191.1789093;122.1041946;151.147995;150.1355896;165.1639404;137.1324005;79.05390167;184.1689606;110.1040573;115.0944519;82.07301331;167.1428375;181.1581421;72.08854675;164.1513672;198.1848145;212.1996613;72.04407501;240.2682037;205.1946564;226.2162781;112.0836105;101.0787888;129.1099396;113.0959244;108.0886536;58.07309341;209.1892853;99.08007813;195.1742859;140.1147919;124.1199799;192.1821136;178.1666412;126.0991821;56.05752563;240.2317657;143.1260529;94.07296753;213.2043915;171.1568146;267.259613;87.06331635;68.05751801;264.2669983;138.1359253;155.1429443;127.1117783;154.1299744;70.06525421;223.2056427;199.1883392;166.1674347;141.127594;182.1619873;185.172607	100816674.3

Compound	mz	rt/min	FORMULA	type	MS2 ion fragment	Responsesignal
					4;58.06497955	
Stearamide	284.2938	5.32	C18H37NO	POS	284.2942505;88.07546234;102.0911255;116.1067581;57.06976318	8937187.249
DEHP	391.2831	5.35	C24H38O4	POS	149.0230408;71.08538055;167.0337372;57.06978989;150.0266266;390.2918701	26949227.45
Erucamide	338.3406	5.57	C22H43NO	POS	338.341156;97.10083771;83.08525848;69.06968689;321.3147278;303.303894;57.06974792;81.06961823;95.08520508;71.08532715;109.1008987;111.1165009;135.1165771;114.0910492;121.1008301;100.0753555;149.1320801;85.10092163;111.0801773;128.1066437;97.06440735;107.0852203;55.05410767;123.116539;163.147934;125.0957184;142.1224213;93.06958771;139.1113586;86.05975342;67.05408478;125.1323166;156.1380005;177.1631622;153.1268616;198.1841736;137.1321869;184.1695862;170.1541443;205.194458;304.3063049;240.2311859;322.3175354;233.2258148;191.1787567;151.1482697;167.1429443;226.2157898;79.05399323;181.1582642;72.08047485;254.2467804;219.2095184;72.04416656;195.173996;139.1478882;99.07993317;282.2775879;212.1997681;247.2411194;98.10428619;268.2636108;207.2112427;165.1631775;84.08033752;96.08840942;86.09623718;251.2368011;136.1202393;153.1628571;265.2521667;209.1898193;127.1113205;237.2232666;179.1789703;84.08886719;221.2250977;193.1938324;150.1357727;62.59306335;121.874115;239.1282196	57227831
Proline	114.0561	0.23	C5H9NO2	NEG	114.0562592;85.02961731;113.0246124;57.03465271;71.0504303;59.01387024;71.0140686;113.0357895;69.03475952;41.99859619;86.03270721;55.01896286;43.01890564;70.03002167;97.02970123;84.02179718;83.01382446;95.01431274;41.00317001;44.99839783;58.03797913;71.59553528;87.00873566;87.0452652	4798961.625
Glyoxylic acid	72.9932	0.25	C2H2O3	NEG	72.99320221;44.99824142;72.00931549;72.01737976;43.0189209;65.95289612;64.93772888	9238684.191
Pyruvate	87.0088	0.27	C3H4O3	NEG	87.00881195;86.02481079;43.01898193;41.00331879;41.9985733;59.01382065;68.01428986	36707394.88
Phosphate	96.9696	0.27	H3O4P	NEG	96.9602356;78.95908356;96.96950531;69.03462982;96.00930023	16181538.81
Sarcosine	88.0405	0.40	C3H7NO2	NEG	88.04045105;87.00881195;59.01386261;43.01898575;41.00331116;71.01390839;44.01425552;41.9985733	6677245.163
Alanine	88.0405	0.40	C3H7NO2	NEG	88.04045105;87.00881195;59.01386261;43.01898575;41.00331116;71.01390839;44.01425552;41.9985733	6677245.163
beta-Alanine	88.0405	0.40	C3H7NO2	NEG	88.04045105;87.00881195;59.01386261;43.01898575;41.00331116;71.01390839;44.01425552;41.9985733	6677245.163
2-Hydroxyhexanedioic acid	143.035	0.40	C6H10O5	NEG	143.03508;125.0610962;71.0138092;99.08159637;85.02966309;142.0516052;125.0243149;97.06616211;59.01399994;87.04524231;57.0346489;99.04534149;113.0245514;44.99812698;87.00868988;71.05024719;97.02954865;69.03452301;83.05013275;81.03453064;73.02966309;142.0876007;100.0770569;67.35804749;55.01899719;60.01702881;101.024498;83.01365662;75.00865173;66.00017548;44.46287918;64.71129608	12101712.3
3-Hydorxy-3-methylglutaric acid	143.035	0.40	C6H10O5	NEG	143.03508;125.0610962;71.0138092;99.08159637;85.02966309;142.0516052;125.0243149;97.06616211;59.01399994;87.04524231;57.0346489;99.04534149;113.0245514;44.99812698;87.00868988;71.05024719;97.02954865;69.03452301;83.05013275;81.03453064;73.02966309;142.0876007;100.0770569;67.35804749;55.01899719;60.01702881;101.024498;83.01365662;75.00865173;66.00017548;44.46287918;64.71129608	12101712.3
D-Pinitol	193.0717	0.40	C7H14O6	NEG	161.045639;193.0737;101.0244446;192.059433;59.01385498;73.02954102;85.02957153;99.00885773;87.00870514;71.01387024;133.1061096;141.0195007;125.0243835;97.02958679;113.0243607;83.0139389;44.99824142;155.0350494;178.026886;89.02452087;69.034729;175.0614777;134.0375519;81.03455353;57.03472519;95.01407623;159.030304;55.01912689;149.0611115;86.03305817;142.0277557;191.0558014;111.0090027;117.0195847;140.0113525;115.0404816;127.0404	13762388.73

Compound	mz	rt/min	FORMULA	type	MS2 ion fragment	Responsesignal
					51;123.0090103;150.9486084;135.0297546;148.8910675;79.01889801;131.0355225;75.00863647;103.0040588;143.034729;188.0972748;46.02157974;74.56755829;73.78886414;121.0294418;151.3209076;92.4324646;72.46144104;96.63103485	
Methyl β-D-Galactopyranoside	193.0717	0.40	C7H14O6	NEG	161.045639;193.0737;101.0244446;192.059433;59.01385498;73.02954102;85.02957153;99.00885773;87.00870514;71.01387024;133.1061096;141.0195007;125.0243835;97.02958679;113.0243607;83.0139389;44.99824142;155.0350494;178.026886;89.02452087;69.034729;175.0614777;134.0375519;81.03455353;57.03472519;95.01407623;159.030304;55.01912689;149.0611115;86.03305817;142.0277557;191.0558014;111.0090027;117.0195847;140.0113525;115.0404816;127.040451;123.0090103;150.9486084;135.0297546;148.8910675;79.01889801;131.0355225;75.00863647;103.0040588;143.034729;188.0972748;46.02157974;74.56755829;73.78886414;121.0294418;151.3209076;92.4324646;72.46144104;96.63103485	13762388.73
Asparagine	131.0462	0.40	C4H8N2O3	NEG	114.0198364;113.0357742;131.0462189;130.0875092;70.02990723;73.02958679;95.02519989;72.00920868;71.02514648;85.02947235;41.99853897;59.0138588;87.04512787;58.02970123;88.04072571;101.0243301;71.01390076;85.06564331;75.00869751;57.03448486;115.0232773	4763921.861
Diglycine	131.0462	0.40	C4H8N2O3	NEG	114.0198364;113.0357742;131.0462189;130.0875092;70.02990723;73.02958679;95.02519989;72.00920868;71.02514648;85.02947235;41.99853897;59.0138588;87.04512787;58.02970123;88.04072571;101.0243301;71.01390076;85.06564331;75.00869751;57.03448486;115.0232773	4763921.861
Xylose	131.0351	0.41	C5H10O5	NEG	113.0356522;114.0197906;130.0873718;73.02951813;131.0347595;70.02989197;85.02955627;95.02516937;88.04050446;87.04510498;72.00898743;59.01390076;41.99856567;71.02508545;57.03452301;101.0244751;58.02987289;75.00897217;71.01378632;115.0035019;100.9335785;69.03452301;86.06111908;44.9981575;89.03547668;115.022438;55.01900482;60.60174179;52.58994675	11811132.55
Fructose	179.0561	0.42	C6H12O6	NEG	59.01386261;179.0558929;89.02445984;161.0455322;71.01391602;87.00878906;85.02957153;75.00886536;101.024559;117.0191574;99.0089035;118.9929504;125.0242615;73.0296402;113.0244675;96.96962738;44.99821854;119.0348892;141.0195618;164.0117645;97.02947998;159.0296021;178.0874634;83.01376343;163.0398712;69.03433228;95.01376343;151.0401306;177.0386505;57.0344429;41.00327301;81.03464508;43.01905441;143.0339661;96.96067047;131.0345154;65.44882202;99.51197815;54.89618301	19656536.12
Mannose	179.0561	0.42	C6H12O6	NEG	59.01386261;179.0558929;89.02445984;161.0455322;71.01391602;87.00878906;85.02957153;75.00886536;101.024559;117.0191574;99.0089035;118.9929504;125.0242615;73.0296402;113.0244675;96.96962738;44.99821854;119.0348892;141.0195618;164.0117645;97.02947998;159.0296021;178.0874634;83.01376343;163.0398712;69.03433228;95.01376343;151.0401306;177.0386505;57.0344429;41.00327301;81.03464508;43.01905441;143.0339661;96.96067047;131.0345154;65.44882202;99.51197815;54.89618301	19656536.12
Galactinol	341.1088	0.42	C12H22O11	NEG	89.02442169;59.0138588;71.01387787;119.034996;101.0244217;341.1088562;179.0561218;113.0244446;161.0457306;143.0349884;131.0350342;149.045639;85.02966309;87.00866699;90.02798462;95.0139389;73.02957153;60.01712036	18290703.17
Turanose	341.1088	0.42	C12H22O11	NEG	89.02442169;59.0138588;71.01387787;119.034996;101.0244217;341.1088562;179.0561218;113.0244446;161.0457306;143.0349884;131.0350342;149.045639;85.02966309;87.00866699;90.02798462;95.0139389;73.02957153;60.01712036	18290703.17
Sucrose	341.1088	0.42	C12H22O11	NEG	89.02442169;59.0138588;71.01387787;119.034996;101.0244217;341.1088562;179.0561218;113.0244446;161.0457306;143.0349884;131.035034	18290703.17

Compound	mz	rt/min	FORMULA	type	MS2 ion fragment	Responsesignal
					2;149.045639;85.02966309;87.00866699;90.02798462;95.0139389;73.02957153;60.01712036	
Palatinose (hydrate)	341.1088	0.42	C12H22O11	NEG	89.02442169;59.0138588;71.01387787;119.034996;101.0244217;341.1088562;179.0561218;113.0244446;161.0457306;143.0349884;131.0350342;149.045639;85.02966309;87.00866699;90.02798462;95.0139389;73.02957153;60.01712036	18290703.17
(3S,4R,5R)-1,3,4,5-tetrahydroxy-6-[(2S,3R,4S,5S,6R)-3,4,5-trihydroxy-6-(hydroxymethyl)tetrahydropyran-2-yl]oxy-hexan-2-one	341.1088	0.42	C12H22O11	NEG	89.02442169;59.0138588;71.01387787;119.034996;101.0244217;341.1088562;179.0561218;113.0244446;161.0457306;143.0349884;131.0350342;149.045639;85.02966309;87.00866699;90.02798462;95.0139389;73.02957153;60.01712036	18290703.17
(2R,3S,4R,5R)-2,3,4,5-tetrahydroxy-6-[(2R,3R,4S,5S,6R)-3,4,5-trihydroxy-6-(hydroxymethyl)tetrahydropyran-2-yl]oxy-hexanal	341.1088	0.42	C12H22O11	NEG	89.02442169;59.0138588;71.01387787;119.034996;101.0244217;341.1088562;179.0561218;113.0244446;161.0457306;143.0349884;131.0350342;149.045639;85.02966309;87.00866699;90.02798462;95.0139389;73.02957153;60.01712036	18290703.17
(2R,3S,4S,5R,6R)-2-(hydroxymethyl)-6-[(2S,3R,4S,5S,6R)-3,4,5-trihydroxy-6-(hydroxymethyl)tetrahydropyran-2-yl]oxy-tetrahydropyran-3,4,5-triol	341.1088	0.42	C12H22O11	NEG	89.02442169;59.0138588;71.01387787;119.034996;101.0244217;341.1088562;179.0561218;113.0244446;161.0457306;143.0349884;131.0350342;149.045639;85.02966309;87.00866699;90.02798462;95.0139389;73.02957153;60.01712036	18290703.17
Trehalose	341.1088	0.42	C12H22O11	NEG	89.02442169;59.0138588;71.01387787;119.034996;101.0244217;341.1088562;179.0561218;113.0244446;161.0457306;143.0349884;131.0350342;149.045639;85.02966309;87.00866699;90.02798462;95.0139389;73.02957153;60.01712036	18290703.17
Isomaltose	341.1088	0.42	C12H22O11	NEG	89.02442169;59.0138588;71.01387787;119.034996;101.0244217;341.1088562;179.0561218;113.0244446;161.0457306;143.0349884;131.0350342;149.045639;85.02966309;87.00866699;90.02798462;95.0139389;73.02957153;60.01712036	18290703.17
3-Pyridol	94.0299	0.43	C5H5NO	NEG	94.02987671	3755606.47
Valine	116.0717	0.43	C5H11NO2	NEG	73.02955627;116.0718613;59.01386261;115.0402603;99.00895691;115.0765533;71.0140152;71.05021667;99.92590332;55.01892471;97.02988434;72.04557037;98.02461243;74.02470398;88.04066467;111.7101822;43.63993454;48.53553391	5086101.077
4-(Methylamino)butanoic acid	116.0717	0.43	C5H11NO2	NEG	73.02955627;116.0718613;59.01386261;115.0402603;99.00895691;115.0765533;71.0140152;71.05021667;99.92590332;55.01892471;97.02988434;72.04557037;98.02461243;74.02470398;88.04066467;111.7101822;43.63993454;48.53553391	5086101.077
5-Aminopentanoic acid	116.0717	0.43	C5H11NO2	NEG	73.02955627;116.0718613;59.01386261;115.0402603;99.00895691;115.0765533;71.0140152;71.05021667;99.92590332;55.01892471;97.02988434;72.04557037;98.02461243;74.02470398;88.04066467;111.7101822;43.63993454;48.53553391	5086101.077
(S)-3,4-Dihydroxybutyric acid (lithium hydrate)	119.035	0.44	C4H8O4	NEG	59.01387405;119.0352554;74.02482605;71.01393127;75.00894928;118.0509872;61.98842239;101.02491;89.02454376;73.02968597;55.01911545;83.01396179;57.03453064;45.99340057;57.24157715;89.40660095;49.30751801;56.39379501	10371942.73
2,4-Dihydroxybutanoic acid	119.035	0.44	C4H8O4	NEG	59.01387405;119.0352554;74.02482605;71.01393127;75.00894928;118.0509872;61.98842239;101.02491;89.02454376;73.02968597;55.01911545;83.01396179;57.03453064;45.99340057;57.24157715;89.40660095;49.30751801;56.39379501	10371942.73
Purine	119.035	0.44	C5H4N4	NEG	59.01387405;119.0352554;74.02482605;71.01393127;75.00894928;118.0509872;61.98842239;101.02491;89.02454376;73.02968597;55.01911545;83.01396179;57.03453064;45.99340057;57.24157715;89.40660095;49.30751801;56.39379501	10371942.73
D-Mannoheptulose	191.0561	0.44	C7H14O7	NEG	191.0560455;85.02954865;111.0088425;133.1063538;87.0087738;59.01386642;127.0401001;93.03457642;173.0458984	26751265.28
3-Amino-4-methylpentanoic acid	130.0874	0.45	C6H13NO2	NEG	130.0874329;114.0196915;113.0357742;73.02946472;85.02941895;57.03450775;86.06138611;87.04547882;59.01383972;88.04063416;70.0298996;85.06580353;129.0558014;101.0244522;112.0405731;95.02516937;58.02976608;129.0914764;41.99841309;71.02536774;69.03436279;129.0389709;71.01387787;72.00904083;75.00878906;83	5097974.539

Compound	mz	rt/min	FORMULA	type	MS2 ion fragment	Responsesignal
					.04984283	
Norleucine	130.0874	0.45	C6H13NO2	NEG	130.0874329;114.0196915;113.0357742;73.02946472;85.02941895;57.03450775;86.06138611;87.04547882;59.01383972;88.04063416;70.0298996;85.06580353;129.0558014;101.0244522;112.0405731;95.02516937;58.02976608;129.0914764;41.99841309;71.02536774;69.03436279;129.0389709;71.01387787;72.00904083;75.00878906;83.04984283	5097974.539
Leucine	130.0874	0.45	C6H13NO2	NEG	130.0874329;114.0196915;113.0357742;73.02946472;85.02941895;57.03450775;86.06138611;87.04547882;59.01383972;88.04063416;70.0298996;85.06580353;129.0558014;101.0244522;112.0405731;95.02516937;58.02976608;129.0914764;41.99841309;71.02536774;69.03436279;129.0389709;71.01387787;72.00904083;75.00878906;83.04984283	5097974.539
Isoleucine	130.0874	0.45	C6H13NO2	NEG	130.0874329;114.0196915;113.0357742;73.02946472;85.02941895;57.03450775;86.06138611;87.04547882;59.01383972;88.04063416;70.0298996;85.06580353;129.0558014;101.0244522;112.0405731;95.02516937;58.02976608;129.0914764;41.99841309;71.02536774;69.03436279;129.0389709;71.01387787;72.00904083;75.00878906;83.04984283	5097974.539
Caplamin	130.0874	0.45	C6H13NO2	NEG	130.0874329;114.0196915;113.0357742;73.02946472;85.02941895;57.03450775;86.06138611;87.04547882;59.01383972;88.04063416;70.0298996;85.06580353;129.0558014;101.0244522;112.0405731;95.02516937;58.02976608;129.0914764;41.99841309;71.02536774;69.03436279;129.0389709;71.01387787;72.00904083;75.00878906;83.04984283	5097974.539
Glycolate	75.0088	0.45	C2H4O3	NEG	75.00880432;72.99317169;74.0247879	22644256.42
Lactate	89.0245	0.46	C3H6O3	NEG	89.02445984;43.01898575;59.01387024;88.04051971;71.01386261;41.00333786	72620636.76
Glyceraldehyde	89.0245	0.46	C3H6O3	NEG	89.02445984;43.01898575;59.01387024;88.04051971;71.01386261;41.00333786	72620636.76
Dihydroxyacetone	89.0245	0.46	C3H6O3	NEG	89.02445984;43.01898575;59.01387024;88.04051971;71.01386261;41.00333786	72620636.76
Pyruvaldehyde	71.0139	0.48	C3H4O2	NEG	71.01393127;41.00334549;43.01898193	12767635.55
Methylmalonic acid	117.0194	0.49	C4H6O4	NEG	116.9286804;73.02954865;117.0194168;116.0717926;99.00882721;99.92591858;59.01385117	14872231.24
Succinate	117.0194	0.49	C4H6O4	NEG	116.9286804;73.02954865;117.0194168;116.0717926;99.00882721;99.92591858;59.01385117	14872231.24
Galactose 1-phosphate	259.0223	0.50	C6H13O9P	NEG	78.95907593;259.0222168;96.96961212;223.0020294;241.0126801;221.8414307;134.894577;223.8397522;133.106369;191.0558319;127.8699875;198.9915619	988372.7193
Glyceric acid	105.0193	0.51	C3H6O4	NEG	105.0194473;75.00878906;59.01385498;44.99815369;74.0248642;104.0352859;72.99321747;56.99810028;72.00950623;104.043663	9574779.821
sn-Glycerol 3-phosphate	171.0064	0.52	C3H9O6P	NEG	78.9590683;171.0068207;170.8334045;127.1128006;96.96961975;125.0976181;127.0766602;89.02453613;109.06604;153.0931244;99.0820694	975226.1311
Pyroglutamic acid	128.0354	0.52	C5H7NO3	NEG	128.0354156;85.02957153;127.0404053;57.03467178;101.0243988;55.01887512;127.0036011;59.01386642;73.02955627;83.05019379	24647911.54
Dimethadione	128.0354	0.52	C5H7NO3	NEG	128.0354156;85.02957153;127.0404053;57.03467178;101.0243988;55.01887512;127.0036011;59.01386642;73.02955627;83.05019379	24647911.54
Malic acid	133.0143	0.53	C4H6O5	NEG	115.0038376;133.0144348;71.01391602;72.99315643;89.02440643;75.00880432;80.965271;87.1006546;43.01898956;116.0073624;132.0999603;88.04031372;87.00908661;132.0301819;116.079071;85.08525848;87.04495239	21138336.64
Citric Acid	191.0198	0.53	C6H8O7	NEG	111.0087967;85.02949524;87.00875854;191.0195618;133.1062164;145.8625183;127.0399399;144.8660583;59.01380157;93.03478241;57.03446198;129.0193176;44.9982338;173.0453033;61.98832703;173.0090637;71.01387024;143.8652191;112.0122223;176.0115051;171.0300903;163.8728333;101.0243759;99.04541016;78.95890045;109.0301208;67.01899719;90.71437836;83.04979706;158.3726807;119.9253159;122.5343704	4824290.774
1,5-Anhydroglucitol	163.0612	0.67	C6H12	NEG	163.0614929;162.8393097;101.0245361;59.0138	6801640.7

Compound	mz	rt/min	FORMULA	type	MS2 ion fragment	Responsesignal
			O5		855;61.02953339;72.99308777;119.0503922;75.00878906;147.0454102;85.02967072;72.00926971;91.05554962;135.044693;57.03493881;102.028183;89.02479553;134.0375366;51.87244797;62.03287506;47.55602264;47.05875397;71.01434326;74.67336273	09
Mevalonic acid	147.0664	0.68	C6H12O4	NEG	59.01387787;147.0664825;87.04522705;57.03456879;85.06588745;129.0553131;101.0610352;61.98852921;99.00900269;85.02974701;129.0197601;75.00878143;89.02475739;71.0139389;73.02936554;44.99833298;50.77872849;53.99124146;56.49211884;119.0506439;80.8565979	692468.9978
3,6,9-Trioxaundecanedioic acid	221.0667	0.69	C8H14O7	NEG	119.0350723;59.0139389;163.0610657;221.0669861;75.00888062;162.8392487;176.9411011;101.0249176;161.0037079;221.0254364;220.9338684;202.8872375;124.0412292;71.01396179;179.1078491;184.8766327;111.4502869;164.9314575;121.9454422;107.226387;206.7860107;62.77288055	11502434.12
Phenylalanine	164.0718	0.70	C9H11NO2	NEG	147.0453949;164.0719147;72.00917053;59.01383591;163.061264;119.0503311;101.0240936;150.0329285;103.0557098;121.0660782;93.0345993;61.02954483;121.0296249;75.00894928;163.0774384;91.05521393;163.039032;148.049408;85.02976227;120.0457458;72.99314117;94.02938843;52.88581467;87.008255;61.91124725;61.07467651	1735530.491
Glutaric acid	131.0351	0.70	C5H8O4	NEG	130.0874481;87.04524231;73.02954865;131.0351257;85.02953339;59.01392365;113.0246277;101.0248718;57.03470993;85.06583405;88.0404892;75.00891113;114.0193634;69.03456116;113.0357971;71.0139389;112.0402908;86.06110382;44.99828339;89.02407074;44.01431656;76.92909241;84.27475739;115.0037766;72.51476288;70.92175293	17670802.83
Methylsuccinic acid	131.0351	0.70	C5H8O4	NEG	130.0874481;87.04524231;73.02954865;131.0351257;85.02953339;59.01392365;113.0246277;101.0248718;57.03470993;85.06583405;88.0404892;75.00891113;114.0193634;69.03456116;113.0357971;71.0139389;112.0402908;86.06110382;44.99828339;89.02407074;44.01431656;76.92909241;84.27475739;115.0037766;72.51476288;70.92175293	17670802.83
2-Ketobutyric acid	101.0245	0.77	C4H6O3	NEG	73.02957153;101.0245438;59.01387787;57.03462219;55.01900864;83.01396942;43.0189743;58.00605774;74.03316498;41.00336456;71.0139389	173403727.3
Propionic acid	73.0296	0.79	C3H6O2	NEG	73.0295639;72.99324799;44.99822617	9591781.283
Hydroxyacetone	73.0296	0.79	C3H6O2	NEG	73.0295639;72.99324799;44.99822617	9591781.283
3-Hydroxyvaleric acid	117.0558	0.91	C5H10O3	NEG	116.9287262;73.02957153;59.01388931;116.0718765;99.92588806;99.00889587;71.0502243;71.01413727	3247676.79
5-ketocaproate	129.0559	0.97	C6H10O3	NEG	129.0559692;59.01392746;57.03464508;128.0354309;129.0195618;85.02955627;85.06587219;128.0718079;111.0454483;83.05027008;73.02954865;55.01900482;101.0245285;71.01393127;111.0089722;68.99824524;99.00941467;86.06119537;44.99834824;87.00906372;72.99324036;42.41983032;102.2197037;48.93334961	17909757.54
4-Oxohexanoic acid	129.0558	1.36	C6H10O3	NEG	129.0559235;85.065979;128.0354309;129.0196228;111.0454712;57.0346489;85.02960968;59.01389313;83.05020142;73.02955627;128.0718842;55.01879883;101.0246429;87.00871277;68.99827576;67.0190506;88.04024506;71.01399994;86.06967163;99.0093689;44.9982338;41.00331879;112.0400696;41.99884415;61.95458984;49.47343826;117.0899963;125.762764;47.30423355;41.29431534	22218823.2
3-Methyl-2-oxovaleric acid	129.0558	1.36	C6H10O3	NEG	129.0559235;85.065979;128.0354309;129.0196228;111.0454712;57.0346489;85.02960968;59.01389313;83.05020142;73.02955627;128.0718842;55.01879883;101.0246429;87.00871277;68.99827576;67.0190506;88.04024506;71.01399994;86.06967163;99.0093689;44.9982338;41.00331879;112.0400696;41.99884415;61.95458984;49.47343826;117.0899963;125.762764;47.30423355;41.	22218823.2

Compound	mz	rt/min	FORMULA	type	MS2 ion fragment	Responsesignal
					29431534	
Ketoleucine	129.0558	1.36	C6H10O3	NEG	129.0559235;85.065979;128.0354309;129.0196228;111.0454712;57.0346489;85.02960968;59.01389313;83.05020142;73.02955627;128.0718842;55.01879883;101.0246429;87.00871277;68.99827576;67.0190506;88.04024506;71.01399994;86.06967163;99.0093689;44.9982338;41.00331879;112.0400696;41.99884415;61.95458984;49.47343826;117.0899963;125.762764;47.30423355;41.29431534	22218823.2
Pyrocatechol	109.0296	1.79	C6H6O2	NEG	109.0296783;108.0455551;66.0349884;108.0218887;81.03434753	7383259.621
Hydroquinone	109.0296	1.79	C6H6O2	NEG	109.0296783;108.0455551;66.0349884;108.0218887;81.03434753	7383259.621
Butanoic acid	87.0453	2.16	C4H8O2	NEG	87.00883484;87.04518127;86.02483368;43.01899338;41.00333405;41.99852371;59.01384735	3990964.709
Vanillic acid	167.0352	2.41	C8H8O4	NEG	167.03479;109.0297546;152.011795;123.0454102;108.0222015;149.0245972;123.0815506;139.0398254;41.99847031;166.0505981;137.0241852;121.0288925;135.0087128;132.868454;125.0241776;120.8993454;81.03475189;122.0322876;49.26750946	7116553.227
Terephthalic-Acid	165.0195	2.46	C8H6O4	NEG	121.0296631;147.0817413;164.8364105;93.03469086;109.0660934;165.0198364;136.0529938;137.09729;147.0453949;69.03471375;135.0818329;164.071106;150.0322571;122.0331497;44.99818802;137.0613098;95.05025482;135.045105;77.03979492;119.086731;108.0578537;106.0429993;132.0583649;119.0503922;91.0554657;107.0500031;75.00888062;72.00917816;59.01387024;148.0852966	6556237.12
Phthalic acid	165.0195	2.46	C8H6O4	NEG	121.0296631;147.0817413;164.8364105;93.03469086;109.0660934;165.0198364;136.0529938;137.09729;147.0453949;69.03471375;135.0818329;164.071106;150.0322571;122.0331497;44.99818802;137.0613098;95.05025482;135.045105;77.03979492;119.086731;108.0578537;106.0429993;132.0583649;119.0503922;91.0554657;107.0500031;75.00888062;72.00917816;59.01387024;148.0852966	6556237.12
Piperonylic acid	165.0195	2.46	C8H6O4	NEG	121.0296631;147.0817413;164.8364105;93.03469086;109.0660934;165.0198364;136.0529938;137.09729;147.0453949;69.03471375;135.0818329;164.071106;150.0322571;122.0331497;44.99818802;137.0613098;95.05025482;135.045105;77.03979492;119.086731;108.0578537;106.0429993;132.0583649;119.0503922;91.0554657;107.0500031;75.00888062;72.00917816;59.01387024;148.0852966	6556237.12
3-Hydroxybenzoic acid	137.0246	2.48	C7H6O3	NEG	93.03463745;137.0245514;108.0215454;109.0298386;94.03830719;136.0404663;94.03048706;95.05043793;59.0138855;81.03465271;108.0455475;109.039711;78.63521576	20658122.56
4-Hydroxybenzoic acid	137.0246	2.48	C7H6O3	NEG	93.03463745;137.0245514;108.0215454;109.0298386;94.03830719;136.0404663;94.03048706;95.05043793;59.0138855;81.03465271;108.0455475;109.039711;78.63521576	20658122.56
Hydroxyisocaproic acid	131.0715	2.50	C6H12O3	NEG	59.0138855;131.0715179;130.0874329;73.02961731;131.0351715;85.02952576;87.04524231;113.0248337;85.06562805;57.03449631;101.0246506;88.0404129;86.06136322;71.01416016;75.00895691;69.03465271;113.0339127;113.0612411	13484921.78
3-(3-Hydroxyphenyl)propanoic acid	165.056	2.56	C9H10O3	NEG	121.0296555;93.03463745;147.081665;121.0659943;165.0556946;164.8364258;109.06604;136.0531311;165.0198822;95.05037689;135.0451508;69.03466797;137.09729;137.0608978;44.99825287;150.0327148;135.0812683;164.0721893;122.0334549;77.03993225;132.058075;106.0424118;119.0868607;75.00894928;43.01896667;108.058197;122.0696335;91.05561829;94.03784943;137.0248566;118.8999557;148.0850677;119.0507431;59.0138588	2261152.094
2-Phenyllactic acid	165.056	2.56	C9H10O3	NEG	121.0296555;93.03463745;147.081665;121.0659943;165.0556946;164.8364258;109.06604;136.0531311;165.0198822;95.05037689;135.0451508;69.03466797;137.09729;137.0608978;44.99825287;150.0327148;135.0812683;164.0721893;122.0334549;77.03993225;132.058075;106.0424118;	2261152.094

Compound	mz	rt/min	FORMULA	type	MS2 ion fragment	Responsesignal
					119.0868607;75.00894928;43.01896667;108.058197;122.0696335;91.05561829;94.03784943;137.0248566;118.8999557;148.0850677;119.0507431;59.0138588	
Veratric acid	181.049	2.60	C9H10O4	NEG	137.0609894;125.060936;89.02451324;59.01388931;101.0245972;119.0503693;71.01399231;109.0661011;81.034729;109.0296097;181.0499878;163.0769958;166.02742;80.02674866;108.0217361;139.076355;111.045433;73.02961731;163.0610809;96.02150726;85.02970123;113.024971;93.07120514;93.03464508;95.05043793;122.0376358;43.01903152;57.03479004;97.02959442;121.0662003;146.868454;44.99819183;97.06591797;83.01412964;107.0500412;135.0821533;135.0456848;131.0354767;94.03010559;138.0649414;115.0401154;69.0346756;143.8657379;120.9887924;151.0040436;149.0467072;55.0189476;83.05045319;134.037674;144.8722992;56.49669647;152.9406738;90.02742004;129.2163849;86.54283905;116.1464081	888301.6139
Idramantone	165.0922	2.61	C10H14O2	NEG	165.0921631;121.0296478;109.0660934;136.0531158;121.0659637;147.081604;93.03466034;137.0973969;69.03463745;147.0455322;150.0324402;135.0816803;44.99826813;137.0608521;119.050415;135.0451965;164.072403;108.0582581;95.0503006;119.0868225	2150350.489
Pimelic acid	159.0665	2.61	C7H12O4	NEG	97.06598663;159.066452;115.0766373;159.0305328;100.9337692;73.02958679;95.05041504;141.055954;129.019455;158.0801849;101.024292;111.0088654;158.0629272;59.01407242;87.00904083;113.0246811;116.9286499;98.06938171;99.00908661;57.03463364;71.01383972;85.02961731;158.1273193;113.0975113;114.0928421;128.051239	55948876.29
3-Methyladipic acid	159.0665	2.61	C7H12O4	NEG	97.06598663;159.066452;115.0766373;159.0305328;100.9337692;73.02958679;95.05041504;141.055954;129.019455;158.0801849;101.024292;111.0088654;158.0629272;59.01407242;87.00904083;113.0246811;116.9286499;98.06938171;99.00908661;57.03463364;71.01383972;85.02961731;158.1273193;113.0975113;114.0928421;128.051239	55948876.29
2-Hydroxy-6-methoxybenzoic acid	167.0352	2.63	C8H8O4	NEG	152.0116425;167.0348816;123.045311;108.0217972;91.01878357;166.0512085;153.0149231;125.0244064;149.0610046;59.01373672;95.05064392;139.0390778;59.05046082;122.0610199	12738027.99
2-Hydroxy-4-methoxybenzoic acid	167.0352	2.63	C8H8O4	NEG	152.0116425;167.0348816;123.045311;108.0217972;91.01878357;166.0512085;153.0149231;125.0244064;149.0610046;59.01373672;95.05064392;139.0390778;59.05046082;122.0610199	12738027.99
5-Methoxysalicylic acid	167.0352	2.63	C8H8O4	NEG	152.0116425;167.0348816;123.045311;108.0217972;91.01878357;166.0512085;153.0149231;125.0244064;149.0610046;59.01373672;95.05064392;139.0390778;59.05046082;122.0610199	12738027.99
2-hydroxy-3,4-dimethoxy-benzoic acid	197.0457	2.65	C9H10O5	NEG	162.8391724;182.0221405;197.0454865;160.8421936;153.0559387;166.9986725;121.0296173;138.0323944;123.0089188;169.1231232;183.0254364;161.8423157;108.0452194;152.0358887;166.0584717;151.0347595;196.1350403;69.03453064	6634563.756
Suberic acid	173.0821	2.67	C8H14O4	NEG	59.01388931;173.082077;111.0816803;113.0609818;172.1066895;93.03460693;155.0717468;95.05046082	8659764.699
2-tert-butyl-1,4-benzoquinone	163.0767	2.68	C10H12O2	NEG	163.0765228;134.0375061;162.839325;135.0816956;119.0503235;147.0450592;59.01387405;106.0427933;72.0090332;120.0579224;101.0244904;91.055336;136.085144;85.02957153;107.0504227	2181015.199
3-hydroxybenzaldehyde	121.0297	2.70	C7H6O2	NEG	121.0296555	50515781.59
4-Hydroxybenzaldehyde	121.0297	2.70	C7H6O2	NEG	121.0296555	50515781.59
2-Ethyl-2-hydroxybutyric acid	131.0716	2.73	C6H12O3	NEG	131.0714874;85.0659256;130.0874939;73.02953339;59.01389694;87.0451889;113.060791;113.025116;101.0248413;57.03452301;129.056076;75.00884247;86.0694809;86.06118011;88.0404129	36483373.02
4-(3-hydroxyprop-1-enyl)-2,6-dimethoxy-phenol	209.0821	2.75	C11H14O4	NEG	121.0296478;194.058548;179.0350952;161.0246124;209.0818939;176.0479279;209.0453186;59.	2904250.593

Compound	mz	rt/min	FORMULA	type	MS2 ion fragment	Responsesignal
					01398849;191.0711517;120.0213928;124.040535;164.9414825;122.0330963;195.0619049;151.0403442;165.0553894;69.36632538;80.62268066;62.94742203	
Sinapyl alcohol	209.0821	2.75	C11H14O4	NEG	121.0296478;194.058548;179.0350952;161.0246124;209.0818939;176.0479279;209.0453186;59.01398849;191.0711517;120.0213928;124.040535;164.9414825;122.0330963;195.0619049;151.0403442;165.0553894;69.36632538;80.62268066;62.94742203	2904250.593
3-(2,5-Dimethoxyphenyl)propionic acid	209.0821	2.75	C11H14O4	NEG	121.0296478;194.058548;179.0350952;161.0246124;209.0818939;176.0479279;209.0453186;59.01398849;191.0711517;120.0213928;124.040535;164.9414825;122.0330963;195.0619049;151.0403442;165.0553894;69.36632538;80.62268066;62.94742203	2904250.593
1-(4-hydroxy-3,5-dimethoxy-phenyl)propan-1-one	209.0821	2.75	C11H14O4	NEG	121.0296478;194.058548;179.0350952;161.0246124;209.0818939;176.0479279;209.0453186;59.01398849;191.0711517;120.0213928;124.040535;164.9414825;122.0330963;195.0619049;151.0403442;165.0553894;69.36632538;80.62268066;62.94742203	2904250.593
2-Carboxybenzaldehyde	149.0247	2.76	C8H6O3	NEG	149.0244904;105.0347137;131.050354;121.0295486;89.02453613;95.05032349;134.0369873;121.065979;59.01385117;106.0423813;77.03977966;130.0425568;107.0502701;43.01903152;93.0344162;123.0452728;71.01370239;69.0348053	1310230.242
2',4',6'-Trihydroxyacetophenone	149.0247	2.76	C8H8O4	NEG	149.0244904;105.0347137;131.050354;121.0295486;89.02453613;95.05032349;134.0369873;121.065979;59.01385117;106.0423813;77.03977966;130.0425568;107.0502701;43.01903152;93.0344162;123.0452728;71.01370239;69.0348053	1310230.242
3,4-Dihydroxyphenylacetic acid	149.0247	2.76	C8H8O4	NEG	149.0244904;105.0347137;131.050354;121.0295486;89.02453613;95.05032349;134.0369873;121.065979;59.01385117;106.0423813;77.03977966;130.0425568;107.0502701;43.01903152;93.0344162;123.0452728;71.01370239;69.0348053	1310230.242
(2S,3S,4aS)-2,3,7-trihydroxy-9-methoxy-4a-methyl-3,4-dihydro-2H-benzo[c]chromen-6-one	291.0878	2.76	C15H16O6	NEG	291.0875549;217.0870819;247.09758;189.0922394;201.0923615;229.0870667;218.0900574;199.0758972;173.0971375;245.0832367;219.1012421;248.1012573;171.0817566;59.01386642;71.01387787;202.0637817;215.0706177;161.0971375;137.0613403;163.0394287;190.0948486;203.0710297;135.0456543;263.0925598;188.0474854;174.0684052;187.0762024;162.8390045;160.0531006;58.33828354;136.1366882;137.0240631;71.31994629;77.42198944;233.0811768;108.6706238	693732.0528
(1-Hydroxycyclohexyl)acetic acid	157.0872	2.80	C8H14O3	NEG	157.0870667;100.9337311;97.06600189;139.0765533;113.0973282;57.03462601;59.01385117;73.02954865;116.928688;111.0819626;115.0763397;129.0197296;140.0802765;98.06926727;69.03468323;114.9491882;114.1012573;115.920517;95.05034637;99.00900269;111.0089417;114.0923309	39161155.24
5-Oxooctanoic acid	157.0872	2.80	C8H14O3	NEG	157.0870667;100.9337311;97.06600189;139.0765533;113.0973282;57.03462601;59.01385117;73.02954865;116.928688;111.0819626;115.0763397;129.0197296;140.0802765;98.06926727;69.03468323;114.9491882;114.1012573;115.920517;95.05034637;99.00900269;111.0089417;114.0923309	39161155.24
3,5-Dimethoxybenzoic acid	181.0509	2.81	C9H10O4	NEG	166.0271759;181.0509033;151.0037842;109.0296402;167.0305481;89.02446747;59.0138855;101.0246048;137.0609131;71.01392365	50949072.7
ethyl 3,5-dihydroxybenzoate	181.0509	2.81	C9H10O4	NEG	166.0271759;181.0509033;151.0037842;109.0296402;167.0305481;89.02446747;59.0138855;101.0246048;137.0609131;71.01392365	50949072.7
3,4-Dihydroxyhydrocinnamic acid	181.0509	2.81	C9H10O4	NEG	166.0271759;181.0509033;151.0037842;109.0296402;167.0305481;89.02446747;59.0138855;101.0246048;137.0609131;71.01392365	50949072.7
3,5-Dihydroxyacetophenone	151.0403	2.81	C8H8O3	NEG	136.0166931;151.0401764;137.02005;92.02690125	75726833.13
Isovanillin	151.0403	2.81	C8H8O3	NEG	136.0166931;151.0401764;137.02005;92.02690125	75726833.13
Vanillin	151.0403	2.81	C8H8O3	NEG	136.0166931;151.0401764;137.02005;92.02690125	75726833.13
2-hydroxy-3-methoxy-benza	151.0403	2.81	C8H8O	NEG	136.0166931;151.0401764;137.02005;92.026901	75726833.

Compound	mz	rt/min	FORMULA	type	MS2 ion fragment	Responsesignal
ldehyde			3		25	13
2,5-Dihydroxyacetophenone	151.0403	2.81	C8H8O3	NEG	136.0166931;151.0401764;137.02005;92.02690125	75726833.13
4-Methylsalicylic acid	151.0403	2.81	C8H8O3	NEG	136.0166931;151.0401764;137.02005;92.02690125	75726833.13
Coniferol	179.0715	2.81	C10H12O3	NEG	146.0374603;164.0480042;179.0716858;161.0608215;59.01389694;135.0452728;87.00888824;71.01392365;147.0415192;144.8717651;165.0516205;143.8651276;89.02458954;118.9935074;178.0879974;163.0399475;151.0401306;145.0297699;101.0245972;178.0509186	8304461.696
3-(4-Methoxyphenyl)propanoic acid	179.0715	2.81	C10H12O3	NEG	146.0374603;164.0480042;179.0716858;161.0608215;59.01389694;135.0452728;87.00888824;71.01392365;147.0415192;144.8717651;165.0516205;143.8651276;89.02458954;118.9935074;178.0879974;163.0399475;151.0401306;145.0297699;101.0245972;178.0509186	8304461.696
3-(3-Methoxyphenyl)propanoic acid	179.0715	2.81	C10H12O3	NEG	146.0374603;164.0480042;179.0716858;161.0608215;59.01389694;135.0452728;87.00888824;71.01392365;147.0415192;144.8717651;165.0516205;143.8651276;89.02458954;118.9935074;178.0879974;163.0399475;151.0401306;145.0297699;101.0245972;178.0509186	8304461.696
3-(2-Methoxyphenyl)propanoic acid	179.0715	2.81	C10H12O3	NEG	146.0374603;164.0480042;179.0716858;161.0608215;59.01389694;135.0452728;87.00888824;71.01392365;147.0415192;144.8717651;165.0516205;143.8651276;89.02458954;118.9935074;178.0879974;163.0399475;151.0401306;145.0297699;101.0245972;178.0509186	8304461.696
2-(1-carboxyethyl)-5-methyl-cyclopentanecarboxylic acid	199.0977	2.83	C10H16O4	NEG	199.0977173;155.1078491;162.8392334;164.8362122;137.0971985;181.0871429;83.05023193;59.01393509;163.8399658;156.1108551;57.03462219;125.8735657;156.894989;127.0400314;73.02989197;161.8430176;125.0973892;198.1219177;111.0813675;127.1124268;171.1028137;100.9338379;109.0659027;135.0821686;71.05023956;198.1503754;138.1006927;99.04451752;138.4012756;69.03466034;160.6610413;135.9211731;113.024826;64.24098969;110.3273621	4102348.363
4-(3,4-dihydroxyphenyl)-7-hydroxy-5-[(2S,3R,4S,5S,6R)-3,4,5-trihydroxy-6-(hydroxymethyl)tetrahydropyran-2-yl]oxy-chromen-2-one	447.0939	2.86	C21H20O11	NEG	327.051239;357.0618286;447.0937805;328.0549622;299.0557861;297.0404358;285.0408325;358.0653992;369.0622864;298.0479431;339.0508118;284.0324402;59.01353073;325.0343628;393.0606384;429.0837097	6013342.579
2-(3,4-dihydroxyphenyl)-5,7-dihydroxy-6-[3,4,5-trihydroxy-6-(hydroxymethyl)tetrahydropyran-2-yl]chromen-4-one	447.0939	2.86	C21H20O11	NEG	327.051239;357.0618286;447.0937805;328.0549622;299.0557861;297.0404358;285.0408325;358.0653992;369.0622864;298.0479431;339.0508118;284.0324402;59.01353073;325.0343628;393.0606384;429.0837097	6013342.579
Isoorientin	447.0939	2.86	C21H20O11	NEG	327.051239;357.0618286;447.0937805;328.0549622;299.0557861;297.0404358;285.0408325;358.0653992;369.0622864;298.0479431;339.0508118;284.0324402;59.01353073;325.0343628;393.0606384;429.0837097	6013342.579
2-(1,5,8-trihydroxy-4a,8-dimethyl-1,2,3,4,5,6,7,8a-octahydronaphthalen-2-yl)prop-2-enoic acid	283.1553	2.86	C15H24O5	NEG	282.2520447;59.01379776;283.1566772	6741795.683
3-Hydroxyacetophenone	135.0454	2.87	C8H8O2	NEG	135.0452118;134.9941254;93.03469086;134.0476227;120.0216827;92.02675629;91.0221405;107.05056;108.0218658;106.0425797	8694676.953
Phenylacetic acid	135.0454	2.87	C8H8O2	NEG	135.0452118;134.9941254;93.03469086;134.0476227;120.0216827;92.02675629;91.0221405;107.05056;108.0218658;106.0425797	8694676.953
4-Hydroxyphenylacetaldehyde	135.0454	2.87	C8H8O2	NEG	135.0452118;134.9941254;93.03469086;134.0476227;120.0216827;92.02675629;91.0221405;107.05056;108.0218658;106.0425797	8694676.953
Hydroxytyrosol acetate	195.0665	2.88	C10H12O4	NEG	195.0640259;160.8422089;167.0692139;169.0846252;127.0765152;180.0437775;165.0552826;150.0321655;158.8469238;59.01385117;151.0759583;136.0530701;135.040863	8113290.327
Hydroferulic acid	195.0665	2.88	C10H12O4	NEG	195.0640259;160.8422089;167.0692139;169.0846252;127.0765152;180.0437775;165.0552826;150.0321655;158.8469238;59.01385117;151.0759583;136.0530701;135.040863	8113290.327
2,5-dimethylbenzene-1,3-diol	137.061	2.90	C8H10O2	NEG	134.9940948;137.0608978;135.0453339;119.0503845;137.0245361;93.0346756;107.0503845;135.9936676;136.0496521;120.9784546;108.021789	1443468.872

Compound	mz	rt/min	FORMULA	type	MS2 ion fragment	Responsesignal
					6;136.0149689;91.0218811;122.0382614;109.0293961;94.03797913;81.03458405;60.97530746;74.9912796	
4,5-dimethylbenzene-1,3-diol	137.061	2.90	C8H10O2	NEG	134.9940948;137.0608978;135.0453339;119.0503845;137.0245361;93.0346756;107.0503845;135.9936676;136.0496521;120.9784546;108.0217896;136.0149689;91.0218811;122.0382614;109.0293961;94.03797913;81.03458405;60.97530746;74.9912796	1443468.872
4-Ethylresorcinol	137.061	2.90	C8H10O2	NEG	134.9940948;137.0608978;135.0453339;119.0503845;137.0245361;93.0346756;107.0503845;135.9936676;136.0496521;120.9784546;108.0217896;136.0149689;91.0218811;122.0382614;109.0293961;94.03797913;81.03458405;60.97530746;74.9912796	1443468.872
3-(2-Hydroxyphenyl)propanoic acid	165.0559	2.90	C9H10O3	NEG	93.03465271;121.0660553;165.0557709;164.8365326;95.05032349;147.0451813;164.0710449;164.1078644;59.01389694;94.03791046;72.00919342;122.069313;150.0324554;109.0658798;106.0425873;151.0034027;148.0483246;137.0613251;136.0533295;119.0507507	10886323.18
6,8-dihydroxy-3-methyl-isochromen-1-one	191.0352	2.90	C10H8O4	NEG	191.0351715;176.0115967;111.0088654;87.00881195;85.02948761;163.0402832;59.01398849;91.05549622;177.0157776;148.0173492;147.0455933;129.0193787;145.8641815;144.8666992;61.98851776;57.03482437;119.0507431;93.03457642;135.0446472;145.9412384;120.0219193;190.0526886;121.0294647;89.083992;76.01516724;84.47278595	5719027.256
(4E)-8-hydroxy-4-(2-hydroxy-1-methyl-ethylidene)-10-oxatricyclo[7.2.1.01,5]dodecane-8-carboxylic acid	281.1398	2.90	C15H22O5	NEG	280.2363586;281.1431885;59.01387024;85.75856781;274.0559692;88.19199371	4249462.184
8-hydroxy-4-(1-hydroxy-1-methyl-ethyl)-10-oxatricyclo[7.2.1.01,5]dodec-3-ene-8-carboxylic acid	281.1398	2.90	C15H22O5	NEG	280.2363586;281.1431885;59.01387024;85.75856781;274.0559692;88.19199371	4249462.184
5-[6-(hydroxymethyl)-7-oxatricyclo[4.3.0.03,9]nonan-9-yl]-2-methyl-4-oxo-pentanoic acid	281.1398	2.90	C15H22O5	NEG	280.2363586;281.1431885;59.01387024;85.75856781;274.0559692;88.19199371	4249462.184
Cyclopropylacetic acid	99.0453	2.92	C5H8O2	NEG	99.04529572;99.0089035;98.02497864;55.01895142;71.01399231;43.01901245;41.00341415;59.01391602;81.0344696;41.99861908;52.930336;49.62852478;52.13112259;69.81878662;48.70893097;53.58376312;42.86409378;94.69765472	6342414.084
(2R)-7-hydroxy-8-(2-hydroxyethyl)-5-methoxy-2-methyl-chroman-4-one	251.0928	2.94	C13H16O5	NEG	71.01393127;251.0925293;137.0245667;141.0561066;99.0454483;109.029541;250.1454468;223.1707458;113.0608368;72.0173645;207.1024017;59.0139389;195.139679;233.1555328;236.067688	709646.9841
Salicylic acid	137.0246	2.96	C7H6O3	NEG	137.0245209;93.03468323;134.9941559;135.0456696;108.0218887;81.03479767;119.0502777;109.0296783;107.05056;136.0167084;136.0493469;94.03800964;122.0371704;95.0500412;135.9939423;109.065979;94.03022003;136.0382996;41.99853897;136.0764618;92.05066681;108.0453339;75.83859253	7520663.608
N-Acetyltryptophan	245.0934	2.98	C13H14N2O3	NEG	203.0825958;245.0936584;74.02479553;116.0354156;116.0505676;98.02487946;58.02986526;72.04562378;78.95909119;201.1091461;139.1128235;96.95989227;204.0848236;209.1177216;159.09198;183.1022339;59.01371002;142.0660248;70.02986908;130.065979;96.96856689;203.9728241;227.0929871;244.1546936;186.0550842;99.77420044;51.27581024;51.59308624	4891967.555
4,7-dihydroxychromen-2-one	177.0194	2.99	C9H6O4	NEG	177.0195007;149.0245056;141.8683624;121.0297623;162.0325165;123.0450134;93.03459167;176.0352478;133.0297089;95.05043793;163.0278015;77.03986359;176.0897064;112.0854645;134.0374756;105.0347366;59.01401138;176.0721436;129.0196838;114.0894852;117.0349274;57.03465271;71.01409149;159.8793488;99.00875854;160.8418579;91.05568695;43.1808815;43.10702515	4299431.324
methyl (E)-3-(3-hydroxy-4-methoxy-phenyl)prop-2-enoate	207.0664	2.99	C11H12O4	NEG	192.0428467;177.0193787;207.0662994;193.0462036;178.0227661;191.0354004;149.0242615	42445249.24

Compound	mz	rt/min	FORMULA	type	MS2 ion fragment	Responsesignal
3,5-Dimethoxycinnamic acid	207.0664	2.99	C11H12O4	NEG	192.0428467;177.0193787;207.0662994;193.0462036;178.0227661;191.0354004;149.0242615	42445249.24
2-acetylbenzoic acid	163.0403	3.00	C9H8O3	NEG	119.0503387;162.8392639;163.0402222;147.0450592;59.01383591;120.0540466;134.0371704;85.02954865;91.05529022;72.00914764;101.0246887;135.0817413;135.0455933;73.02960968	6525651.502
4-Hydroxycinnamic acid	163.0403	3.00	C9H8O3	NEG	119.0503387;162.8392639;163.0402222;147.0450592;59.01383591;120.0540466;134.0371704;85.02954865;91.05529022;72.00914764;101.0246887;135.0817413;135.0455933;73.02960968	6525651.502
3-Hydroxycinnamic acid	163.0403	3.00	C9H8O3	NEG	119.0503387;162.8392639;163.0402222;147.0450592;59.01383591;120.0540466;134.0371704;85.02954865;91.05529022;72.00914764;101.0246887;135.0817413;135.0455933;73.02960968	6525651.502
5-Hydroxyvalproic acid	159.1029	3.03	C8H16O3	NEG	159.1027679;113.0973892;59.0138588;73.0295639;114.1006165;101.9337463;85.02954865;141.0922089;131.0355072	59600734.68
8-hydroxy-3-methyl-isochroman-1-one	177.0558	3.06	C10H10O3	NEG	162.0323181;177.0557556;163.0357666;134.037384	72272693.62
2-Oxo-4-phenylbutyric acid	177.0558	3.06	C10H10O3	NEG	162.0323181;177.0557556;163.0357666;134.037384	72272693.62
4-Chromanone	147.0453	3.08	C9H8O2	NEG	147.0452728;41.00338745;59.01388931;119.0504379	1161285.377
(1R,4S,8R,9R,12S,13S,16S,19R)-9,19-dihydroxy-7,7-dimethyl-17-methylene-3,10-dioxapentacyclo[14.2.1.01,13.04,12.08,12]nonadecane-2,18-dione	361.1658	3.11	C20H26O6	NEG	361.1658325;165.0557556;346.142395;122.037468;179.0714569;121.0296173;136.0531769;315.1238403;109.0295944;347.1459045;100.9336319;116.9286118;279.2328796;223.0974426;313.1460571;298.12146;316.1300049;166.0595245;145.0506134;135.0458984;159.0660553	12969327.37
2,3-bis[(4-hydroxy-3-methoxy-phenyl)methyl]butane-1,4-diol	361.1658	3.11	C20H26O6	NEG	361.1658325;165.0557556;346.142395;122.037468;179.0714569;121.0296173;136.0531769;315.1238403;109.0295944;347.1459045;100.9336319;116.9286118;279.2328796;223.0974426;313.1460571;298.12146;316.1300049;166.0595245;145.0506134;135.0458984;159.0660553	12969327.37
4-[[(3R,4R,5S)-5-(4-hydroxy-3-methoxy-phenyl)-4-(hydroxymethyl)tetrahydrofuran-3-yl]methyl]-2-methoxy-phenol	359.1505	3.12	C20H24O6	NEG	329.1400452;277.2171326;175.0763702;178.0632935;160.0532532;192.0789795;96.96958923;330.1430359;359.1147156;344.086853;59.0138855;75.00875092;187.0977173;261.1351318;193.0854797;174.068985;209.0800323;314.0440674;278.2230835;116.9289474;136.0529633;161.0597382;193.0498047;301.0006714;208.0750427;205.0869751;173.0603485;190.0638733;159.0451202;191.0717926;177.0545654;125.0977859;222.0911255;100.1984482;279.0310059;76.43462372	6134588.857
Valeric acid	101.0609	3.12	C5H10O2	NEG	101.0608749;73.0295639;59.0138588;57.03463364	50329530.33
Isovaleric acid	101.0609	3.12	C5H10O2	NEG	101.0608749;73.0295639;59.0138588;57.03463364	50329530.33
3-Methoxyphenylacetic acid	165.0558	3.13	C9H10O3	NEG	164.8364258;165.0558167;93.03466797;147.0453339;150.0323486;164.0717621;121.0661163;121.0297241;120.0540924;72.00919342;119.0501099;103.0553894;148.0488434;137.0607452;109.0658569;122.0610733;75.00907135;59.0139122;109.0293961;106.0424118;149.0101929;135.0450592;108.0219116;95.04984283;137.0245514;123.044548;136.0521545;45.10717773;91.05513;45.49184418;151.0044098;108.4558716;101.1839294;94.03799438;107.0373154;136.0173645;50.70397568	4744872.674
4-Ethoxybenzoic acid	165.0558	3.13	C9H10O3	NEG	164.8364258;165.0558167;93.03466797;147.0453339;150.0323486;164.0717621;121.0661163;121.0297241;120.0540924;72.00919342;119.0501099;103.0553894;148.0488434;137.0607452;109.0658569;122.0610733;75.00907135;59.0139122;109.0293961;106.0424118;149.0101929;135.0450592;108.0219116;95.04984283;137.0245514;123.044548;136.0521545;45.10717773;91.05513;45.49184418;151.0044098;108.4558716;101.1839294;94.03799438;107.0373154;136.0173645;50.70397568	4744872.674
Ethyl 4-hydroxybenzoate	165.0558	3.13	C9H10O3	NEG	164.8364258;165.0558167;93.03466797;147.0453339;150.0323486;164.0717621;121.0661163;121.0297241;120.0540924;72.00919342;119.0501099;103.0553894;148.0488434;137.0607452;109.0658569;122.0610733;75.00907135;59.0139122;109.0293961;106.0424118;149.0101929;135.0450592;108.0219116;95.04984283;137.0245514;12	4744872.674

Compound	mz	rt/min	FORMULA	type	MS2 ion fragment	Responsesignal
					3.044548;136.0521545;45.10717773;91.05513;45.49184418;151.0044098;108.4558716;101.1839294;94.03799438;107.0373154;136.0173645;50.70397568	
Ethyl 3-hydroxybenzoate	165.0558	3.13	C9H10O3	NEG	164.8364258;165.0558167;93.03466797;147.0453339;150.0323486;164.0717621;121.0661163;121.0297241;120.0540924;72.00919342;119.0501099;103.0553894;148.0488434;137.0607452;109.0658569;122.0610733;75.00907135;59.0139122;109.0293961;106.0424118;149.0101929;135.0450592;108.0219116;95.04984283;137.0245514;123.044548;136.0521545;45.10717773;91.05513;45.49184418;151.0044098;108.4558716;101.1839294;94.03799438;107.0373154;136.0173645;50.70397568	4744872.674
Desaminotyrosine	165.0558	3.13	C9H10O3	NEG	164.8364258;165.0558167;93.03466797;147.0453339;150.0323486;164.0717621;121.0661163;121.0297241;120.0540924;72.00919342;119.0501099;103.0553894;148.0488434;137.0607452;109.0658569;122.0610733;75.00907135;59.0139122;109.0293961;106.0424118;149.0101929;135.0450592;108.0219116;95.04984283;137.0245514;123.044548;136.0521545;45.10717773;91.05513;45.49184418;151.0044098;108.4558716;101.1839294;94.03799438;107.0373154;136.0173645;50.70397568	4744872.674
Indole-3-carboxaldehyde	144.0457	3.14	C9H7NO	NEG	144.0455627;59.01390839;143.0714264	13150074.33
methyl (2S,3S,4S,5R,6S)-6-[5,6-dihydroxy-2-(4-hydroxyphenyl)-4-oxo-chromen-7-yl]oxy-3,4,5-trihydroxy-tetrahydropyran-2-carboxylate	475.0887	3.15	C22H20O12	NEG	299.0561218;113.0245132;284.0325623;85.0295639;300.0595703;59.01390839;175.024826;475.0882568;99.00878906;95.01379395;71.01391602;75.0087738;117.0194626;285.0363464;103.0036545;129.0199127;87.00880432;115.0034943;89.02437592	11167224.87
3-hydroxy-3,4-bis[(4-hydroxy-3-methoxy-phenyl)methyl]tetrahydrofuran-2-one	373.1296	3.17	C20H22O7	NEG	373.1293335;179.0713806;99.0087738;109.0296097;123.0452652;122.0373611;327.1238708;164.0480042;136.0529785;223.0615845;237.0769958;221.0453339;137.0607758;235.0613556;358.1065979;355.1191101;149.0608978;147.0454254;151.0764008;207.0662384;312.1004028;180.0749207;193.0870972;221.0815277;121.0293121;191.071579;148.0529633;311.1297607;176.0482178;177.0557404;340.0957947;55.01898575;249.0752106;315.1240234;190.0640106;178.063446;100.0122299;72.99299622;163.0404053;328.12677;163.0763245;296.1049805;206.057724;359.1072388;159.0449066;71.01392365;59.01376724;108.0216751	35131169.07
(2E,5E)-3,5,7-trimethylocta-2,5-dienedioic acid	211.0977	3.18	C11H16O4	NEG	211.097702;193.0869141;167.1077576;149.0973511;197.9866638;59.01389694;97.0295105;99.00896454;183.1390228;208.9768066;110.0376968;194.0896149;168.1105804;171;124.0405045;111.0819092;79.95737457;117.0463486;116.0510178;71.0140152;123.0813675;183.1032562;131.0865784;139.1131744;210.1225281;155.9888916;196.0389709;144.0572815;172.0075989;147.0818329;151.9165039;85.02937317;139.0768433	14962383.12
(2S,3S,4S,5R,6S)-6-[4-(5,7-dihydroxy-4-oxo-chromen-3-yl)phenoxy]-3,4,5-trihydroxy-tetrahydropyran-2-carboxylic acid	445.0781	3.20	C21H18O11	NEG	269.0455017;113.0244827;85.02951813;270.0487976;59.01387405;99.00878143;175.0247498;71.01384735;95.01392365;75.00873566;103.0037003;117.0194626;87.00879669;129.0194702;89.02458954;115.0038071;445.0771179;57.03460312	27258387.94
Baicalin	445.0781	3.20	C21H18O11	NEG	269.0455017;113.0244827;85.02951813;270.0487976;59.01387405;99.00878143;175.0247498;71.01384735;95.01392365;75.00873566;103.0037003;117.0194626;87.00879669;129.0194702;89.02458954;115.0038071;445.0771179;57.03460312	27258387.94
p-Toluquinone	121.0296	3.21	C7H6O2	NEG	121.0296707;77.03979492;94.02980042;61.98847198	22957801.29
Benzoic acid	121.0296	3.21	C7H6O2	NEG	121.0296707;77.03979492;94.02980042;61.98847198	22957801.29
Tropolone	121.0296	3.21	C7H6O2	NEG	121.0296707;77.03979492;94.02980042;61.98847198	22957801.29
2-(2-Butoxyethoxy)acetic acid	175.0977	3.22	C8H16O4	NEG	175.0976257;75.00882721;56.99824905;72.99317932;129.0922394;44.99827194;47.01387787;174.0849152;112.0850372	17658474.3

Compound	mz	rt/min	FORMULA	type	MS2 ion fragment	Responses ignal
(E)-2-methylpent-2-enoic acid	113.0609	3.26	C6H10 O2	NEG	113.0609894;85.02957916;57.03463364;59.01391983;68.9957962;69.034729;55.01899338;44.99818039;84.02184296;41.99857712;71.01396942;41.0033226;83.01387787;43.01891708	19116163.55
Azelaic acid	187.0975	3.28	C9H16 O4	NEG	125.0972824;187.0976257;169.0870819;97.06597137;143.1083679;123.0817184;126.1007385;57.03469086	2586699626
(E)-9,10-dihydroxytetradec-6-enedioic acid	269.1396	3.30	C14H24 O6	NEG	269.1395264;223.1339111;225.149765;71.01387787;251.1654816;269.0455627;251.1291656;227.128891;225.1131287;211.1340332;241.1440582;197.1183014;224.13797;59.0139389;209.1183014;195.139801;268.161499;125.0971985;233.1174622;207.1384735;193.1238251;233.1553345;252.166748;125.8731079;61.98859024;207.1763306;250.1520691;163.1123962;181.1233521;252.1338043;171.1030426;226.1522217;148.0162201;228.1330566;83.05039978;57.03458023;207.1036072;185.1181488;213.1503906;81.03453827;254.0604553;97.02901459;189.1637421;268.2377014;111.0817642;198.1216125;97.06565857;205.1230621;87.0453949;131.4740295;115.7579269;229.8749084;50.70356369	2730634.002
(2R,3S,4S,5R,6R)-2-(hydroxymethyl)-6-(5-isopropenyl-2-methyl-cyclohex-2-en-1-yl)oxy-tetrahydropyran-3,4,5-triol	295.1552	3.33	C16H26 O6	NEG	277.1446838;295.1555176;251.1291962;259.1343079;223.134079;221.1184235;249.1496735;239.1296082;233.120224;189.1282959;233.1528473;278.1461487;59.01393509;195.1394653;171.1033173;231.1385651;215.144104;136.05336;252.1309662;207.1393433;71.01399231;294.179718;109.0664368;280.0732422;134.0380096;260.1360779;235.134903;161.097229;95.05006409;205.15979;250.1522675;179.1445007;135.0820313;125.0976105;205.1222839;294.2174377;224.1374817;277.7619019;237.1149902;97.06577301;97.0296936;83.0502243;114.0671997;101.0241852;59.36223602;63.90166855;145.5738678	4166338.55
(2R,3R,4S,5S,6R)-2-[(2E)-2,5-dimethyl-4-vinyl-hexa-2,5-dienoxy]-6-(hydroxymethyl)tetrahydropyran-3,4,5-triol	295.1552	3.33	C16H26 O6	NEG	277.1446838;295.1555176;251.1291962;259.1343079;223.134079;221.1184235;249.1496735;239.1296082;233.120224;189.1282959;233.1528473;278.1461487;59.01393509;195.1394653;171.1033173;231.1385651;215.144104;136.05336;252.1309662;207.1393433;71.01399231;294.179718;109.0664368;280.0732422;134.0380096;260.1360779;235.134903;161.097229;95.05006409;205.15979;250.1522675;179.1445007;135.0820313;125.0976105;205.1222839;294.2174377;224.1374817;277.7619019;237.1149902;97.06577301;97.0296936;83.0502243;114.0671997;101.0241852;59.36223602;63.90166855;145.5738678	4166338.55
Butyllactate	145.0871	3.33	C7H14 O3	NEG	145.0871429;99.08164215;127.0767212;100.0849457;143.0714417	138346083.5
8-acetoxy-10-hydroxy-19-methoxy-1,9,18-trimethyl-15-oxo-16,20-dioxahexacyclo[15.3.2.02,13.04,12.07,11.014,19]docos-5-ene-5-carboxylic acid	503.2297	3.34	C27H36 O9	NEG	503.0232849;145.0871582;59.01393509;467.0602722;269.0455017;330.1099243;162.8937073;470.9991455;160.8419189;329.0679626;125.0973816;187.0984344;413.0519714;66.07299042;191.065918;140.6389008;116.9287415;92.43857574	14849352.81
Matairesinol	357.1344	3.34	C20H22 O6	NEG	357.1345825;83.01389313;137.0609131;342.1108398;221.0819855;122.0374832;123.0454254;121.0295258;136.0530701;209.0821075;147.0454559;313.1445007;162.0685425;298.1201172;343.1139832;151.0765686;161.0604095;84.0174408;55.01900864;206.0585785;191.0711823	1438392486
3-(2-hydroxyphenyl)-1-phenyl-prop-2-en-1-one	223.0744	3.36	C15H12 O2	NEG	179.1071014;223.0287933;176.8369293;164.835434;205.0871582;59.01402283;205.1239014;223.0667877;162.8397522;223.0575104;165.0921021;195.1388245;57.03481674;133.0400238;97.02970886;161.0980072;141.0924835;191.1145935;134.0478516;197.0039673;208.0391083;85.02906799;222.1207428;59.29081345;50.70702362;64.10745239;141.5693665;157.2324982;83.37810516;74.93685913;151.0769348;55.56411743	7540173.288
(E)-4-hydroxydodec-2-enedioic acid	225.1133	3.42	C12H20 O5	NEG	225.1130676;207.1025391;181.1233673;163.1128235;208.1061249;97.02946472;99.00865936;110.0373917;109.065979;182.1272888;137.0969849;197.1188354;59.01382446	129917821.7
Royal jelly acid	185.1184	3.42	C10H18 O3	NEG	185.1183167;141.0921936;141.1286469;125.0970612;57.03469467;116.0507507;139.1130981	208374575.4
(E)-8-hydroxy-2,6-dimethyl	185.1184	3.42	C10H18	NEG	185.1183167;141.0921936;141.1286469;125.097	208374575

Compound	mz	rt/min	FORMULA	type	MS2 ion fragment	Responsesignal
-oct-2-enoic acid			O3		0612;57.03469467;116.0507507;139.1130981	.4
Arctigenin	371.15	3.47	C21H24O6	NEG	83.01387787;371.1500549;136.0530396;151.0765381;356.1266479;235.0976563;121.0296936;137.0574646;84.01721191;55.01894379;357.1304932;312.1355591	642650606.8
Kaempferol	285.0405	3.48	C15H10O6	NEG	285.0404053;284.2677307;151.0039063;133.0296021;175.0401764;241.1456909	11433375.61
methyl 3,8-dihydroxy-9-oxo-xanthene-1-carboxylate	285.0405	3.48	C15H10O6	NEG	285.0404053;284.2677307;151.0039063;133.0296021;175.0401764;241.1456909	11433375.61
1,3,5,6-tetrahydroxy-2-methyl-anthracene-9,10-dione	285.0405	3.48	C15H10O6	NEG	285.0404053;284.2677307;151.0039063;133.0296021;175.0401764;241.1456909	11433375.61
3-Hydroxyoctanoic acid	159.1027	3.49	C8H16O3	NEG	59.01387787;159.1027374;60.01722336;101.9338379;73.02960968;100.9336395;85.02963257	81851905.95
3-Phenylpropanoic acid	149.0609	3.50	C9H10O2	NEG	149.060791;105.0710907;134.0374451;89.02459717;106.0422134	8687682.792
4-Allylcatechol	149.0609	3.50	C9H10O2	NEG	149.060791;105.0710907;134.0374451;89.02459717;106.0422134	8687682.792
4'-Hydroxy-3'-methylacetophenone	149.0609	3.50	C9H10O2	NEG	149.060791;105.0710907;134.0374451;89.02459717;106.0422134	8687682.792
4′-Hydroxy-2′-methylacetophenone	149.0609	3.50	C9H10O2	NEG	149.060791;105.0710907;134.0374451;89.02459717;106.0422134	8687682.792
2'-Hydroxy-4'-methylacetophenone	149.0609	3.50	C9H10O2	NEG	149.060791;105.0710907;134.0374451;89.02459717;106.0422134	8687682.792
Sebacic acid	201.1132	3.51	C10H18O4	NEG	201.1131897;139.112915;183.1026764;140.1162415;184.1060791;57.03461075;172.0769196;111.0816879;164.8361816;157.123642	296496373.6
5-[(1E,3E)-hepta-1,3-dienyl]-1,2,3-trihydroxy-cyclopentanecarboxylic acid	255.1238	3.52	C13H20O5	NEG	211.1341095;255.1223755;219.8451538;219.1392517;212.137619;237.1491699;120.0458145;185.1178284;237.1129913;193.1229858;254.1467896;197.1182861;57.03458023;138.176712;254.2193451;168.104126;237.2249298;110.1738052;144.0817566;75.58898163;54.34331512;109.6004105	8455274.429
1-(4-hydroxy-1,3-dihydroisobenzofuran-1-yl)butane-2,3-diol	223.0973	3.53	C12H16O4	NEG	223.0956573;195.1003113;179.1077271;155.1078033;59.01386261;205.0865631;197.116333;135.1183014;97.02960205;161.0966339;136.0530548;110.037384;222.1124725;177.08992;176.8367462	14435713.52
(1R,9S,10S)-3,4-dihydroxy-5-isopropyl-11,11-dimethyl-16-oxatetracyclo[7.5.2.01,10.02,7]hexadeca-2,4,6-triene-8,15-dione	343.1554	3.54	C20H24O5	NEG	343.1557617;328.1319275;187.0976105;329.1353455;125.0972137;122.0374985;263.1656494;201.1130524;185.1185608;313.1080627;207.1389008;93.070961;121.0294418;171.1022797;59.01384735;325.2064209;209.1181335;173.0822144;119.086937;289.1807861;342.2028198;191.0717773;139.0764771;155.0712891;111.0814743;121.0656738;163.0404663;109.0295639;327.1238708;307.1918335;183.0666962;299.1297913;123.0813065;157.0872498	10939343.73
10-Hydroxydecanoic acid	187.134	3.54	C10H20O3	NEG	125.0972672;187.0979614;187.1334076;97.0659256;126.1007156;169.0867462;143.1079102;141.1288757;123.0817184;57.03462982;186.1221466;141.8680878	12854476.45
Tropic acid	147.0453	3.54	C9H10O3	NEG	147.0451965;103.0554123;59.01390839;61.98846054;99.00854492;59.05021667;57.03446579;75.00881958;104.059082;85.02964783	5396642.386
Pinobanksin	271.0614	3.55	C15H12O5	NEG	271.0621033;241.0505981;256.0740356;151.0037384;119.0502853;253.144577;253.0507507;107.0139084;93.03466034;177.0192719;125.0973969;270.1788635;257.07724;270.2488098;181.1236267;242.0532074;125.0249557;199.1336517;143.0714111;213.0547943;83.01348114;165.0196838;197.0604858;111.0819244;209.1551514;59.01395798;270.2160339;97.06604767;83.05036926;227.0721893;255.0651855;157.0871124;152.0080872;243.1245575;226.15271;65.003479	9281247.598
6-hydroxy-1,5-dimethoxy-xanthen-9-one	271.0614	3.55	C15H12O5	NEG	271.0621033;241.0505981;256.0740356;151.0037384;119.0502853;253.144577;253.0507507;107.0139084;93.03466034;177.0192719;125.0973969;270.1788635;257.07724;270.2488098;181.1236267;242.0532074;125.0249557;199.1336517;143.0714111;213.0547943;83.01348114;165.0196838;197.0604858;111.0819244;209.1551514;59.01395798;270.2160339;97.06604767;83.05036926;227.0721893;255.0651855;157.0871124;152.0080872;243.1245575;226.15271;65.003479	9281247.598
(3Z,6S)-3-[[2-(1,1-dimethylallyl)-1H-indol-3-yl]methyl	322.1563	3.57	C19H21N3O2	NEG	305.1758423;322.1562195;185.1182709;251.1287994;223.1338654;287.1652527;137.060791;253	2396484.261

Compound	mz	rt/min	FORMULA	type	MS2 ion fragment	Responsesignal
ene]-6-methyl-piperazine-2,5-dione					.0847168;135.0816498;252.0780029;254.0885315;306.1789246;221.1180573;197.1181793;125.0973434;243.1749878;249.1495361;189.1283264;279.1593323;125.0608597;71.05023193;247.1330719;233.1169434;151.0764313;171.1021271;107.0503845;239.1286926;149.0973511;186.1215973;252.1302185;109.0665512;277.1807251;263.1645813;155.0611267;288.1687012;224.1376801;161.0600891;183.1024628;99.04524231;209.118042;237.1491089;269.1550598;203.1440582;123.0814896;213.113327;95.05005646;133.0658417;175.1130066;59.0138588;235.134613;184.1127777;138.064682;279.197113;195.1011963;265.1427002;207.138382;83.05042267;231.1390839;199.1340637	
Isocaproic acid	115.0765	3.59	C6H12O2	NEG	115.0765991	1035646460
Caproic acid	115.0765	3.59	C6H12O2	NEG	115.0765991	1035646460
Cyclopiazonic acid	335.1403	3.59	C20H20N2O3	NEG	335.1404419;148.0530396;320.1169739;178.0510559;149.0607605;190.0509338;191.058548;175.0401611;159.0928345;135.0451813;134.0373077;156.069397;321.1203308;192.0664978;176.0346832;177.0421906;276.103302;179.054306;150.0643158;160.0160217;164.8360901	16321695.26
4-Vinylphenol	119.0503	3.60	C8H8O	NEG	119.0504532;59.01387787;61.98838425;117.9289322	3582499.909
3-hydroxy-2-[(Z)-oct-2-enyl]pentanedioic acid	239.1289	3.60	C13H22O5	NEG	239.1286163;85.02954865;221.1183014;195.1028748;195.1382446;83.05029297;153.0922699;177.12854;171.1027069;57.03460693;193.1231537;155.1078186;83.0140152;222.1220856;110.0377045;196.1061096;211.133255;111.0817947;109.0662766;84.0218811;59.01381302;97.0292511;151.1133575;179.1444092;81.03460693;203.107132;95.05016327;69.034729;86.03310394;196.142334;125.0974121;67.01908112	33188177.72
(Z)-2-oct-7-enylpent-2-enedioic acid	239.1289	3.60	C13H20O4	NEG	239.1286163;85.02954865;221.1183014;195.1028748;195.1382446;83.05029297;153.0922699;177.12854;171.1027069;57.03460693;193.1231537;155.1078186;83.0140152;222.1220856;110.0377045;196.1061096;211.133255;111.0817947;109.0662766;84.0218811;59.01381302;97.0292511;151.1133575;179.1444092;81.03460693;203.107132;95.05016327;69.034729;86.03310394;196.142334;125.0974121;67.01908112	33188177.72
Cyclohexanecarboxylic acid	127.0765	3.63	C7H12O2	NEG	127.0766296;59.01390457;109.0660706	239539660.5
(2E)-2-hexylidene-3-methyl-butanedioic acid	213.1133	3.64	C11H18O4	NEG	169.1234436;213.113327;151.112915;170.1268311;195.1023865	60298174.86
2-hexyl-3-methylene-butanedioic acid	213.1133	3.64	C11H18O4	NEG	169.1234436;213.113327;151.112915;170.1268311;195.1023865	60298174.86
7,3',4'-Trihydroxyflavone	269.0456	3.64	C15H10O5	NEG	269.0455322;151.0038757;149.0245056;225.056076;117.0346909	130864374.3
Sulfuretin	269.0456	3.64	C15H10O5	NEG	269.0455322;151.0038757;149.0245056;225.056076;117.0346909	130864374.3
6,7,4'-Trihydroxyisoflavone	269.0456	3.64	C15H10O5	NEG	269.0455322;151.0038757;149.0245056;225.056076;117.0346909	130864374.3
2-Hydroxyoctanoic acid	159.1028	3.69	C8H16O3	NEG	100.9337234;159.1027832;113.0973663;59.01388168;101.9338913;115.9207687;116.9290161;73.02967834;97.06593323;129.0192413;158.0829773;141.0926208;114.1009064	13058233.11
Zearalanone	319.1554	3.71	C18H24O5	NEG	319.1558533;163.0765381;301.1446838;111.0816422;277.1446838;149.0610199;107.0503845;263.129303;247.0976868;145.0662384;106.0423584;197.1185913;135.0454865;121.0656815;257.1540222;209.1184845;283.133606;109.0659561;125.097435;259.1345215;164.0803528;207.1020203;249.1139374;275.1659241;83.05024719;275.12854;147.0446014;149.0964966;169.0871429;134.0373535;203.1087799;59.01381302;57.03459549;71.05033875;165.0919342;179.1080627;95.05051422;171.1023407;231.1021576;302.1488953;135.0816803;191.1077576;133.0293121;221.1186218;123.0814896;151.0769501;147.0812073;148.0526581;318.1773682;245.1180725;125.0610809;236.1058655;161.0975494;122.0375595;278.1480103;123.0452271;191.0349121;137.0606995;97.06623077;251.0692749;189.0558319;26	3486353.537

Compound	mz	rt/min	FORMULA	type	MS2 ion fragment	Responsesignal
[(5E)-3,4-dihydroxy-10-oxo-2-propyl-2,3,4,7,8,9-hexahydrooxecin-9-yl] (2E,4E)-hexa-2,4-dienoate	337.166	3.72	C18H26O6	NEG	1.1132507;127.0764542;237.1134338;291.1607056;192.0777893;215.1442261;127.0398788;93.03446198;190.063858;235.09729;110.0372162;192.0450287;233.1551819;193.0871735;276.1676941;176.0485382;175.1139679;243.102951;195.1044617;290.1170654;241.122406;112.0846939;177.1284943;235.1318054;189.091629;160.841156;175.0402374;318.2443237;205.0497437;199.1131744;179.0352478;185.0973206;217.1220551337.1654358;319.1547241;137.0608978;251.1643982;293.1765747;301.1451721;275.164917;125.0974274;167.0713196;251.1286774;213.1128845;129.0193634;127.0763092;171.1029968;154.0243378;199.0966187;59.01388931;237.1147919;295.153717;123.0452728;123.0812759;149.061554;109.0659943;237.146698;165.0556641;185.1178741;277.1431274;223.1335297;231.1746216;257.1534729;111.0450516;281.13797;221.1178741;183.1024628;71.05020142;97.02923584;263.1281433;201.1127167;135.0819855;99.04524231;291.1591797;152.0277557;85.02965546;153.0553131;225.1143494;71.01389313;199.1338654;151.0402985;97.06607819;121.0659409;233.1547546;95.05040741;320.1574402;263.1638184;109.0293808;252.1645203;149.0970154;269.0787964;73.02941895;205.1232758;107.0500031;57.03448105;155.1069336;139.0397034;153.0304108;249.1852875;128.080307;83.05043793;235.133316;177.1287689;209.1185303;127.040184;125.0607224;141.056076;336.1799316;283.1357117;194.058609;302.1469116;276.1676636;196.0976563;249.1491089;151.0762634;138.0927277;187.0969238;110.0694351;322.0820923;197.117691;175.1140594;309.1708374;93.03442383;233.1163788;111.0817719;166.0874023;265.1080627;139.0761871;255.2317963;296.1597595;126.1008835;101.0243073;153.0923004;247.1698608;279.1596985;239.127243;81.03485107;203.1078644;75.00926971;163.1128693;53.82291794;50.55126953;68.24414825	4818179.489
3-Phenylbutyric acid	163.0766	3.73	C10H12O2	NEG	163.0765686;91.05541229;59.01389694;119.0504379;119.0867157;92.05871582;71.01391602;147.0452118	22304098.64
4-Phenylbutanoic acid	163.0766	3.73	C10H12O2	NEG	163.0765686;91.05541229;59.01389694;119.0504379;119.0867157;92.05871582;71.01391602;147.0452118	22304098.64
4-Isopropylbenzoic acid	163.0766	3.73	C10H12O2	NEG	163.0765686;91.05541229;59.01389694;119.0504379;119.0867157;92.05871582;71.01391602;147.0452118	22304098.64
5,5-dimethyl-4-[(E)-3-methyl-7-[(2R,3R,4S,5S,6R)-3,4,5-trihydroxy-6-(hydroxymethyl)oxan-2-yl]oxyoct-3-enyl]oxolan-2-one	415.2341	3.73	C21H36O8	NEG	171.1026917;125.0972137;185.1182861;187.0975189;111.0816116;173.0818481;415.2348022;261.1343689;247.1186981;243.1237488;229.1081238;169.086792;59.01390457;172.1060638;279.1963501;297.2067871;157.0869446;126.1008072;201.113205;186.1221771;225.1135864;188.1013641;97.06594849;159.1024933;137.0971985;112.0849075;199.0974426;213.1131287;262.1383667;174.0848846;379.215271;155.1074066;83.05014801;183.1027985;141.0921783;57.03448486;397.2191162;199.1338806;139.0762939;61.98845291;257.1398621;414.2244873;396.2134094	13468374.93
2-hexylbutanedioic acid	201.1133	3.75	C10H18O4	NEG	201.1132813;157.1234436;139.1129913;183.1026611;200.1377411;164.8360138;74.02481842;125.0970688;158.1266785;59.01394653;159.8939667;155.1438904;127.1130676;166.832962;158.8933258;83.05021667;99.00896454;141.9364014;99.92590332;140.1166382;97.0659256;173.1184235;155.1078033;116.9285889;57.03462601;111.0814743;113.0970688;158.0613251;184.1063232	35409136.23
Monobutyl phthalate	221.0819	3.77	C12H14O4	NEG	71.05029297;69.03463745;221.0822144;177.0921631;121.0295639;134.0374298;165.0924225;149.0973511;147.0087738;177.1279144;133.1022339;59.01392365;203.1073151;77.03972626;193.122406;107.0503998;159.1187134;147.0819244;162.8395844;79.05550385;220.1398926;95.05044556;109.0407715;135.0444336;139.0765533;123.0817566;72.05353546;175.0765686;57.034721	10924833.4

Compound	mz	rt/min	FORMULA	type	MS2 ion fragment	Responsesignal
					37;105.0348892;178.1242371;178.096756;85.02945709;220.1061707;124.0404358;83.05010986;123.0452652	
Pregnenolone sulfate	377.1737	3.80	C21H32O5S	NEG	95.05032349;209.1177826;71.01390076;341.1980591;113.0610199;183.0669403;171.1031036;71.05031586;125.0972366;185.1179657;305.1775818;223.1332245;99.0453949;83.05024719;359.1862793;135.0822754;197.1186676;110.0378494;265.1448975;151.0767822;78.95889282;287.1656494;109.0664063;331.1924133;100.3454132;127.0403137;59.01390457;96.96920776;57.30519867;120.7782288;148.7724762;159.8271179;69.60073853	31698207.78
13,14-Dihydro-15-ketotetra norprostaglandin F1.beta.	299.1865	3.81	C16H28O5	NEG	299.1863708;125.0972214;281.1758423;237.1859589;284.0326233;111.0815582;255.1953888;282.1789246;97.06606293;298.2035828;169.0864868;139.1127167;126.1006927;183.1022491;127.0765381;59.01386642;173.0812378;285.0358276;263.1638794;157.0874939;187.0979614;171.1023407;171.0657959;238.1898193;129.0922089;298.2477417;83.05014801;185.1186218	23345841.21
2-[(3S,3aR,5R,7aS)-3a-acetyl-3-hydroxy-7a-methyl-2,3,4,5,6,7-hexahydro-1H-inden-5-yl]prop-2-enoic acid	265.1446	3.83	C15H22O4	NEG	265.1444702;223.1339722;247.1339722;203.1442108;237.1498108;95.05026245;203.1077423;205.1235504;177.1284943;185.1178589;221.1183929;221.1546631;224.1373596;248.1370392;264.16745;125.097374;229.1236877;149.09729;59.01392365;179.1438904;57.03463364;109.0658798;111.0452194;71.01383972;83.05020905;219.1395416;123.0817413;175.1130219;81.03490448;97.06604767;111.0818481;139.1127777;193.1603241	5149843.52
(1R,5R,8S)-2,3-dihydroxy-2,10,10-trimethyltricyclo[6.3.0.01,5]undec-6-ene-6-carboxylic acid	265.1446	3.83	C15H22O4	NEG	265.1444702;223.1339722;247.1339722;203.1442108;237.1498108;95.05026245;203.1077423;205.1235504;177.1284943;185.1178589;221.1183929;221.1546631;224.1373596;248.1370392;264.16745;125.097374;229.1236877;149.09729;59.01392365;179.1438904;57.03463364;109.0658798;111.0452194;71.01383972;83.05020905;219.1395416;123.0817413;175.1130219;81.03490448;97.06604767;111.0818481;139.1127777;193.1603241	5149843.52
(1S,5R,8S,9S)-7,9-dihydroxy-2,5,9-trimethyltricyclo[6.3.0.01,5]undec-2-ene-3-carboxylic acid	265.1446	3.83	C15H22O4	NEG	265.1444702;223.1339722;247.1339722;203.1442108;237.1498108;95.05026245;203.1077423;205.1235504;177.1284943;185.1178589;221.1183929;221.1546631;224.1373596;248.1370392;264.16745;125.097374;229.1236877;149.09729;59.01392365;179.1438904;57.03463364;109.0658798;111.0452194;71.01383972;83.05020905;219.1395416;123.0817413;175.1130219;81.03490448;97.06604767;111.0818481;139.1127777;193.1603241	5149843.52
(3aR,5S,7S,8R,8aS,9aR)-7,8-dihydroxy-5,8a-dimethyl-1-methylene-4,5,5a,6,7,8,9,9a-octahydro-3aH-azuleno[6,5-b]furan-2-one	265.1446	3.83	C15H22O4	NEG	265.1444702;223.1339722;247.1339722;203.1442108;237.1498108;95.05026245;203.1077423;205.1235504;177.1284943;185.1178589;221.1183929;221.1546631;224.1373596;248.1370392;264.16745;125.097374;229.1236877;149.09729;59.01392365;179.1438904;57.03463364;109.0658798;111.0452194;71.01383972;83.05020905;219.1395416;123.0817413;175.1130219;81.03490448;97.06604767;111.0818481;139.1127777;193.1603241	5149843.52
(1S,5S,8R,9S)-9-hydroxy-9-(hydroxymethyl)-2,5-dimethyltricyclo[6.3.0.01,5]undec-2-ene-3-carboxylic acid	265.1446	3.83	C15H22O4	NEG	265.1444702;223.1339722;247.1339722;203.1442108;237.1498108;95.05026245;203.1077423;205.1235504;177.1284943;185.1178589;221.1183929;221.1546631;224.1373596;248.1370392;264.16745;125.097374;229.1236877;149.09729;59.01392365;179.1438904;57.03463364;109.0658798;111.0452194;71.01383972;83.05020905;219.1395416;123.0817413;175.1130219;81.03490448;97.06604767;111.0818481;139.1127777;193.1603241	5149843.52
3a,8-dihydroxy-3,5a,9-trimethyl-4,5,6,7,8,9b-hexahydro-3H-benzo[g]benzofuran-2-one	265.1446	3.83	C15H22O4	NEG	265.1444702;223.1339722;247.1339722;203.1442108;237.1498108;95.05026245;203.1077423;205.1235504;177.1284943;185.1178589;221.1183929;221.1546631;224.1373596;248.1370392;264.16745;125.097374;229.1236877;149.09729;59.01392365;179.1438904;57.03463364;109.0658798;111.0452194;71.01383972;83.05020905;219.1	5149843.52

Compound	mz	rt/min	FORMULA	type	MS2 ion fragment	Responsesignal
					395416;123.0817413;175.1130219;81.03490448;97.06604767;111.0818481;139.1127777;193.1603241	
(Z)-9,12,13-trihydroxyoctadec-15-enoic acid	329.2334	3.84	C18H34O5	NEG	329.2334595;211.1338959;229.1443481;171.1026764;139.112854;99.08156586;183.1391296;311.2227783;212.1374207;293.2118835;127.1127167;201.1128082;193.122879;230.1477509;209.1184387;328.2209167	831163255.6
(Z)-5,8,11-trihydroxyoctadec-9-enoic acid	329.2334	3.84	C18H34O5	NEG	329.2334595;211.1338959;229.1443481;171.1026764;139.112854;99.08156586;183.1391296;311.2227783;212.1374207;293.2118835;127.1127167;201.1128082;193.122879;230.1477509;209.1184387;328.2209167	831163255.6
(E,9S,12S,13S)-9,12,13-trihydroxyoctadec-10-enoic acid	329.2334	3.84	C18H34O5	NEG	329.2334595;211.1338959;229.1443481;171.1026764;139.112854;99.08156586;183.1391296;311.2227783;212.1374207;293.2118835;127.1127167;201.1128082;193.122879;230.1477509;209.1184387;328.2209167	831163255.6
(E)-9,12,13-trihydroxyoctadec-10-enoic acid	329.2334	3.84	C18H34O5	NEG	329.2334595;211.1338959;229.1443481;171.1026764;139.112854;99.08156586;183.1391296;311.2227783;212.1374207;293.2118835;127.1127167;201.1128082;193.122879;230.1477509;209.1184387;328.2209167	831163255.6
(Z)-9,10,11-trihydroxyoctadec-12-enoic acid	329.2334	3.84	C18H34O5	NEG	329.2334595;211.1338959;229.1443481;171.1026764;139.112854;99.08156586;183.1391296;311.2227783;212.1374207;293.2118835;127.1127167;201.1128082;193.122879;230.1477509;209.1184387;328.2209167	831163255.6
(1S,5S,8R,9S)-9-hydroxy-2,5,9-trimethyltricyclo[6.3.0.01,5]undec-2-ene-3-carboxylic acid	249.1496	3.85	C15H22O3	NEG	249.1494751;203.1440582;187.1490784;205.1599731;231.1387787;85.02939606;248.166275	39924412.93
2-(1-hydroxypropan-2-yl)-3,3,6-trimethyl-5,6-dihydro-2H-1-benzofuran-7-carbaldehyde	249.1496	3.85	C15H22O3	NEG	249.1494751;203.1440582;187.1490784;205.1599731;231.1387787;85.02939606;248.166275	39924412.93
Dodecanedioic acid	229.1445	3.89	C12H22O4	NEG	229.1443481;211.1338959;167.144104;212.1371307;168.1475677;185.155365	41639937.26
2-Methylcaproic acid	129.0921	3.90	C7H14O2	NEG	129.0922241	710463024.6
8a-Hydroxy-4a,8-dimethyl-3,4,5,6,7,8-hexahydronaphthalene-2-carboxylic acid	223.1339	3.90	C13H20O3	NEG	223.0284271;223.1338806;195.1390228;205.1234894;179.1440887;167.1077423;59.01386642;179.1084595;69.03460693;141.0922089;74.9908371;149.0977478;71.05010986;177.1288605;196.1425476;57.03462601	339041789
4-hydroxy-4-[(E)-3-hydroxybut-1-enyl]-3,5,5-trimethyl-cyclohex-2-en-1-one	223.1339	3.90	C13H20O3	NEG	223.0284271;223.1338806;195.1390228;205.1234894;179.1440887;167.1077423;59.01386642;179.1084595;69.03460693;141.0922089;74.9908371;149.0977478;71.05010986;177.1288605;196.1425476;57.03462601	339041789
5-(2-hydroxyethyl)-3,5a-dimethyl-3a,4,4a,5,6,6a-hexahydro-3H-cyclopropa[f][1]benzofuran-2-one	223.1339	3.90	C13H20O3	NEG	223.0284271;223.1338806;195.1390228;205.1234894;179.1440887;167.1077423;59.01386642;179.1084595;69.03460693;141.0922089;74.9908371;149.0977478;71.05010986;177.1288605;196.1425476;57.03462601	339041789
4-(4-methoxyphenyl)butan-2-one	177.0921	3.91	C11H14O2	NEG	177.0921173;159.0815887;105.0710602;59.01386261	48534694.78
1,3,8-trihydroxy-6-(2-hydroxypropyl)anthracene-9,10-dione	313.0718	3.92	C17H14O6	NEG	253.0505676;312.2990112;313.0722961;312.229187;295.1910706;251.2016449;125.0973511;271.0611267;254.053894;294.2156982;185.0813904;157.0866547;187.0975952;238.1882782;173.0814972;269.2112122;171.1030731;141.0918732;298.0470276;139.1131744;276.2044678;59.01380157;212.1367493;145.0871735;111.0818405;97.06581879;197.0609131;268.1995239;225.0557251	19566938.4
2,6-dihydroxy-7-methoxy-1,1,4a-trimethyl-3,4,10,10a-tetrahydro-2H-phenanthren-9-one	303.1601	3.92	C18H24O4	NEG	303.1600647;285.1494751;259.1701355;241.1598358;122.037468;137.0608368;302.1764221;123.0453796;59.01378632;160.0531311;136.0529938;286.1522217;129.0918732;162.0681458;275.1656189;187.113266;148.0537567;135.0814056;260.1729126;147.0812073;205.123642;257.1542053;125.0608368;261.1496582;267.1383057;133.0660706;165.0915527;123.0810852;135.0455017;125.097023;177.091629;247.1335449;174.0687714;231.1750641;163.0752411;150.0689087	4927090.807
Sulfaphenazole	313.0718	3.92	C15H14N4O2S	NEG	253.0505676;312.2990112;313.0722961;312.229187;295.1910706;251.2016449;125.0973511;271	19566938.4

Compound	mz	rt/min	FORMULA	type	MS2 ion fragment	Responses ignal
					.0611267;254.053894;294.2156982;185.0813904;157.0866547;187.0975952;238.1882782;173.0814972;269.2112122;171.1030731;141.0918732;298.0470276;139.1131744;276.2044678;59.01380157;212.1367493;145.0871735;111.0818405;97.06581879;197.0609131;268.1995239;225.0557251	
(E)-1-(3,4-dihydroxy-2,6-dimethoxy-phenyl)-3-phenyl-prop-2-en-1-one	299.0925	3.92	C17H16O5	NEG	269.0454407;284.0683594;299.0927124;283.0611877;270.0488892;241.0503693;285.0717773;164.9829865;180.0065308;281.1758728;255.0658112	7431008.441
5,7-dihydroxy-2-phenyl-chroman-4-one	255.0661	3.93	C15H12O4	NEG	255.0662842;213.0556641;151.0037842;254.054306;211.0764313;145.0657806;219.845459;107.0138626;187.0764923;83.0137558;171.0454254	4470420.961
3-phenyl-1-(2,4,6-trihydroxyphenyl)prop-2-en-1-one	255.0661	3.93	C15H12O4	NEG	255.0662842;213.0556641;151.0037842;254.054306;211.0764313;145.0657806;219.845459;107.0138626;187.0764923;83.0137558;171.0454254	4470420.961
(2Z)-6-hydroxy-2-[(4-hydroxy-3-methoxy-phenyl)methylene]benzofuran-3-one	283.0611	3.94	C16H12O5	NEG	283.0612183;268.0377502;269.0411987;282.2523804	2550120715
(1aS,3aS,6aS,6bR)-2-formyl-5,5,6b-trimethyl-3a,4,6,6a-tetrahydro-1H-cyclopropa[e]indene-1a-carboxylic acid	247.134	3.95	C15H20O3	NEG	247.1339264;203.1440582;149.097229;59.01385498;205.1233368;175.1129761;121.0660324;229.1226807;204.1469574;109.0660629;95.05024719;123.0817413;245.1182251;108.0582352;150.1004181;97.06597137;219.139679;106.042511;231.1025543;161.1344147;83.05016327;201.1291046;161.0975647;107.0503922;206.1272278;131.0504456;174.1052094;187.1127319;81.03471375;147.0818024;185.1330566;135.0815277;162.1055603;111.0816956;185.0976563;145.0664978;213.0927277;120.0583572	3511511.467
(1S,2Z,10R)-11,11-dimethyl-7-methylidene-6-oxobicyclo[8.1.0]undec-2-ene-3-carboxylic acid	247.134	3.95	C15H20O3	NEG	247.1339264;203.1440582;149.097229;59.01385498;205.1233368;175.1129761;121.0660324;229.1226807;204.1469574;109.0660629;95.05024719;123.0817413;245.1182251;108.0582352;150.1004181;97.06597137;219.139679;106.042511;231.1025543;161.1344147;83.05016327;201.1291046;161.0975647;107.0503922;206.1272278;131.0504456;174.1052094;187.1127319;81.03471375;147.0818024;185.1330566;135.0815277;162.1055603;111.0816956;185.0976563;145.0664978;213.0927277;120.0583572	3511511.467
7,8-dihydroxy-4-phenyl-chromen-2-one	253.0507	3.96	C15H10O4	NEG	253.0505829;209.1547089;59.01390839;209.0608978;252.1690521	9647721.266
6-hydroxy-2-(2-hydroxyphenyl)chromen-4-one	253.0507	3.96	C15H10O4	NEG	253.0505829;209.1547089;59.01390839;209.0608978;252.1690521	9647721.266
2-(3,4-dihydroxyphenyl)chromen-4-one	253.0507	3.96	C15H10O4	NEG	253.0505829;209.1547089;59.01390839;209.0608978;252.1690521	9647721.266
Rubiadin	253.0507	3.96	C15H10O4	NEG	253.0505829;209.1547089;59.01390839;209.0608978;252.1690521	9647721.266
trans-2-Octenoic acid	141.0922	3.97	C8H14O2	NEG	141.0922394;59.0139122	97432225.55
3-hydroxy-2-octyl-pentanedioic acid	241.1445	4.01	C13H24O5	NEG	241.1444702;223.1339722;179.1441956;197.1547699;224.1372833;180.147522;57.03466034;71.05019379;198.1586914;125.0974121;195.1392059;225.1496277	43044570.56
[(2R,3R,4R,8S,10R,11R)-2,3,11-trihydroxy-4,6,6,10-tetramethyl-8-tricyclo[5.3.1.04,11]undecanyl] acetate	311.1867	4.02	C17H28O5	NEG	293.2122498;267.1966858;171.1026459;310.2108765;210.1215973;157.0871277;211.1344147;292.2000427;311.1843262;186.1217651;237.1864319;113.0975266;99.08164978;275.2018738;196.105835;209.1178284;195.1030426;185.1181183;172.10672;294.2169189;153.128952;183.1403351;100.0854187;114.1013565;311.1271973;111.0822067;125.0978546;57.03477097;181.1248932;97.06624603;268.2032776;249.2240295;139.1136169;83.05051422;127.1132507;183.1031342;199.1345367;155.1085205;167.1073761;249.1884613;169.1244049;291.1978149;109.0658798;225.1510162;195.1400757;59.01408386;121.1020279;137.0981903;149.0987244;98.21785736;100.9472427;81.03468323;197.108139;64.03484344;127.9130478;121.5388489;197.1203613;93.3263855	29221823.38
methyl 8-hydroxy-9-oxo-xanthene-1-carboxylate	269.0459	4.03	C15H10O5	NEG	269.0454407;268.0377197;151.0039825;241.0504456;251.165451;149.0245056;225.0557556;268.1626892;117.0348206;250.1525879	9156016.833
Emodin	269.0459	4.03	C15H10O5	NEG	269.0454407;268.0377197;151.0039825;241.0504456;251.165451;149.0245056;225.0557556;268	9156016.833

Compound	mz	rt/min	FORMULA	type	MS2 ion fragment	Responsesignal
					.1626892;117.0348206;250.1525879	
3,7-dihydroxy-9-methoxy-1-methyl-benzo[c]chromen-6-one	271.0613	4.04	C15H12O5	NEG	271.0610657;256.0376587;270.0493469;257.040863;270.210083	536104.5829
1,6-dihydroxy-3-methoxy-8-methyl-xanthen-9-one	271.0613	4.04	C15H12O5	NEG	271.0610657;256.0376587;270.0493469;257.040863;270.210083	536104.5829
7-methoxy-8-(3-methyl-5-oxo-2H-furan-4-yl)chromen-2-one	271.0613	4.04	C15H12O5	NEG	271.0610657;256.0376587;270.0493469;257.040863;270.210083	536104.5829
Aurantiamide acetate	443.198	4.06	C27H28N2O4	NEG	59.0138855;383.1766052;262.1236877;120.0455933;248.0956116;292.1216125;384.1808472;222.0927734;201.0667877	144379516.8
(3aS,5R,7S,7aR)-2,7-diacetyloxy-3-formyl-3a-hydroxy-1,1,3,5-tetramethyl-2,4,5,6,7,7a-hexahydroindene-4-carboxylic acid	383.1764	4.06	C19H28O8	NEG	383.1761475;120.0455856;248.0955353;262.1241455;292.1218872;201.0670013;222.0924683;249.1011963;353.1661987;247.0876312;265.098053;91.05536652;132.0820007;263.127594;295.2279053;339.2168884;195.0817108;293.1250305;326.1558838;250.0843506;125.0968933;291.1136169;116.0508423;321.2052917;291.1968079;309.207428;223.0950317;68.01431274;121.0493546;158.0617065;202.0698547;187.0983429;171.1027985;220.0771332;197.1182861;185.1183777;354.1686096;59.01388931;277.2180176;170.0621033;221.0967255;225.1133575;261.1031189;200.0595703;171.0696106;365.2343445;223.1347656	15311957.26
(1R,2R,4S,16R,17R,20S)-2-hydroxy-13,20-dimethoxy-4,7,17,22,22-pentamethyl-5,10,21,23-tetraoxahexacyclo[18.2.1.01,17.04,16.06,14.08,12]tricosa-6(14),7,12-trien-11-one	473.2184	4.12	C26H34O8	NEG	59.01388168;187.0969696;229.1435547;145.0868378;125.0972443;159.0664215;60.01731491	136651042
2-Methylheptanoic acid	143.1078	4.13	C8H16O2	NEG	143.1078186	587607342.3
Valproic acid	143.1078	4.13	C8H16O2	NEG	143.1078186	587607342.3
Caprylic acid	143.1078	4.13	C8H16O2	NEG	143.1078186	587607342.3
(1R,2R,6R,9R)-2,11,11-trimethyl-3-oxo-tricyclo[4.3.2.01,5]undecane-9-carboxylic acid	249.1496	4.13	C15H22O3	NEG	249.1495361;231.1390076;69.03464508;193.1234283;221.1547241;205.1597443;167.107666;59.01389694;205.1232605;149.1337891;187.1491394;248.1659851;232.1431885;95.05010223;83.05031586;248.1367188;194.1267548;161.1334991;97.06588745;179.1077728;151.1124878;203.1440277;206.1623077;222.157959;109.0662537;71.01390839;134.0737152;111.0816116	16110247.34
2-[(2S,4aR,8aS)-2-hydroxy-4a-methyl-8-methylene-decalin-2-yl]prop-2-enoic acid	249.1496	4.13	C15H22O3	NEG	249.1495361;231.1390076;69.03464508;193.1234283;221.1547241;205.1597443;167.107666;59.01389694;205.1232605;149.1337891;187.1491394;248.1659851;232.1431885;95.05010223;83.05031586;248.1367188;194.1267548;161.1334991;97.06588745;179.1077728;151.1124878;203.1440277;206.1623077;222.157959;109.0662537;71.01390839;134.0737152;111.0816116	16110247.34
(3S,3aR,8aS)-3,3a-dihydroxy-5-isopropylidene-3,8-dimethyl-1,2,4,8a-tetrahydroazulen-6-one	249.1496	4.13	C15H22O3	NEG	249.1495361;231.1390076;69.03464508;193.1234283;221.1547241;205.1597443;167.107666;59.01389694;205.1232605;149.1337891;187.1491394;248.1659851;232.1431885;95.05010223;83.05031586;248.1367188;194.1267548;161.1334991;97.06588745;179.1077728;151.1124878;203.1440277;206.1623077;222.157959;109.0662537;71.01390839;134.0737152;111.0816116	16110247.34
2-Hydroxydecanoic-acid	187.134	4.17	C10H20O3	NEG	125.0973282;187.0980988;187.1335907;141.1286469;97.06604767;169.0871277;126.1005859;57.03449631;123.0818405;143.1077423;142.1317139;169.1231232;59.01366425	19438174.07
8-hydroxy-6-methyl-2-(4-methylpent-3-enyl)octa-2,6-dienoic acid	251.1653	4.20	C15H24O3	NEG	251.1651306;71.05027008;125.097229;207.1756439;167.1075592;107.0504074;59.01377106;179.107605;195.1390381;233.15448;97.06602478	42813234.39
Dodecyl gallate	337.2023	4.21	C19H30O5	NEG	263.1653137;293.2124939;319.2289429;219.1758728;275.2011414;309.2074585;59.01383972;337.1989746;235.1707153;295.1924438;291.1964417;319.1899719;251.1655121;171.1026611;183.1391144;294.2170715;237.1500092;281.1747437;57.03461456;337.1073303;185.1186523;264.1697693;279.1968994;125.0973969;223.1706543;233.15448;71.05023193;97.02981567;199.13407	23549070.99

Compound	mz	rt/min	FORMULA	type	MS2 ion fragment	Responses ignal
					9;113.097641;318.2148743;336.2573547;99.0820694;320.2318115;221.1546631;211.1335449;245.1547546;305.176239;195.13974;97.06604004;165.1288757;336.2187195;137.0970459;273.18573;73.02962494;213.1481934;237.1847839;111.0819931;223.1351013;121.1024017;249.2225952;239.1631165;276.2062073;310.2113037;209.1179504;95.05027771;109.066246;149.1343689;207.1384735;87.04478455;183.104248;71.01396179;139.112793;181.1236115;287.1648865;83.05050659;151.1131439;191.2404938;153.0924072;125.0606308;167.1071167;85.06608582;141.1290436;115.0405197;59.37295914;197.1172791;157.0870361;72.79650879;100.9173355;137.0588531;145.2643585	
6,9a,11a-Trimethyl-4-oxo-1,2,3a,3b,5,5a,7,8,9,9b,10,11-dodecahydronaphtho[1,2-g][1]benzofuran-6-carboxylic acid	333.2072	4.23	C20H30O4	NEG	333.2078247;332.2529602;315.1987915;314.2411194;175.0979156;145.0871735;289.2181702;59.01396561;331.1921997;269.1909485;287.2018127	8919940.75
(1S,5R,9S,12S)-16-isopropyl-5,9-dimethyl-13,14-dioxatetracyclo[10.2.2.01,10.04,9]hexadec-15-ene-5-carboxylic acid	333.2072	4.23	C20H30O4	NEG	333.2078247;332.2529602;315.1987915;314.2411194;175.0979156;145.0871735;289.2181702;59.01396561;331.1921997;269.1909485;287.2018127	8919940.75
2-hydroxy-1',4,4'a-trimethyl-5'-oxo-4-vinyl-spiro[cyclopentane-1,6'-decalin]-1'-carboxylic acid	333.2072	4.23	C20H30O4	NEG	333.2078247;332.2529602;315.1987915;314.2411194;175.0979156;145.0871735;289.2181702;59.01396561;331.1921997;269.1909485;287.2018127	8919940.75
5-[(E)-4-carboxy-3-methyl-but-3-enyl]-1,4a-dimethyl-6-methylene-decalin-1-carboxylic acid	333.2072	4.23	C20H30O4	NEG	333.2078247;332.2529602;315.1987915;314.2411194;175.0979156;145.0871735;289.2181702;59.01396561;331.1921997;269.1909485;287.2018127	8919940.75
5,9-dimethyltetracyclo[11.2.1.01,10.04,9]hexadecane-5,14-dicarboxylic acid	333.2072	4.23	C20H30O4	NEG	333.2078247;332.2529602;315.1987915;314.2411194;175.0979156;145.0871735;289.2181702;59.01396561;331.1921997;269.1909485;287.2018127	8919940.75
(E)-2-(4-Methylpent-3-enyl)-6-oxohept-2-enoic acid	223.1339	4.23	C13H20O3	NEG	223.1341858;195.1390533;205.1233673;179.144165;167.1078186;59.01382065;69.03463745;179.1082458;164.8362122;176.8372498;196.1430969;71.05039215;57.03466797;149.0970306;125.0972672;177.1290436;95.0502243;109.0661163;85.06585693;108.0582428;206.1273804;161.1333923	6503592.712
(10Z,13Z)-15,16-dihydroxyoctadeca-10,13-dienoic acid	311.2228	4.24	C18H32O4	NEG	311.2229004;223.1702728;87.0451889;293.2125549;275.2016296;235.1703644;224.1738739;57.03459167;253.1810303;85.02939606;309.20755;183.1400146	1758328854
4-acetoxy-8-(3-oxo-2-pent-2-enyl-cyclopenten-1-yl)octanoic acid	349.1969	4.25	C20H30O5	NEG	311.223114;127.0404053;349.1976318;312.2278137;305.2121582;223.1708069;313.2367859;83.05029297;331.1893311;113.0245438;224.1742554;348.2227783;87.04538727;59.01397324;287.2035217;275.2007751;331.2328796;196.3167877;233.1205139;83.83285522;71.01407623;196.8869781;119.9210129;56.75397873	13943822.89
5-[(E)-4-carboxy-3-methyl-but-3-enyl]-3-hydroxy-1,4a-dimethyl-6-methylene-decalin-1-carboxylic acid	349.1969	4.25	C20H30O5	NEG	311.223114;127.0404053;349.1976318;312.2278137;305.2121582;223.1708069;313.2367859;83.05029297;331.1893311;113.0245438;224.1742554;348.2227783;87.04538727;59.01397324;287.2035217;275.2007751;331.2328796;196.3167877;233.1205139;83.83285522;71.01407623;196.8869781;119.9210129;56.75397873	13943822.89
4-[2-[(1R,4aS,5R,6R,8aS)-6-hydroxy-5-(hydroxymethyl)-5,8a-dimethyl-2-methylene-decalin-1-yl]-1-hydroxy-ethyl]-2H-furan-5-one	349.1969	4.25	C20H30O5	NEG	311.223114;127.0404053;349.1976318;312.2278137;305.2121582;223.1708069;313.2367859;83.05029297;331.1893311;113.0245438;224.1742554;348.2227783;87.04538727;59.01397324;287.2035217;275.2007751;331.2328796;196.3167877;233.1205139;83.83285522;71.01407623;196.8869781;119.9210129;56.75397873	13943822.89
5-[2-(3-furyl)ethyl]-8-hydroxy-5,6,8a-trimethyl-3,4,4a,6,7,8-hexahydronaphthalene-1-carboxylic acid	331.1916	4.26	C20H28O4	NEG	331.1919861;217.0873718;288.160614;330.2362366;287.2017517;275.2018433;313.1801758;293.2131958;59.01396179;235.1687927;86.52674103;201.1139984;57.64878464;50.96218872	31870929.14
Marrubiin	331.1916	4.26	C20H28O4	NEG	331.1919861;217.0873718;288.160614;330.2362366;287.2017517;275.2018433;313.1801758;293.2131958;59.01396179;235.1687927;86.52674103;201.1139984;57.64878464;50.96218872	31870929.14
2-[(2,6-dimethylphenyl)carb	268.098	4.27	C16H15	NEG	268.0977478;91.05539703;224.1082611;267.196	14061609.

Compound	mz	rt/min	FORMULA	type	MS2 ion fragment	Responsesignal
amoyl]benzoic acid			NO3		1975;120.0456085;268.0405273;147.0453644;267.1595459;132.0456085;225.1853485;249.1856842;223.1700592;133.053833;249.1496735;207.1755371;225.1111145;195.1387177;92.05861664;165.1281891;251.1651459;169.1226654;116.0716171;250.1887817;59.01399994;113.0973816;125.0971909;114.0923386;167.1090851;102.0353165;121.0489578;92.05060577;223.2065277;151.0043945;72.99317169;57.03471375;115.0757599;171.1023865;169.0867157;209.1196442;205.159256;197.0968323;128.0358276;237.1497803;250.1539612;75.00883484;87.3572998;154.0513458;141.0919647;149.024231;185.1178741;135.3562469;68.99851227;83.04997253;129.8156891;236.9730682;83.52458954;73.74687195;64.46956635	13
(2S)-2-benzamido-3-phenyl-propanoic acid	268.098	4.27	C16H15NO3	NEG	268.0977478;91.05539703;224.1082611;267.1961975;120.0456085;268.0405273;147.0453644;267.1595459;132.0456085;225.1853485;249.1856842;223.1700592;133.053833;249.1496735;207.1755371;225.1111145;195.1387177;92.05861664;165.1281891;251.1651459;169.1226654;116.0716171;250.1887817;59.01399994;113.0973816;125.0971909;114.0923386;167.1090851;102.0353165;121.0489578;92.05060577;223.2065277;151.0043945;72.99317169;57.03471375;115.0757599;171.1023865;169.0867157;209.1196442;205.159256;197.0968323;128.0358276;237.1497803;250.1539612;75.00883484;87.3572998;154.0513458;141.0919647;149.024231;185.1178741;135.3562469;68.99851227;83.04997253;129.8156891;236.9730682;83.52458954;73.74687195;64.46956635	14061609.13
(10R,11R)-10,11-dihydroxy-9-(hydroxymethyl)-1,6a,6b,9,12a-pentamethyl-2-methylene-1,3,4,5,6,6a,7,8,8a,10,11,12,13,14b-tetradecahydropicene-4a-carboxylic acid	485.328	4.27	C30H46O5	NEG	485.3269958;53.92599487;439.3221436	17647128.22
(10S,11R)-10,11-dihydroxy-9-(hydroxymethyl)-1,6a,6b,9,12a-pentamethyl-2-methylidene-1,3,4,5,6,6a,7,8,8a,10,11,12,13,14b-tetradecahydropicene-4a-carboxylic acid	485.328	4.27	C30H46O5	NEG	485.3269958;53.92599487;439.3221436	17647128.22
Quillaic acid	485.328	4.27	C30H46O5	NEG	485.3269958;53.92599487;439.3221436	17647128.22
Asperphenamate	505.2138	4.27	C32H30N2O4	NEG	268.0979919;224.1082001;120.0456161;91.05543518;329.0672302;269.1013184;147.0453491;132.0456085;113.0245209;225.1118011;314.0420227;311.2956238;133.0535736;85.02967072;59.0139389;254.1179962;330.0710449	38888111.36
2-[2-[(Z)-3-(hydroxymethyl)-7-oxooct-3-enyl]-3-(2-hydroxypropan-2-yl)-1-methylcyclopentyl]acetic acid	353.2335	4.27	C20H34O5	NEG	353.2349854;293.2123413;59.01387787;275.201355;311.223175;309.2444153;335.2241211;235.1704254;291.2331543;353.1059265;267.1597595;171.1024323;223.1704254;191.0557861;294.2151184;249.149826;125.0979843;183.1389923;352.2114868;251.1669769;155.1077576;317.2140198;265.2179565;352.2615356;236.1733704;195.1395721;237.1512146;276.2051392;292.2382507;336.2276917;181.1243896;185.118927;169.1242523;265.1835632;310.2504272;137.0979156;151.1138306;249.2210236;237.1859894;201.1131897;211.1369934;187.0994263;279.1964417;209.1230469;209.1546326;321.2062988;308.2327881;74.0249176;295.1904602;157.1243286;183.1040497;205.1602936;232.3775482;255.1581268;164.685379;166.0918579;273.2253113;289.1816711;173.0467834;194.0749969;87.04540253;247.2808685;139.1143036	15660308.08
5-[5-hydroxy-3-(hydroxymethyl)pentyl]-8a-(hydroxymethyl)-5,6-dimethyl-3,4,4a,6,7,8-hexahydronaphthalene-1-carboxylic acid	353.2335	4.27	C20H34O5	NEG	353.2349854;293.2123413;59.01387787;275.201355;311.223175;309.2444153;335.2241211;235.1704254;291.2331543;353.1059265;267.1597595;171.1024323;223.1704254;191.0557861;294.2151184;249.149826;125.0979843;183.1389923;352.2114868;251.1669769;155.1077576;317.2140198;265.2179565;352.2615356;236.1733704;195.1395721;237.1512146;276.2051392;292.238250	15660308.08

Compound	mz	rt/min	FORMULA	type	MS2 ion fragment	Responsesignal
					7;336.2276917;181.1243896;185.118927;169.1242523;265.1835632;310.2504272;137.0979156;151.1138306;249.2210236;237.1859894;201.1131897;211.1369934;187.0994263;279.1964417;209.1230469;209.1546326;321.2062988;308.2327881;74.0249176;295.1904602;157.1243286;183.1040497;205.1602936;232.3775482;255.1581268;164.685379;166.0918579;273.2253113;289.1816711;173.0467834;194.0749969;87.04540253;247.2808685;139.1143036	
4,5-dihydroxy-2,2-bis(3-methylbut-2-enyl)cyclopent-4-ene-1,3-dione	263.129	4.28	C15H20O4	NEG	194.0584717;263.1292725;152.0116425;219.1390991;195.0643616;169.087265;153.0148926;124.0165634;125.0971603;220.1425781;166.0637054	37999378.55
2-decyl-3-hydroxy-pentanedioic acid	269.1759	4.29	C15H28O5	NEG	269.1760559;269.046051;237.1496124;251.1652679;207.1753845;205.1232605;225.1855011;238.1531067;161.1334381;252.1695557;151.0038452;59.0140419;125.0975952;149.0246582;57.03465271;208.1782837;71.0503006;254.0589905;225.0563507;149.0974579;165.0195007;206.1269684;107.0867004;117.0354462;99.08145142;209.1549683;223.1338501;226.0630798;177.128418;121.0293884	20665327.03
Hexylresorcinol	193.1234	4.31	C12H18O2	NEG	193.1234283;71.0139389;161.0457153;59.0138855;101.0244675;85.02954102;73.02955627;87.00874329;158.8471069;177.8605804;99.00898743;176.8589478;141.0194702;97.02970123;125.0243149	51284726.97
Pelargonic acid	157.1234	4.33	C9H18O2	NEG	157.1234131;100.9337158	568878891.5
Pseudolaric Acid B	413.1637	4.35	C23H28O8	NEG	116.9286575;413.1645813;100.9337082;237.0225067;377.1429749;311.2232056;333.1530762;101.0609131;221.0276184;313.2382507;99.92588043;395.1531677;193.0325165;171.1025085;165.0377808;117.928688;359.1333923;293.2121887;209.027832;187.0978699;378.1454773;99.00868988;84.93882751;181.0329285;255.0329285;201.1139679;251.0381775;125.0972137;61.98836136;219.13797;59.01399994;284.0358276;139.1126556;238.0244598;101.9337387;412.3114014;312.2280884;267.0344238;211.1338501;265.053833;222.0328217;115.0766449;225.0228577;197.1188049;269.0480347;116.0717926;223.0422058;334.15448;329.2342529;173.0819244;266.0249329;155.1079712;295.2272949;115.9205933;169.1234283;256.0383606;141.0915985;111.0817413;294.2155151;331.1397705;369.1738586;239.039032;277.054718;183.103363;75.00892639;285.0418701;255.1622162;229.144577;169.0873413;185.1181793;360.1324463;83.05043793;396.1550598;194.0362396;285.1706543	11033504.1
methyl (2S,4aR,6aR,7R,9S,10aS,10bR)-9-acetoxy-2-(3-furyl)-6a,10b-dimethyl-4,10-dioxo-2,4a,5,6,7,8,9,10a-octahydro-1H-benzo[f]isochromene-7-carboxylate	413.1637	4.35	C23H28O8	NEG	116.9286575;413.1645813;100.9337082;237.0225067;377.1429749;311.2232056;333.1530762;101.0609131;221.0276184;313.2382507;99.92588043;395.1531677;193.0325165;171.1025085;165.0377808;117.928688;359.1333923;293.2121887;209.027832;187.0978699;378.1454773;99.00868988;84.93882751;181.0329285;255.0329285;201.1139679;251.0381775;125.0972137;61.98836136;219.13797;59.01399994;284.0358276;139.1126556;238.0244598;101.9337387;412.3114014;312.2280884;267.0344238;211.1338501;265.053833;222.0328217;115.0766449;225.0228577;197.1188049;269.0480347;116.0717926;223.0422058;334.15448;329.2342529;173.0819244;266.0249329;155.1079712;295.2272949;115.9205933;169.1234283;256.0383606;141.0915985;111.0817413;294.2155151;331.1397705;369.1738586;239.039032;277.054718;183.103363;75.00892639;285.0418701;255.1622162;229.144577;169.0873413;185.1181793;360.1324463;83.05043793;396.1550598;194.0362396;285.1706543	11033504.1
13(S)-HpOTrE	309.2074	4.44	C18H30O4	NEG	291.1967468;309.2074585;209.1183929;247.2069702;292.2001343;171.1028442;155.108017;165.1284637;265.2160645;137.0973206;273.1860352;139.1125793;185.1186066;125.0972366;193.1239929;151.1134033;83.05031586;167.1081238;181.1236267;193.1595917;59.01387787;163.112	285263926.7

Compound	mz	rt/min	FORMULA	type	MS2 ion fragment	Responsesignal
					854;83.01377106;263.2011414;179.1440582;281.2124329;191.1082306;210.1212616;211.1340027;123.0812607;248.2098541;166.1000061;153.0918121;252.1362152;127.1127777;183.1388702;113.0968933;195.102356;180.1151581;57.03453445;97.06584167;125.0605545;98.03721619;141.1283417	
ethyl 2,2-dimethyl-3-(2-methylprop-1-enyl)cyclopropanecarboxylate	195.139	4.44	C12H20O2	NEG	195.1390686;59.01389313;177.1284332	471947789.5
1,11-dihydroxy-1,2,6a,6b,9,9,12a-heptamethyl-10-oxo-3,4,5,6,6a,7,8,8a,11,12,13,14b-dodecahydro-2H-picene-4a-carboxylic acid	485.3284	4.45	C30H46O5	NEG	485.3269958;53.92599487;439.3221436	10294282.24
(3aS,5aS,5bR,9S,11aR,13aR)-9-hydroxy-1-isopropenyl-5b,8,8,11a-tetramethyl-1,2,3,4,5,6,7,7a,9,10,11,11b,12,13,13a,13b-hexadecahydrocyclopenta[a]chrysene-3a,5a-dicarboxylic acid	485.3284	4.45	C30H46O5	NEG	485.3269958;53.92599487;439.3221436	10294282.24
(1R,2R,5S,8R,14R,15R,16S)-16-hydroxy-8-isopropenyl-1,2,14,17,17-pentamethyl-pentacyclo[11.7.0.02,10.05,9.014,18]icosane-5,15-dicarboxylic acid	485.3284	4.45	C30H46O5	NEG	485.3269958;53.92599487;439.3221436	10294282.24
10-Undecenoic acid	183.139	4.47	C11H20O2	NEG	183.1390228	1497391999
4,4,7a-trimethyl-3a,5,6,7-tetrahydro-3H-indene-1-carboxylic acid	207.139	4.47	C13H20O2	NEG	207.1390381;59.01382446;205.1230011;189.1290741	80302687.59
12(S)-HHTrE	279.1965	4.49	C17H28O3	NEG	279.1967468;278.2213745;278.1837463;235.2070923;237.1861115;261.1862488;59.01390076;85.06589508;57.03468323;222.1583252;235.1711121	36301660.81
ethyl octanoate	171.1391	4.51	C10H20O2	NEG	171.1391907	1122359466
Capric acid	171.1391	4.51	C10H20O2	NEG	171.1391907	1122359466
4-Ethyloctanoic acid	171.1391	4.51	C10H20O2	NEG	171.1391907	1122359466
beta-Chamigrenic acid	233.1547	4.54	C15H22O2	NEG	233.1546783	204445426.6
2,10,10-trimethyltricyclo[6.3.0.01,5]undec-6-ene-6-carboxylic acid	233.1547	4.54	C15H22O2	NEG	233.1546783	204445426.6
1,1,9a-trimethyl-3,4,8,9-tetrahydro-2H-benzo[7]annulene-7-carboxylic acid	233.1547	4.54	C15H22O2	NEG	233.1546783	204445426.6
(1S,5S,8S,9R)-2,5,9-trimethyltricyclo[6.3.0.01,5]undec-2-ene-3-carboxylic acid	233.1547	4.54	C15H22O2	NEG	233.1546783	204445426.6
(E)-5-(2,3-dimethyl-3-tricyclo[2.2.1.02,6]heptanyl)-2-methyl-pent-2-enoic acid	233.1547	4.54	C15H22O2	NEG	233.1546783	204445426.6
(2E,4Z)-9-oxooctadeca-2,4-dienoic acid	293.2123	4.56	C18H30O3	NEG	293.2123413;113.0973053;185.1184082;249.2224121;167.1081085;195.1392517;179.1079102;125.0976105	7006574438
9-Oxo-10(E),12(E)-octadecadienoic acid	293.2123	4.56	C18H30O3	NEG	293.2123413;113.0973053;185.1184082;249.2224121;167.1081085;195.1392517;179.1079102;125.0976105	7006574438
Medicagenic acid	501.3226	4.56	C30H46O6	NEG	501.3225403;439.322052;457.3323364;55.70327377;423.2903442;440.3260193;187.0976257;203.0928497;277.2180481;125.0971375;458.3369141	12201676.67
3,12-dihydroxy-4,6a,6b,11,12,14b-hexamethyl-1,2,3,4a,5,6,7,8,9,10,11,12a,14,14a-tetradecahydropicene-4,8a-dicarboxylic acid	501.3226	4.56	C30H46O6	NEG	501.3225403;439.322052;457.3323364;55.70327377;423.2903442;440.3260193;187.0976257;203.0928497;277.2180481;125.0971375;458.3369141	12201676.67
Hexadecanedioic acid	285.2072	4.58	C16H30O4	NEG	285.2072754;267.1966553;223.2067719;284.2675781;268.2001953;224.2101746;78.95915222;241.2168732;141.0921936;270.0425415	8928001.692
3-(2,5-dihydroxy-3-isopropyl-6-methyl-phenyl)-5-isopro	329.1761	4.59	C20H26O4	NEG	329.176178;311.1654968;217.1234741;285.1863098;283.1704407;151.0765991;261.1134949;243	76445942.71

Compound	mz	rt/min	FORMULA	type	MS2 ion fragment	Responsesignal
pyl-2-methyl-benzene-1,4-diol					.10289;312.1683655;267.1760864;286.1893616;199.1123352;109.0661774;107.086586;284.1741638	
Platycodigenin	519.333	4.59	C30H48O7	NEG	519.333252;475.3435059;457.3334045;501.3215942;241.1080017;57.70454025;413.3441162;125.0973358;439.3230896;483.3251648;476.3476563;197.1186066;171.1028748;223.0970764;303.2333069;458.3366089;347.2239075;287.2370911;331.2281494;481.315155;187.0978851;395.3320007;502.325592;225.1133881;429.3385925;293.2124939;305.2484436;419.2457581;59.01390839;311.2228088;57.81601334;295.2282104;411.3274231	15603540.49
(1S,4aR,6aS,6bR,10R,11R,12aR)-1,10,11-trihydroxy-9,9-bis(hydroxymethyl)-2,2,6a,6b,12a-pentamethyl-1,3,4,5,6,6a,7,8,8a,10,11,12,13,14b-tetradecahydropicene-4a-carboxylic acid	519.333	4.59	C30H48O7	NEG	519.333252;475.3435059;457.3334045;501.3215942;241.1080017;57.70454025;413.3441162;125.0973358;439.3230896;483.3251648;476.3476563;197.1186066;171.1028748;223.0970764;303.2333069;458.3366089;347.2239075;287.2370911;331.2281494;481.315155;187.0978851;395.3320007;502.325592;225.1133881;429.3385925;293.2124939;305.2484436;419.2457581;59.01390839;311.2228088;57.81601334;295.2282104;411.3274231	15603540.49
1,3,5,8-tetrahydroxy-2,4-bis(3-methylbut-2-enyl)xanthen-9-one	395.1532	4.60	C23H24O6	NEG	395.153656;116.9286499;100.9336929;376.2941589;84.93874359;99.9258728;78.95909882;394.3057861;280.2000122;333.1540527;117.928627;351.2938538;351.1670532;377.1429138;113.0973053;377.2733154;243.0330963;116.0720444;101.9338913;279.1964722;185.118515;359.1319885;295.2296753;186.1215363;315.14505;199.0427551;297.2073975;125.0979004;277.2172852;114.1011429;283.1922607;311.2219849;293.2122803;225.0233459	19805492.7
2,5-Di-tert-butylhydroquinone	221.1547	4.61	C14H22O2	NEG	221.1547546;59.01388931;219.1393585	239709851.7
N-Lauroylglycine	256.1919	4.61	C14H27NO3	NEG	74.0247879;256.1913757;255.2323761;212.2019806;59.01387787;75.02812958;183.1755371	27383221.18
(2Z)-2-[(4S,5S,6S,8S,9S,10R,13R,14S,16S)-6,16-diacetoxy-4,8,10,14-tetramethyl-3,7-dioxo-5,6,9,11,12,13,15,16-octahydro-4H-cyclopenta[a]phenanthren-17-ylidene]-6-methyl-hept-5-enoic acid	549.2838	4.64	C33H44O8	NEG	61.98828125;513.3458252;293.2115784;277.2165222;59.01383209;269.2122192;311.2227478;187.0978394;125.09729;155.1078186;171.1027527;209.1174011;237.1512299;548.3580322;211.1334686;137.098114;295.2268372;97.06632233;199.0964508;291.196991;505.3443604;215.092392;261.1358337;531.3458252;223.1702728;197.1553802;289.1777649;237.1155701;201.1128235;310.9976501;185.11763;103.7876129;527.3603516;527.5848389;116.9285278;159.0656891	1946656.95
3-[(2Z)-3,7-dimethylocta-2,6-dienyl]-2,4-dihydroxy-6-(2-phenylethyl)benzoic acid	375.1945	4.65	C25H30O4	NEG	78.95910645;375.1950989;96.96970367;357.2444153;313.2536926;374.2528687;331.2679138;221.1553192;357.1859436;277.2174988;96.00921631	20578603.45
13(S)-HODE	295.2275	4.66	C18H32O3	NEG	295.2279663;277.2174377;195.1390839;171.1027527;183.1390381;278.2211914;294.2157898;113.0973282	6137549212
(10E,12E)-9-hydroxyoctadeca-10,12-dienoic acid	295.2275	4.66	C18H32O3	NEG	295.2279663;277.2174377;195.1390839;171.1027527;183.1390381;278.2211914;294.2157898;113.0973282	6137549212
Undecanoic acid	185.1548	4.67	C11H22O2	NEG	185.1547546	185925242.7
18α-Glycyrrhetinic acid	469.3329	4.68	C30H46O4	NEG	469.3321533;423.3272095;52.14875793;157.0871887;424.3303528;311.2227478;451.3197937;171.1027985;437.1027222;425.3412781;173.0820007	373721.5919
(2S,4aS,6aS,6bR,10S,12aS,14bS)-10-hydroxy-2,4a,6a,6b,9,9,12a-heptamethyl-13-oxo-3,4,5,6,6a,7,8,8a,10,11,12,14b-dodecahydro-1H-picene-2-carboxylic acid	469.3329	4.68	C30H46O4	NEG	469.3321533;423.3272095;52.14875793;157.0871887;424.3303528;311.2227478;451.3197937;171.1027985;437.1027222;425.3412781;173.0820007	373721.5919
(2Z,6E,10E)-12-hydroxy-6,10-dimethyl-2-(4-methylpent-3-enyl)dodeca-2,6,10-trienoic acid	319.2278	4.68	C20H32O3	NEG	319.228363;301.2173157;318.2077637;213.1495819;71.05036926;275.2388306;121.1022949;275.2016296;263.202301;235.1708679;318.2506104;300.2053223	10050451.03
5-[(Z)-5-hydroxy-3-methyl-pent-3-enyl]-1,4a-dimethyl-6-methylene-decalin-1-carboxylic acid	319.2278	4.68	C20H32O3	NEG	319.228363;301.2173157;318.2077637;213.1495819;71.05036926;275.2388306;121.1022949;275.2016296;263.202301;235.1708679;318.2506104;300.2053223	10050451.03
14-(hydroxymethyl)-5,9-di	319.2278	4.68	C20H32	NEG	319.228363;301.2173157;318.2077637;213.1495	10050451.

Compound	mz	rt/min	FORMULA	type	MS2 ion fragment	Responsesignal
methyl-tetracyclo[11.2.1.01,10.04,9]hexadecane-5-carboxylic acid			O3		819;71.05036926;275.2388306;121.1022949;275.2016296;263.202301;235.1708679;318.2506104;300.2053223	03
(5S,9R)-14-(hydroxymethyl)-5,9-dimethyl-tetracyclo[11.2.1.01,10.04,9]hexadecane-5-carboxylic acid	319.2278	4.68	C20H32O3	NEG	319.228363;301.2173157;318.2077637;213.1495819;71.05036926;275.2388306;121.1022949;275.2016296;263.202301;235.1708679;318.2506104;300.2053223	10050451.03
(2E,6E,10E)-12-hydroxy-6,10-dimethyl-2-(4-methylpent-3-enyl)dodeca-2,6,10-trienoic acid	319.2278	4.68	C20H32O3	NEG	319.228363;301.2173157;318.2077637;213.1495819;71.05036926;275.2388306;121.1022949;275.2016296;263.202301;235.1708679;318.2506104;300.2053223	10050451.03
(E)-5-[(1R,3S,4aS,5S,8aS)-3-hydroxy-1,4a,5-trimethyl-2-methylidene-4,5,6,7,8,8a-hexahydro-3H-naphthalen-1-yl]-3-methylpent-2-enoic acid	319.2278	4.68	C20H32O3	NEG	319.228363;301.2173157;318.2077637;213.1495819;71.05036926;275.2388306;121.1022949;275.2016296;263.202301;235.1708679;318.2506104;300.2053223	10050451.03
3-(3,4-dihydroxyphenyl)-5,7-dihydroxy-6,8-bis(3-methylbut-2-enyl)chromone	421.1661	4.68	C25H26O6	NEG	421.1661072;337.1090698;309.1134949;365.1036377;366.1112061;393.1700134;365.1755981;219.0664978	36137942.55
beta-Estradiol	271.1707	4.70	C18H24O2	NEG	271.1730652;270.2159119;225.2225189;99.08163452;155.1443024;269.2130127;253.2169647;226.2254333;252.206604;97.06575775;127.1129837;113.0971603;227.1806946;98.06941223;235.2064362;59.01399994;141.1287231;227.2054749;169.1603394;227.2282867	11520197.68
Aphidicolin	337.2386	4.71	C20H34O4	NEG	337.239563;319.228241;275.2381287;293.2485657;320.2312927;276.2431641;294.2541809;301.2197571;336.2593994;277.2189026;59.01388931;57.03440475	34679251.97
4-[5-hydroxy-7-(4-hydroxyphenyl)heptyl]phenol	299.1652	4.72	C19H24O3	NEG	298.2470398;280.2364502;218.0950928;299.1669006;281.2407837;155.1081696;156.1114807;172.106369;171.1021576	21799619.83
(E)-6-(1,3-dihydroxy-4,4,8,10,14-pentamethyl-2,3,5,6,7,9,11,15,16,17-decahydro-1H-cyclopenta[a]phenanthren-17-yl)-2-methyl-hept-2-enoic acid	471.3492	4.72	C30H48O4	NEG	471.3478394;52.37273026	1250605.711
(1R,2R,4aS,6aS,6bR,10S,12aR)-1,10-dihydroxy-1,2,6a,6b,9,9,12a-heptamethyl-2,3,4,5,6,6a,7,8,8a,10,11,12,13,14b-tetradecahydropicene-4a-carboxylic acid	471.3492	4.72	C30H48O4	NEG	471.3478394;52.37273026	1250605.711
Pomolic acid	471.3492	4.72	C30H48O4	NEG	471.3478394;52.37273026	1250605.711
(E)-2-(3,5-dihydroxy-4,4,10,13-tetramethyl-2,3,6,7,11,12,14,15,16,17-decahydro-1H-cyclopenta[a]phenanthren-17-yl)-6-hydroxy-5,6-dimethyl-hept-4-en-3-one	471.3492	4.72	C30H48O4	NEG	471.3478394;52.37273026	1250605.711
(5R,10S,13R,14R,17R)-17-[(1R,4S)-4,5-dihydroxy-1,5-dimethyl-hexyl]-4,4,10,13,14-pentamethyl-2,5,6,11,12,15,16,17-octahydro-1H-cyclopenta[a]phenanthrene-3,7-dione	471.3492	4.72	C30H48O4	NEG	471.3478394;52.37273026	1250605.711
Octadecanedioic acid	313.2385	4.73	C18H34O4	NEG	295.2279358;313.2386169;312.2992859;251.2379303;201.1131744;296.2314453;125.0970078;183.1390076;171.1030884;129.092041;151.1129761;252.2407837;277.2170105;59.01380157;294.2162781;141.1288147;269.2478333;185.1188354;267.2326355;127.1124878;111.0817719	118550765.4
(E)-11-hydroxyoctadec-9-enoic acid	297.2434	4.73	C18H34O3	NEG	297.2436218;279.2331238;171.1027832;280.2365417;155.1078339	1104163785
9-Oxooctadecanoic acid	297.2434	4.73	C18H34O3	NEG	297.2436218;279.2331238;171.1027832;280.2365417;155.1078339	1104163785
cis-9,10-Epoxystearic acid	297.2434	4.73	C18H34O3	NEG	297.2436218;279.2331238;171.1027832;280.2365417;155.1078339	1104163785
(E)-6-hydroxyoctadec-4-enoic acid	297.2434	4.73	C18H34O3	NEG	297.2436218;279.2331238;171.1027832;280.2365417;155.1078339	1104163785
5-(5-methoxycarbonyl-5,8a-dimethyl-2-methylene-decalin-1-yl)-3-methyl-pentanoic acid	349.2379	4.78	C21H34O4	NEG	349.2381287;305.2487488;331.2282104;277.2171021;71.01390076;287.2382507;88.04042053;348.2564392;306.2522583;58.02981567;332.2315674;278.2200623;73.02932739	19414273.26

Compound	mz	rt/min	FORMULA	type	MS2 ion fragment	Responsesignal
Tetrahydrocorticosterone	349.2379	4.78	C21H34O4	NEG	349.2381287;305.2487488;331.2282104;277.2171021;71.01390076;287.2382507;88.04042053;348.2564392;306.2522583;58.02981567;332.2315674;278.2200623;73.02932739	19414273.26
methyl 14-hydroxy-14-(hydroxymethyl)-5,9-dimethyl-tetracyclo[11.2.1.01,10.04,9]hexadecane-5-carboxylate	349.2379	4.78	C21H34O4	NEG	349.2381287;305.2487488;331.2282104;277.2171021;71.01390076;287.2382507;88.04042053;348.2564392;306.2522583;58.02981567;332.2315674;278.2200623;73.02932739	19414273.26
2-Hydroxypalmitic acid	271.228	4.79	C16H32O3	NEG	271.2282715;271.17099;225.2225037;99.08166504;270.2160645;227.1803741;169.16008;85.06594086;269.2129211;155.1442719	61793550.66
16-Hydroxypalmitic acid	271.228	4.79	C16H32O3	NEG	271.2282715;271.17099;225.2225037;99.08166504;270.2160645;227.1803741;169.16008;85.06594086;269.2129211;155.1442719	61793550.66
hexyl hexanoate	199.1704	4.82	C12H24O2	NEG	199.1703644	186978859
Dodecanoic acid	199.1704	4.82	C12H24O2	NEG	199.1703644	186978859
(6aR,10aR)-6,6,9-trimethyl-3-propyl-6a,7,8,10a-tetrahydrobenzo[c]chromen-1-ol	285.1861	4.83	C19H26O2	NEG	285.1854248;284.2675171;241.1918793	5594202.122
4,6-dihydroxy-3-(6-hydroxy-1-oxo-3-pentyl-isochromen-8-yl)oxy-2-pentyl-benzoic acid	451.1797	4.83	C26H30O8	NEG	100.9336472;99.00882721;172.9547729;101.9337616;173.0819702;116.928627;451.1790771;115.9207458;154.9442444;111.0816727;450.3361511;277.2174683;255.2332611;433.1702271;332.2322693;59.01394653;403.158905;432.3208618	536501.6302
12-Hydroxystearic acid	299.2591	4.85	C18H36O3	NEG	299.2590332;298.2470703;281.2477112;253.2544403	88688319.25
(1R,5R,9S,15S)-15-acetoxy-5,9-dimethyl-14-methylene-tetracyclo[11.2.1.01,10.04,9]hexadecane-5-carboxylic acid	359.2228	4.85	C22H32O4	NEG	341.2112427;359.2211609;315.2325745;342.2149353;358.2463989;297.2230225;316.23526;191.1079559;275.2026672;192.113678;136.0530243;241.1603546;303.2347717;193.1239014;215.144104;99.08176422;172.0895538;235.0988007;233.154068;290.1548767;177.1278992;218.3467712;115.0984268;97.40900421	732618.5712
Stearidonic acid	275.2016	4.88	C18H28O2	NEG	275.2015381;231.2115631;59.01382828	1127366912
Myristoleic acid	225.186	4.91	C14H26O2	NEG	225.1860046	306801646.5
Testosterone	287.2017	4.92	C19H28O2	NEG	287.201355;80.96526337;286.1894226	9102985.745
3alpha-Hydroxy-5alpha-androstan-17-one (Androsterone)	289.2173	4.96	C19H30O2	NEG	289.2172546;288.2040405	26578247.46
2,4-dihydroxy-6-(hydroxymethyl)-3-[(6-hydroxy-1,2,5,5-tetramethyl-2,3,4,4a,6,7-hexahydronaphthalen-1-yl)methyl]benzaldehyde	387.2174	4.99	C23H32O5	NEG	128.0353851;372.194397;387.217804;369.2073059;191.0713196;373.1978455;217.0506439;328.204895;235.0613861;277.2171021;343.2645569;386.287384;370.2102966;125.0247345;259.1330261;126.0562515	2357664.624
[(1S,3R,3aS,4S,8aR)-1,3-dihydroxy-3-isopropyl-6,8a-dimethyl-1,2,3a,4,5,8-hexahydroazulen-4-yl] 4-methoxybenzoate	387.2174	4.99	C23H32O5	NEG	128.0353851;372.194397;387.217804;369.2073059;191.0713196;373.1978455;217.0506439;328.204895;235.0613861;277.2171021;343.2645569;386.287384;370.2102966;125.0247345;259.1330261;126.0562515	2357664.624
gamma-Linolenic acid	277.2169	5.01	C18H30O2	NEG	277.2172241;59.01386642	6665293995
alpha-Linolenic acid	277.2169	5.01	C18H30O2	NEG	277.2172241;59.01386642	6665293995
Isopimaric acid	301.217	5.02	C20H30O2	NEG	301.2175293;300.2624207;257.2274475;59.01387405;300.20578;273.1857605;283.170105;217.0873871;218.0946808;203.1802979;204.0792999;189.0921021;78.9590683;258.2299194;202.0988617;275.2013855;229.195816;274.1912537	11039936.08
Eicosapentaenoic acid	301.217	5.02	C20H30O2	NEG	301.2175293;300.2624207;257.2274475;59.01387405;300.20578;273.1857605;283.170105;217.0873871;218.0946808;203.1802979;204.0792999;189.0921021;78.9590683;258.2299194;202.0988617;275.2013855;229.195816;274.1912537	11039936.08
1,4a,7-trimethyl-7-vinyl-3,4,6,8,8a,9,10,10a-octahydro-2H-phenanthrene-1-carboxylic acid	301.217	5.02	C20H30O2	NEG	301.2175293;300.2624207;257.2274475;59.01387405;300.20578;273.1857605;283.170105;217.0873871;218.0946808;203.1802979;204.0792999;189.0921021;78.9590683;258.2299194;202.0988617;275.2013855;229.195816;274.1912537	11039936.08
Kaurenoic acid	301.217	5.02	C20H30O2	NEG	301.2175293;300.2624207;257.2274475;59.01387405;300.20578;273.1857605;283.170105;217.0873871;218.0946808;203.1802979;204.0792999;189.0921021;78.9590683;258.2299194;202.0988	11039936.08

Compound	mz	rt/min	FORMULA	type	MS2 ion fragment	Responses ignal
(5R,9S)-5,9-dimethyl-14-methylene-tetracyclo[11.2.1.01,10.04,9]hexadecane-5-carboxylic acid	301.217	5.02	C20H30O2	NEG	301.2175293;300.2624207;257.2274475;59.01387405;300.20578;273.1857605;283.170105;217.0873871;218.0946808;203.1802979;204.0792999;189.0921021;78.9590683;258.2299194;202.0988617;275.2013855;229.195816;274.1912537	11039936.08
Continentalic acid	301.217	5.02	C20H30O2	NEG	301.2175293;300.2624207;257.2274475;59.01387405;300.20578;273.1857605;283.170105;217.0873871;218.0946808;203.1802979;204.0792999;189.0921021;78.9590683;258.2299194;202.0988617;275.2013855;229.195816;274.1912537	11039936.08
(1S,4aR,10aR)-1,4a,7-trimethyl-7-vinyl-3,4,4b,5,6,9,10,10a-octahydro-2H-phenanthrene-1-carboxylic acid	301.217	5.02	C20H30O2	NEG	301.2175293;300.2624207;257.2274475;59.01387405;300.20578;273.1857605;283.170105;217.0873871;218.0946808;203.1802979;204.0792999;189.0921021;78.9590683;258.2299194;202.0988617;275.2013855;229.195816;274.1912537	11039936.08
Brevicuspisaponin 2	485.327	5.07	C30H48O6	NEG	277.2173157;207.1026306;59.0138855;163.1128845;278.2206421;485.3276062;225.1131287;208.1063232;100.9335251;187.0980835;164.1165009;125.0970154;145.1023865;169.0870361	698371.5838
(1R,2R,4aS,6aS,6bR,10R,11S,12R,12aR)-1,10,11,12-tetrahydroxy-1,2,6a,6b,9,9,12a-heptamethyl-2,3,4,5,6,6a,7,8,8a,10,11,12,13,14b-tetradecahydropicene-4a-carboxylic acid	485.327	5.07	C30H48O6	NEG	277.2173157;207.1026306;59.0138855;163.1128845;278.2206421;485.3276062;225.1131287;208.1063232;100.9335251;187.0980835;164.1165009;125.0970154;145.1023865;169.0870361	698371.5838
Bevirimat	583.4011	5.08	C36H56O6	NEG	305.1758728;277.2172546;287.1652832;436.4527588;171.1026764;306.1791992;243.1754303;583.3909912;179.1076965;247.1341553;121.0660172;278.2204285;288.1686707;265.1443481;164.071228;235.1345978;189.1286469;223.1339417;151.0762939;199.1343231;291.1974487;133.0656586;109.0656738;263.1641541;411.2910767;149.096817;269.1524658;203.1444702;191.1434937;85.06585693;135.0812073;183.1018982;175.113266;185.1182556;565.3881226;125.0973129;261.1873779;161.0610657;303.2322083;139.112793;197.1179962;279.2322998;249.1494751;271.2267761;582.4004517;201.1131287;123.0815582;237.1489258;107.0502396;162.0679779;161.09729;289.1802368;307.1906433;412.2936401;547.3881836;95.05051422;311.2216797;64.94608307;187.0978851;229.1229858;221.1184235;151.112793	3302633.065
Ursolic acid	455.3545	5.08	C30H48O3	NEG	455.3526917;50.59545898	1291472.47
3-Epiursolic Acid	455.3545	5.08	C30H48O3	NEG	455.3526917;50.59545898	1291472.47
(4aS,6aS,6bR,10S,12aR)-10-hydroxy-2,2,6a,6b,9,9,12a-heptamethyl-1,3,4,5,6,6a,7,8,8a,10,11,12,13,14b-tetradecahydropicene-4a-carboxylic acid	455.3545	5.08	C30H48O3	NEG	455.3526917;50.59545898	1291472.47
3-Epioleanolic acid	455.3545	5.08	C30H48O3	NEG	455.3526917;50.59545898	1291472.47
β-Boswellic acid	455.3545	5.08	C30H48O3	NEG	455.3526917;50.59545898	1291472.47
Myristic acid	227.2015	5.09	C14H28O2	NEG	227.2016144	1003929048
12-Methyltridecanoic acid	227.2015	5.09	C14H28O2	NEG	227.2016144	1003929048
(E)-5-(1,2,4a,5-tetramethyl-2,3,4,7,8,8a-hexahydronaphthalen-1-yl)-3-methyl-pent-2-enoic acid	303.2329	5.11	C20H32O2	NEG	303.2329712;302.2210999;59.01388931;259.2421875	12666847.36
(10Z)-3a,10-dimethyl-1-prop-1-en-2-yl-2,3,4,5,6,7,8,9,12,12a-decahydro-1H-cyclopenta[11]annulene-6-carboxylic acid	303.2329	5.11	C20H32O2	NEG	303.2329712;302.2210999;59.01388931;259.2421875	12666847.36
Arachidonic acid (AA)	303.2329	5.11	C20H32O2	NEG	303.2329712;302.2210999;59.01388931;259.2421875	12666847.36
cis-9-Palmitoleic acid	253.2172	5.11	C16H30O2	NEG	253.2173004	328029960.6
5-(4-carboxy-3-methyl-butyl)-1,4a-dimethyl-6-methylen	335.2229	5.11	C20H32O4	NEG	75.00878906;277.2173157;74.02481842;278.220459;335.2221375;334.2398071;291.2336426;76.	3531522.296

Compound	mz	rt/min	FORMULA	type	MS2 ion fragment	Responsesignal
e-decalin-1-carboxylic acid					01220703	
6-[(2E)-3,7-dimethylocta-2,6-dienyl]-7-hydroxy-chromen-2-one	297.1529	5.12	C19H22O3	NEG	297.1529846;59.01386261;183.0119934;296.2319031;225.2220612	192689830.3
8-[(2E)-3,7-dimethylocta-2,6-dienyl]-7-hydroxy-chromen-2-one	297.1529	5.12	C19H22O3	NEG	297.1529846;59.01386261;183.0119934;296.2319031;225.2220612	192689830.3
(Z)-2-hydroxyoctadec-9-enoic acid	297.2433	5.16	C18H34O3	NEG	297.1531372;297.2431335;251.2379913;183.0120392;252.2410431;184.0193329;253.253006	45701858.35
(3,11-dihydroxy-7,7,11-trimethyl-6,15-dioxatetracyclo[10.2.1.05,14.08,13]pentadeca-1(14),2,4-trien-4-yl)-phenyl-methanone	379.1581	5.16	C23H24O5	NEG	379.1583252;116.9286194;99.92584229;100.9336624;84.93872833;116.0718155;378.3021851;361.1478577;335.1688232;317.1581421;343.1374817;117.9285431;101.9338455;318.1610718;362.1491699;277.2168274;85.93920898;86.95427704;336.1714172;344.1397095;229.0167389	99789227.15
Orthosphenic acid	487.3426	5.18	C30H48O5	NEG	59.01387787;277.2173157;279.2329407;207.1025238;209.1183167;487.346344;111.0815964;256.2363586;255.2330627;163.1130066;278.221283;173.0821075;112.0851135;280.2363281;227.1292419;174.0858307;225.1128693;169.0865784;486.352417;230.1126709;229.1080322;247.1182861;257.2390442;210.1222382;248.1230164;208.1062164;125.0970688;226.1164551;83.05027771;54.14993668;60.01732254;281.2477112;486.2729187	5389198.087
Arjunolic acid	487.3426	5.18	C30H48O5	NEG	59.01387787;277.2173157;279.2329407;207.1025238;209.1183167;487.346344;111.0815964;256.2363586;255.2330627;163.1130066;278.221283;173.0821075;112.0851135;280.2363281;227.1292419;174.0858307;225.1128693;169.0865784;486.352417;230.1126709;229.1080322;247.1182861;257.2390442;210.1222382;248.1230164;208.1062164;125.0970688;226.1164551;83.05027771;54.14993668;60.01732254;281.2477112;486.2729187	5389198.087
10,11-dihydroxy-9-(hydroxymethyl)-1,2,6a,6b,9,12a-hexamethyl-2,3,4,5,6,6a,7,8,8a,10,11,12,13,14b-tetradecahydro-1H-picene-4a-carboxylic acid	487.3426	5.18	C30H48O5	NEG	59.01387787;277.2173157;279.2329407;207.1025238;209.1183167;487.346344;111.0815964;256.2363586;255.2330627;163.1130066;278.221283;173.0821075;112.0851135;280.2363281;227.1292419;174.0858307;225.1128693;169.0865784;486.352417;230.1126709;229.1080322;247.1182861;257.2390442;210.1222382;248.1230164;208.1062164;125.0970688;226.1164551;83.05027771;54.14993668;60.01732254;281.2477112;486.2729187	5389198.087
(4aS,6aS,6bR,9R,10R,11R,12aR)-10,11-dihydroxy-9-(hydroxymethyl)-2,2,6a,6b,9,12a-hexamethyl-1,3,4,5,6,6a,7,8,8a,10,11,12,13,14b-tetradecahydropicene-4a-carboxylic acid	487.3426	5.18	C30H48O5	NEG	59.01387787;277.2173157;279.2329407;207.1025238;209.1183167;487.346344;111.0815964;256.2363586;255.2330627;163.1130066;278.221283;173.0821075;112.0851135;280.2363281;227.1292419;174.0858307;225.1128693;169.0865784;486.352417;230.1126709;229.1080322;247.1182861;257.2390442;210.1222382;248.1230164;208.1062164;125.0970688;226.1164551;83.05027771;54.14993668;60.01732254;281.2477112;486.2729187	5389198.087
(1S,2R,4aS,6aS,6bR,9R,10R,11R,12aR)-10,11-dihydroxy-9-(hydroxymethyl)-1,2,6a,6b,9,12a-hexamethyl-2,3,4,5,6,6a,7,8,8a,10,11,12,13,14b-tetradecahydro-1H-picene-4a-carboxylic acid	487.3426	5.18	C30H48O5	NEG	59.01387787;277.2173157;279.2329407;207.1025238;209.1183167;487.346344;111.0815964;256.2363586;255.2330627;163.1130066;278.221283;173.0821075;112.0851135;280.2363281;227.1292419;174.0858307;225.1128693;169.0865784;486.352417;230.1126709;229.1080322;247.1182861;257.2390442;210.1222382;248.1230164;208.1062164;125.0970688;226.1164551;83.05027771;54.14993668;60.01732254;281.2477112;486.2729187	5389198.087
Bayogenin	487.3426	5.18	C30H48O5	NEG	59.01387787;277.2173157;279.2329407;207.1025238;209.1183167;487.346344;111.0815964;256.2363586;255.2330627;163.1130066;278.221283;173.0821075;112.0851135;280.2363281;227.1292419;174.0858307;225.1128693;169.0865784;486.352417;230.1126709;229.1080322;247.1182861;257.2390442;210.1222382;248.1230164;208.1062164;125.0970688;226.1164551;83.05027771;54.14993668;60.01732254;281.2477112;486.2729187	5389198.087
(1S,2R,4aS,6aS,6bR,9R,10S,11R,12aR,14bS)-10,11-dihydroxy-9-(hydroxymethyl)-	487.3426	5.18	C30H48O5	NEG	59.01387787;277.2173157;279.2329407;207.1025238;209.1183167;487.346344;111.0815964;256.2363586;255.2330627;163.1130066;278.221283	5389198.087

Compound	mz	rt/min	FORMULA	type	MS2 ion fragment	Responsesignal
1,2,6a,6b,9,12a-hexamethyl-2,3,4,5,6,6a,7,8,8a,10,11,12,13,14b-tetradecahydro-1H-picene-4a-carboxylic acid					;173.0821075;112.0851135;280.2363281;227.1292419;174.0858307;225.1128693;169.0865784;486.352417;230.1126709;229.1080322;247.1182861;257.2390442;210.1222382;248.1230164;208.1062164;125.0970688;226.1164551;83.05027771;54.14993668;60.01732254;281.2477112;486.2729187	
(4aS,6aS,6bR,9R,10S,11R,12aR)-10,11-dihydroxy-9-(hydroxymethyl)-2,2,6a,6b,9,12a-hexamethyl-1,3,4,5,6,6a,7,8,8a,10,11,12,13,14b-tetradecahydropicene-4a-carboxylic acid	487.3426	5.18	C30H48O5	NEG	59.01387787;277.2173157;279.2329407;207.1025238;209.1183167;487.346344;111.0815964;256.2363586;255.2330627;163.1130066;278.221283;173.0821075;112.0851135;280.2363281;227.1292419;174.0858307;225.1128693;169.0865784;486.352417;230.1126709;229.1080322;247.1182861;257.2390442;210.1222382;248.1230164;208.1062164;125.0970688;226.1164551;83.05027771;54.14993668;60.01732254;281.2477112;486.2729187	5389198.087
Euscaphic acid	487.3426	5.18	C30H48O5	NEG	59.01387787;277.2173157;279.2329407;207.1025238;209.1183167;487.346344;111.0815964;256.2363586;255.2330627;163.1130066;278.221283;173.0821075;112.0851135;280.2363281;227.1292419;174.0858307;225.1128693;169.0865784;486.352417;230.1126709;229.1080322;247.1182861;257.2390442;210.1222382;248.1230164;208.1062164;125.0970688;226.1164551;83.05027771;54.14993668;60.01732254;281.2477112;486.2729187	5389198.087
1,10-dihydroxy-9-(hydroxymethyl)-1,2,6a,6b,9,12a-hexamethyl-2,3,4,5,6,6a,7,8,8a,10,11,12,13,14b-tetradecahydropicene-4a-carboxylic acid	487.3426	5.18	C30H48O5	NEG	59.01387787;277.2173157;279.2329407;207.1025238;209.1183167;487.346344;111.0815964;256.2363586;255.2330627;163.1130066;278.221283;173.0821075;112.0851135;280.2363281;227.1292419;174.0858307;225.1128693;169.0865784;486.352417;230.1126709;229.1080322;247.1182861;257.2390442;210.1222382;248.1230164;208.1062164;125.0970688;226.1164551;83.05027771;54.14993668;60.01732254;281.2477112;486.2729187	5389198.087
(4aR,5R,6aR,6aS,6bR,8aR,9R,10S,12aR,14bS)-5,10-dihydroxy-9-(hydroxymethyl)-2,2,6a,6b,9,12a-hexamethyl-1,3,4,5,6,6a,7,8,8a,10,11,12,13,14b-tetradecahydropicene-4a-carboxylic acid	487.3426	5.18	C30H48O5	NEG	59.01387787;277.2173157;279.2329407;207.1025238;209.1183167;487.346344;111.0815964;256.2363586;255.2330627;163.1130066;278.221283;173.0821075;112.0851135;280.2363281;227.1292419;174.0858307;225.1128693;169.0865784;486.352417;230.1126709;229.1080322;247.1182861;257.2390442;210.1222382;248.1230164;208.1062164;125.0970688;226.1164551;83.05027771;54.14993668;60.01732254;281.2477112;486.2729187	5389198.087
(1R,2R,5S,8R,12R,16R,17S,18S)-16-hydroxy-1,2,17-trimethyl-14-oxo-8,18-bis(prop-1-en-2-yl)-13-oxapentacyclo[10.8.1.02,10.05,9.017,21]henicosane-5-carboxylic acid	483.3118	5.21	C30H44O5	NEG	483.3112183;275.1289978;233.0819702;276.1359253;208.0741577;343.1920776;221.0819244;53.70204926;344.1977844;345.169342;234.0848389;281.2473755;200.0928345;261.1125488;277.1391296;369.2810669;255.2336884	8767644.884
2-Hydroxystearic acid	299.259	5.22	C18H36O3	NEG	299.2591858;253.2536011;254.2570801;281.2485657;251.238739	13592823.78
Pentadecanoic acid	241.2171	5.22	C15H30O2	NEG	241.2172394;84.96736145;51.85725403;70.62657928;194.784668;71.48789215;67.25458527;66.3136673	4620372.57
Methyl pentadecanoate	255.2329	5.31	C16H32O2	NEG	255.2328186	328907741.3
Palmitic acid	255.2329	5.31	C16H32O2	NEG	255.2328186	328907741.3
Cer(d18:1/4:0)	368.3168	5.32	C22H43NO3	NEG	368.3170776;130.0874023;324.3275146;281.2485046;131.0911713;102.0563126;325.332489;116.0717163;254.2497406	7274439.723
Tetradecyl sulfate (sodium)	293.1791	5.32	C14H30O4S	NEG	293.1790161;96.96017456;292.2032471	37082489.32
2-(14-methylpentadecanoylamino)-3-phenyl-propanoic acid	402.3016	5.32	C25H41NO3	NEG	402.3010254;164.0716858;254.2488098;147.0451965;91.05532837;277.2175903;165.0751495;266.2485962;143.0713806;78.95906067;255.22789;125.0609512;358.3168945;359.2989197;103.0551682;289.0540466;72.00930786	1724775.094
5Z,8Z,11Z-Eicosatrienoic acid	305.2489	5.32	C20H34O2	NEG	305.2486572	1559847.166
cis-8,11,14-Eicosatrienoic acid	305.2489	5.32	C20H34O2	NEG	305.2486572	1559847.166
1-(2,6-dihydroxyphenyl)-9-	325.1843	5.32	C21H26	NEG	325.1844788;183.0121002;184.0202332	373548999

Compound	mz	rt/min	FORMULA	type	MS2 ion fragment	Responsesignal
phenyl-nonan-1-one			O3			.5
cis-11.14-Eicosadienoic acid	307.2642	5.37	C20H36O2	NEG	307.2640686;306.2174988	8292885.658
[3-[2,3-dihydroxypropoxy(hydroxy)phosphoryl]oxy-2-hydroxypropyl] 14-methylpentadecanoate	483.2733	5.40	C22H45O9P	NEG	255.2328949;256.2361755;483.2729492;152.9958954;227.0326691;78.95905304;257.2390137;53.69778442;171.0066071;245.0433044;153.9994202;53.80925751	13262809.64
LPG(18:1(9Z))	509.2891	5.42	C24H47O9P	NEG	281.2484741;509.2881775;152.9958801;282.2519531;227.0325775;56.58837509;78.95899963;280.2359619;279.2322083;255.233139;245.0433044;171.006897	2098004.492
Heptadecanoic acid	269.2486	5.43	C17H34O2	NEG	269.2485352	16835819.56
Kermadecin H	409.2365	5.45	C26H34O4	NEG	152.9959106;78.95906067;409.2356873;255.2328186;171.0065002;96.96961212;153.9994659;277.217865	4021114.942
Nervonic acid	365.3425	5.47	C24H46O2	NEG	365.3426208;80.96520233	3873363.768
1-Oleoyl-sn-glycerol 3-phosphate	435.2523	5.47	C21H41O7P	NEG	152.9959412;78.95909882;435.2530212;116.9286194;125.0608978;123.0816269;126.0321426;171.0061951;281.2481995;417.3379822;373.3484497;153.9992676;96.96973419;279.2329712;97.06591797;277.2184448;391.3587341	3296461.782
Stearic acid	283.2642	5.54	C18H36O2	NEG	283.2640686;282.2527161	63693284.93
Paullinic acid	309.2799	5.55	C20H38O2	NEG	309.2800598	11078720
trans-11-Eicosenoic acid	309.2799	5.55	C20H38O2	NEG	309.2800598	11078720
Phytanic acid	311.296	5.66	C20H40O2	NEG	311.2957458	210959.1938
Nonadecanoic acid	297.2798	5.68	C19H38O2	NEG	297.2798767	3927954.614
Pristanic acid	297.2798	5.68	C19H38O2	NEG	297.2798767	3927954.614
5-nonadecylbenzene-1,3-diol	375.327	5.84	C25H44O2	NEG	375.3265381;78.95908356;96.96967316;374.315979	13852974.83
Erucic acid	337.3111	5.88	C22H42O2	NEG	337.3114929	358680267.6
Oleic acid	281.2483	5.90	C18H34O2	NEG	281.2485046;280.2363281	540172066.8
trans-Vaccenic acid	281.2483	5.90	C18H34O2	NEG	281.2485046;280.2363281	540172066.8
17-ODYA	279.2327	5.95	C18H32O2	NEG	279.2328796;278.2209778	1095890127
Linoleic acid	279.2327	5.95	C18H32O2	NEG	279.2328796;278.2209778	1095890127

Supplementarey Table S3 The table of biomarkers intersecting between “Perilla Seed Oil” and “hyperlipidemia”.

Common Target								
ADRB2	CTNNB1	XDH	SLC22A5	PLB1	LRP1	IL10	F3	AGXT
ADRB1	CSF2	WT1	SELE	PECAM1	LEP	IFNG	F13A1	SLC25A13
ADRA2A	CKM	VEGFA	RXRG	OTC	LCAT	ICAM1	F10	PON1
ADIPOQ	CCND1	VCAM1	RXRB	NR3C1	KDF1	HNF4A	ESR2	MMP3
ACP5	CCL5	TP53	RXRA	NR1I2	ITGA3	HMOX1	ESR1	IL1A
ACHE	CCL2	TNF	REN	NOS3	INSR	GNMT	EMP2	FABP1
AARS1	CAV1	TGFB1	PYGM	NOS2	INS	FN1	EGF	CXCL8
MMP14	CAT	STAT1	PTEN	NFKB1	IL6	FLT1	EDN1	IL1B
IL18	BDNF	SOD1	PPARG	MTR	IL4	FASN	DDIT3	FAH
F7	BCHE	SLPI	PPARD	MPO	IL2	FASLG	CYP3A4	CYP1A1
CXCL12	APOA1	SLC2A4	PPARA	MMP9				

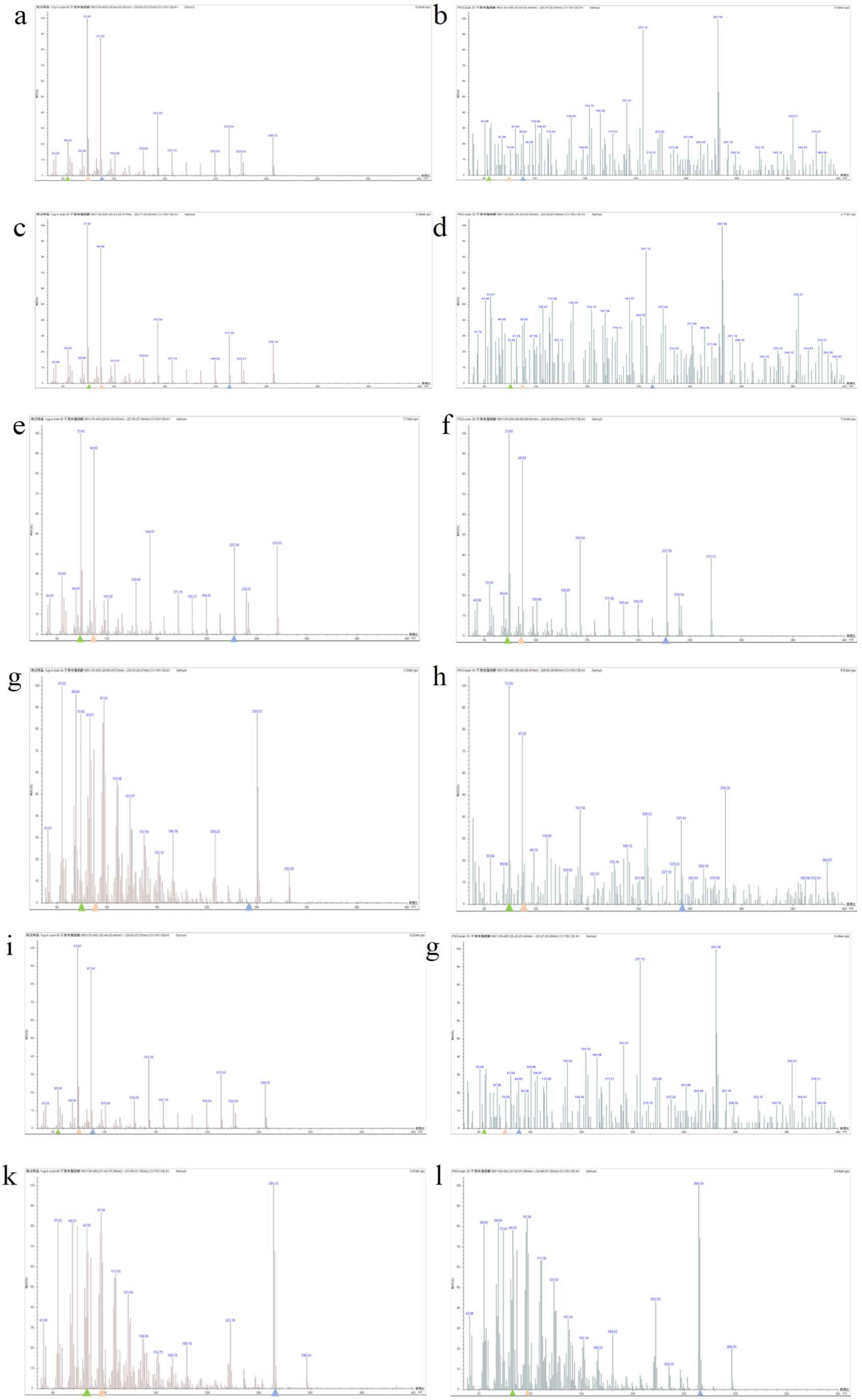
a
b
c
d
e
f
g
h
i
g
k
l

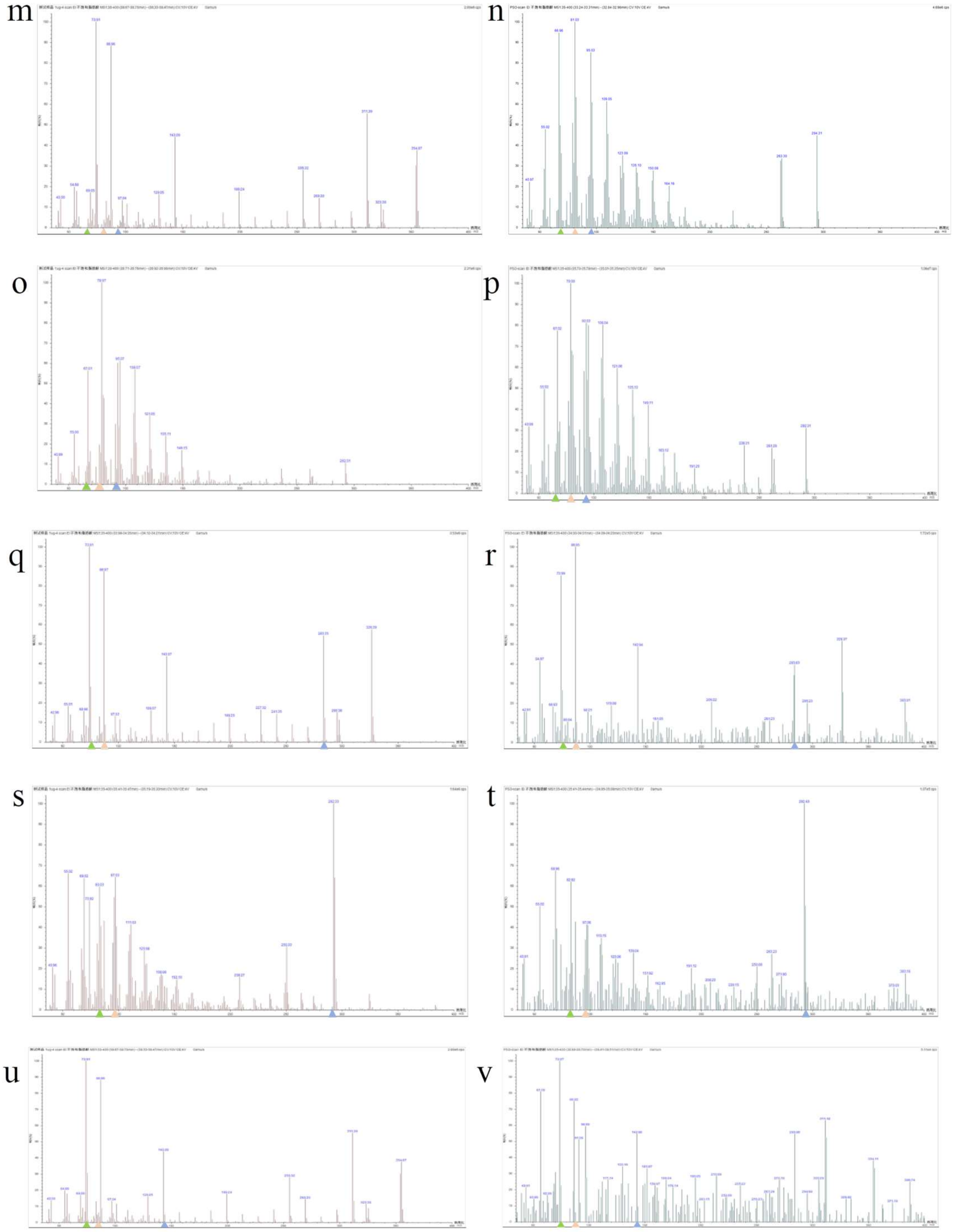

Supplementarey Figure S1. Methyl Fatty Acid GC-MS/MS Spectrum of Perilla Seed Oil. a, chromatogram of methyl myristoleate standard. b, chromatogram of methyl myristoleate in perilla seed oil sample. c, chromatogram of methyl pentadecanoate standard. d chromatogram of pentadecanoate in perilla seed oil sample. e, chromatogram of methyl palmitate standard. f, chromatogram of methyl palmitate in perilla seed oil sample. g, chromatogram of methyl heptadecanoate standard. h, chromatogram of methyl heptadecanoate in perilla seed oil sample. i, chromatogram of methyl stearate standard. j, chromatogram of methyl stearate in perilla seed oil sample. k, chromatogram of methyl oleate standard. l,

chromatogram of methyl oleate in perilla seed oil sample. m, chromatogram of methyl linoleate standard. n, chromatogram of methyl linoleate in perilla seed oil sample. o, chromatogram of methyl α-linolenate standard. p, chromatogram of methyl α-linolenate in perilla seed oil sample. q, chromatogram of methyl arachidonate standard. r, chromatogram of methyl arachidonate in perilla seed oil sample. s, chromatogram of methyl gadoleate standard. t, chromatogram of methyl gadoleate in perilla seed oil sample. u, chromatogram of methyl docosanoate standard. v, chromatogram of methyl docosanoate in perilla seed oil sample.

Supplementarey Table S4 GC-MS/MS calibration data, linear equations, and R^2 for fatty acid methyl esters.

Number	Identification	Linear equation	R^2	LOD (μg/mL)	LOQ (μg/mL)
1	Methyl myristoleate	y=1800.1246x-19002.2437	0.9999	0.010	0.033
2	Methyl pentadecanoate	y=1430.4642x-23385.0670	0.9996	0.005	0.018
3	Methyl palmitate	y=3375.0094x-26011.1622	0.9998	0.006	0.019
4	Methyl heptadecanoate	y=1602.2155x-37792.5823	0.9998	0.009	0.030
5	Methyl stearate	y=1511.8919x-42204.3331	0.9996	0.014	0.048
6	Methyl oleate	y=638.2050 x-7065.3068	0.9991	0.054	0.179
7	Methyl linoleate	y=713.2604 x-16088.5682	0.9997	0.044	0.147
8	Methyl α-linolenate	y=837.2656 x -21154.7378	0.9994	0.079	0.264
9	Methyl arachidonate	y=1255.1708x-53794.3828	0.9990	0.023	0.076
10	Methyl gadoleate	y=466.5031x-29024.28008	0.9991	0.040	0.132
11	Methyl docosanoate	y=972.1004 x-55738.7855	0.9993	0.039	0.130

y: response (%); x: concentration (ng/mL)

Supplementarey Table S5 PSO affects serum lipid levels in rats.

Group	TC(mmol/L)	TG(mmol/L)	HDL-C(mmol/L)	LDL-C(mmol/L)
Control	1.98±0.23	1.63±0.08	1.51±0.16	0.62±0.06
Model	3.55±0.51 ##	2.52±0.30 ##	1.63±0.12 ns	0.88±0.18 ##
PSO-L	2.85±0.36 *	2.42±0.38 ns	1.52±0.13 ns	0.61±0.10 **
PSO-M	1.95±0.31 **	2.15±0.23 ns	1.51±0.14 ns	0.58±0.09 **

PSO-H	2.87±0.50 *	2.40±0.28 ^{ns}	1.64±0.19 ^{ns}	0.61±0.12 **

Data are presented as mean ± standard deviation (n = 6). $^{\#\#}$ $p < 0.01$ vs. control group; *$p < 0.05$, **$p < 0.01$ vs. model group. ns means p > 0.05 vs. control group or vs. model group.

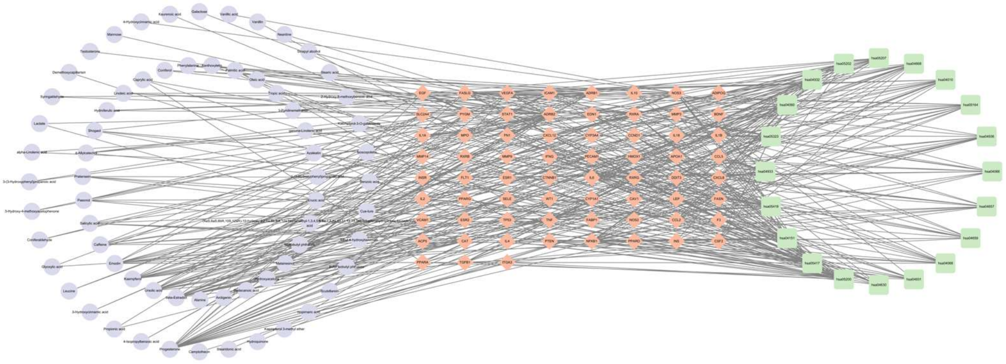

Supplementarey Figure S2. Network of compound-target-pathway.

Supplementarey Table S6 Identification results of differential serum metabolites in control vs. PSO groups.

Serum metabolites	VIP	PSO vs control	Serum metabolites	VIP	PSO vs control
Prostaglandin E2 (PGE2)	2.29	↓##	Tetrahydrocorticosterone	2.31	↑##
Palmitic acid	1.99	↓#	3-Hydroxylauric acid	2.13	↓##
Valerylcarnitine	1.78	↓#	Denticetic-acid	2.13	↓##
Isovalerylcarnitine (Car(5:0))	1.78	↓#	Phenylsulfate	1.63	↓##
Cholic acid 3-glucuronide	1.73	↑#	Stearidonic acid	2.27	↑##
Dodecanoic acid	1.68	↓#	Valeric acid	1.94	↓##
1-Myristoyl-sn-glycero-3-phosphocholine (LPC(14:0/0:0))	1.69	↓#	Isovaleric acid	1.94	↓##
p-Cresol glucuronide	1.71	↓#	5-PAHSA	2.02	↓##
2-Methylhippuric acid	1.51	↓#	9-PAHSA	2.02	↓##
Adenosine	1.66	↓#	2-Methylbenzoxazole	1.80	↓##
Taurocholic acid	1.75	↓#	5-Hydroxyindole	1.80	↓##
Tauro-beta-muricholic acid	1.75	↓#	3,6-Dioxo-5alpha-cholan-24-oic acid	2.36	↑##
3-Hydroxydecanoic acid	1.87	↓#	cis-11.14-Eicosadienoic acid	1.93	↓##
Glycine	1.73	↑#	Myristoylcarnitine	2.05	↓##
Pyruvate	1.66	↓#	alpha-Linolenic acid	2.42	↑##
Dihydroorotic acid	1.72	↓#	gamma-Linolenic acid	2.42	↑##
Tirofiban (hydrochloride monohydrate)	1.67	↓#	2-Ketocaproic acid	1.93	↓##
N,N-Diethyl-2-aminoethanol	1.63	↑#	3-Methyl-2-oxovaleric acid	1.93	↓##
Decanoylcarnitine (Car(10:0))	1.71	↓#	Ketoleucine	1.93	↓##
alpha-Tocopherol (Vitamin E)	1.70	↓#	Dihydrorotenone	2.44	↑##
Nicotinamide N-oxide	1.65	↑#	Arachidonoylcarnitine (Car(20:4))	2.25	↓##
4-Nitroaniline	1.65	↑#	Glycerophosphocholine	2.23	↑##
Azelaic acid	2.02	↑#	Uracil	2.03	↓##
Arachidonic acid (AA)	1.85	↓#	3-methylcytidine	1.99	↓##
13(S)-HpOTrE	2.38	↑#	5-hydroxymethyl-2'-deoxycytidine	1.99	↓##
Formylanthranilic acid	2.03	↓#	gamma-CEHC (gamma-Carboxyethyl hydrochroman)	1.96	↓##
Cholic acid 7-sulfate	1.82	↓#	Indoxyl sulfate	1.82	↓##
Pimelic acid	1.88	↑#	cis-4-Decenoylcarnitine	2.08	↓##
3-Methyladipic acid	1.88	↑#	Lauroylcarnitine	2.06	↓##
Benzoic acid	1.87	↓#	trans-2-Octenoic acid	2.33	↑##

p-Toluquinone	1.87	$\downarrow^{\#}$	Palmitoylcarnitine (Car(16:0))	2.13	$\downarrow^{\#\#}$
3-hydroxybenzaldehyde	1.87	$\downarrow^{\#}$	5-Hydroxyhexanoic acid	2.33	$\uparrow^{\#\#}$
4-Hydroxybenzaldehyde	1.87	$\downarrow^{\#}$	3-O-Acetylursolic acid	2.44	$\uparrow^{\#\#}$
cis-9-Palmitoleic acid	2.01	$\downarrow^{\#}$	PC(14:0/16:0)	2.10	$\downarrow^{\#\#}$
12-Methyltridecanoic acid	1.75	$\downarrow^{\#}$	Serine	2.13	$\uparrow^{\#\#}$
Myristic acid	1.75	$\downarrow^{\#}$	LPC(18:2/0:0)	2.20	$\uparrow^{\#\#}$
alpha-Ketoisovaleric acid	1.87	$\downarrow^{\#}$	Euscaphic acid	2.28	$\uparrow^{\#\#}$
LPC(20:0)	1.87	$\uparrow^{\#}$	Protoporphyrin IX	1.19	$\downarrow^{\#\#}$
2,5-Di-tert-butylhydroquinone	2.28	$\uparrow^{\#}$	1-oleoyl-2-myristoyl-sn-glycero-3-phosphocholine	2.18	$\downarrow^{\#\#}$
Sulfamerazine	2.22	$\uparrow^{\#}$	Prostaglandin H2 (PGH2)	2.29	$\downarrow^{\#\#}$
Octanoylcarnitine (Car(8:0))	1.97	$\downarrow^{\#\#}$			

Arrows for metabolite expression changes: ↑ denotes upregulated metabolites in the PSO group relative to the Control, and ↓ denotes downregulated metabolites. $^{\#}p < 0.05$, $^{\#\#}p < 0.01$ vs. control group.

Supplementarey Table S7 PSO affects the relative expression levels of key serum metabolites.

Metabolite composition	Control	PSO
alpha-Linolenic acid	0.001591 ± 0.00018	$0.02585 \pm 0.00978^{\#\#}$
Eicosapentaenoic acid	$0.00244 \pm 7.01\times10^{-5}$	$0.00094 \pm 0.00052^{\#\#}$
Palmitic acid	0.01404 ± 0.00800	$0.00800 \pm 0.00105^{\#\#}$
Arachidonic acid (AA)	0.00891 ± 0.00180	$0.00605 \pm 0.00079^{\#\#}$
Prostaglandin H2 (PGH2)	$0.00030 \pm 2.00\times10^{-5}$	$0.00015 \pm 2.69\times10^{-5\ \#\#}$
Prostaglandin E2 (PGE2)	$0.00030 \pm 2.00\times10^{-5}$	$0.00015 \pm 2.69\times10^{-5\ \#\#}$
alpha-Tocopherol (Vitamin E)	$5.36\times10^{-5} \pm 1.21\times10^{-5}$	$3.52\times10^{-5} \pm 7.63\times10^{-6\ \#\#}$
Arachidonoylcarnitine (Car(20:4))	$4.80\times10^{-5} \pm 9.38\times10^{-6}$	$2.34\times10^{-5} \pm 2.79\times10^{-6\ \#\#}$
Palmitoylcarnitine (Car(16:0))	0.00091 ± 0.00016	$0.00051 \pm 9.55\times10^{-5\ \#\#}$

Data are presented as mean ± standard deviation. $^{\#\#}p < 0.01$ vs. control group.

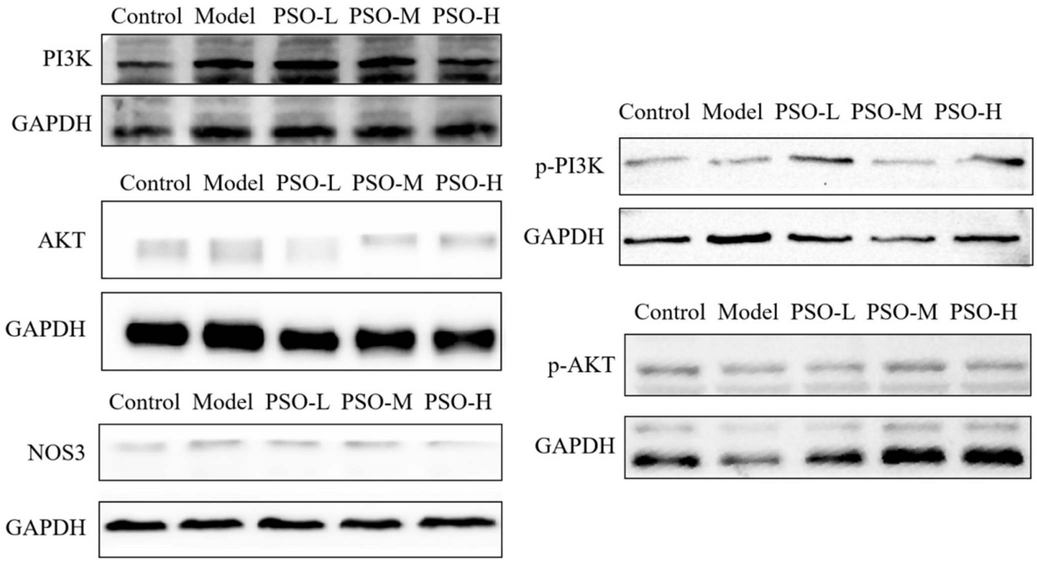

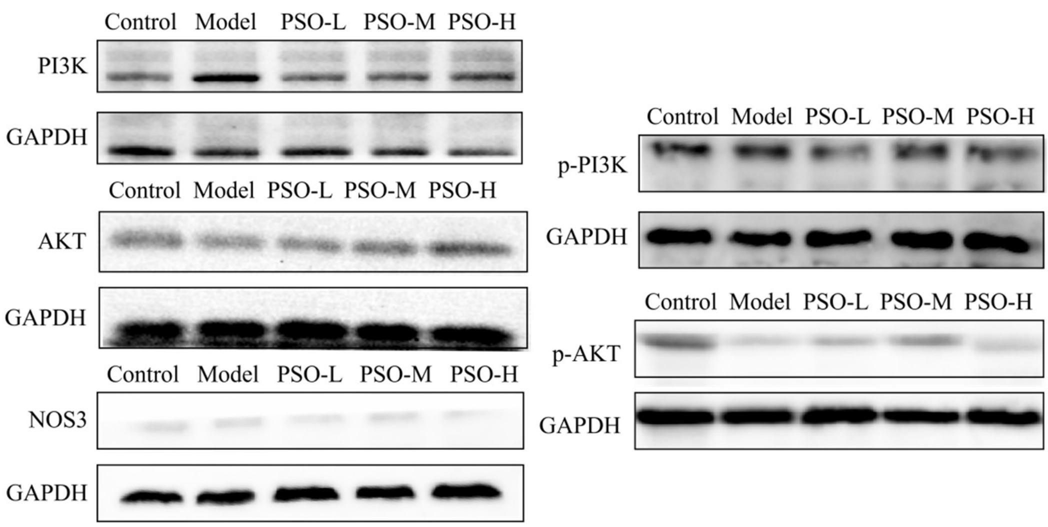

Supplementarey Figure S3. Western blot analysis of the expression of PI3K/AKT/NOS3 signaling pathway-related proteins.

Supplementarey Table S8 Expression levels of key liver proteins.

Group	p-PI3K/PI3K	p-AKT/AKT	NOS3/GAPDH
Control	0.68±0.10	0.85±0.08	0.29±0.07
Model	0.51±0.05 ##	0.63±0.06 ns	0.11±0.01 ##
PSO-L	1.17±0.19 **	0.61±0.07 ns	0.21±0.04 *
PSO-M	1.14±0.10 **	1.05±0.11**	0.28±0.02 **
PSO-H	1.06±0.22 **	1.06±0.09**	0.22±0.02 *

Data are presented as mean ± standard deviation (n = 3). ## $p < 0.01$ vs. control group; *$p < 0.05$, **$p < 0.01$ vs. model group. ns means p > 0.05 vs. control group or vs. model group.
